# Discovery of Highly Potent Noncovalent Inhibitors of SARS-CoV‑2 Main Protease through Computer-Aided Drug Design

**DOI:** 10.1021/acs.jmedchem.5c01199

**Published:** 2025-10-12

**Authors:** Atsutoshi Okabe, Daniel W. Carney, Michiko Tawada, Thamina Akther, Jumpei Aida, Terufumi Takagi, Douglas R. Dougan, Abba E. Leffler, Jeffrey A. Bell, Leah Frye, Eugene R. Hickey, Mallareddy Komandla, Will Tao, Jangir Selimkhanov, Kazuko Yonemori, Edcon Chang, Kumar Saikatendu, Atsuko Ochida

**Affiliations:** † 13498 Takeda Pharmaceutical Company Limited, 26-1 Muraoka-Higashi 2-chrome, Fujisawa, Kanagawa 251-8555, Japan; ‡ 638967 Takeda Development Center Americas, Inc., 9625 Towne Centre Drive, San Diego, California 92121, United States; § 428090 Schrödinger, Inc., 1540 Broadway, New York, New York 10036, United States; ∥ 428090 Schrödinger, Inc., 101 SW Main Street, Suite 1300, Portland, Oregon 97204, United States

## Abstract

The COVID-19 pandemic has highlighted a clear need to ensure rapid and equitable global access to health interventions in preparation for future coronavirus-driven pandemics. Here, we report the discovery of highly potent noncovalent inhibitors of the severe acute respiratory syndrome coronavirus 2 (SARS-CoV-2) main protease (Mpro) with pan-coronavirus (pan-CoV) Mpro inhibition through computer-aided drug design. Virtual screening led to the identification of a noncovalent hit compound with a piperazine core. Structure-guided scaffold morphing provided a novel trisubstituted piperidine core. Free energy perturbation (FEP)-guided designs, with induced-fit of Met49/Met165 and Gln189, resulted in the identification of highly potent compound **30**, which exhibits pan-CoV Mpro inhibition and cellular antiviral efficacy against the SARS-CoV-2 omicron variant. The optimized lead compound **30** was characterized by in vitro ADME/Tox assays and in vivo mouse pharmacokinetics. These findings suggest that compound **30** could be an addition to the repertoire of tools used to support future pandemic preparedness.

## Graphical abstract

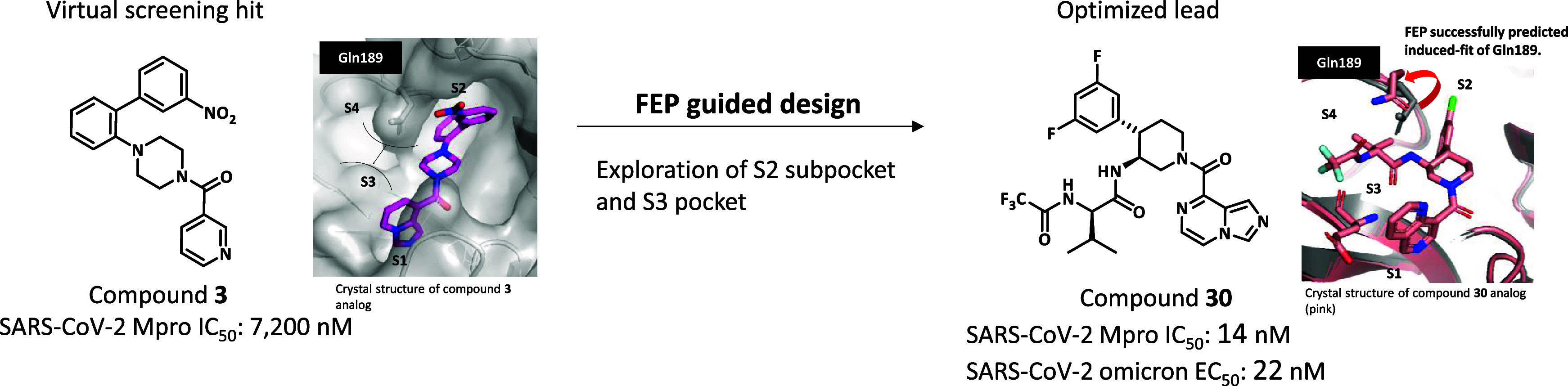

## Introduction

COVID-19 is a highly contagious infectious disease caused by the severe acute respiratory syndrome coronavirus 2 (SARS-CoV-2). Despite rapid development of vaccines, the virus has infected over 775 million people worldwide, resulting in 7 million deaths.^1^ In early 2020, Takeda attempted the development of a plasma-derived anti-SARS-CoV-2 polyclonal hyperimmune globulin (H-IG) to treat high-risk individuals with COVID-19 and explored the repurposing of marketed drugs. To address future pandemic preparedness, small-molecule drug development with pan-coronavirus (pan-CoV) antiviral activity was initiated with external collaborations.^2^

The main protease (Mpro) is essential for viral replication. The primary function is to cleave two large viral polypeptides into functional nonstructural proteins.^3,4^ Although Mpro is a highly conserved protease among various coronaviruses, there are no human proteases with a similar cleavage specificity.^5^ Therefore, Mpro is an attractive target for antiviral drug development for COVID-19 treatment. When our research program was initiated, the information on SARS-CoV-2 Mpro inhibitors was limited, and only covalent inhibitors such as ketoamide-based inhibitors and aldehyde-based inhibitors for SARS-CoV-1 Mpro had been reported.^6–8^ Since covalent inhibitors may cause unexpected toxicity due to their high reactivity, it is worthwhile to develop noncovalent inhibitors for SARS-CoV-2 Mpro.

Two oral drugs, nirmatrelvir and ensitrelvir, that target Mpro have been approved to treat COVID-19. However, both drugs have limitations due to drug–drug interactions due to CYP3A4 inhibition.^9–11^ In regard to pan-CoV Mpro inhibition, ensitrelvir has weak activity against the human coronaviruses MERS, 229E, and NL63, while nirmatrelvir has a broader spectrum of activity.^12^ In order to support future pandemic preparedness, there is a pressing need to develop next-generation noncovalent inhibitors with pan-CoV antiviral activity, which address the limitations regardless of currently available SARS-CoV-2 drugs.^13^

In order to find more effective pan-CoV Mpro inhibitors, we utilized free energy perturbation (FEP) calculation, which is a rigorous method for the prediction of binding affinity using molecular dynamics (MD) simulations, in conjunction with a structure-based design strategy. MD simulations take the conformational dynamics of a target protein into account, enabling FEP calculations to prioritize potent compounds with high accuracy, while accounting for induced-fit effects.^14,15^ Previously, FEP has been shown to work well for designing potent covalent Mpro inhibitors.^16^ In this article, we describe hit compound identification of SARS-CoV-2 Mpro inhibitors by virtual screening (VS). We focus on a novel noncovalent scaffold that was identified among the hit compounds. We also demonstrate rapid potency enhancement using prospective FEP calculations, which predicted induced conformational changes of amino acid residues within the S2 and S3 pockets upon ligand binding. Furthermore, we characterize the optimized lead compound for pan-CoV Mpro inhibition, cellular antiviral efficacy against SARS-CoV-2 omicron variant, as well as its in vitro ADME/Tox and in vivo mouse PK profiles.

## Results and Discussions

### Hit Compound Identification for SARS-CoV-2 Mpro Inhibitor by Virtual Screening

Structure- and ligand-based VS methods are common computational techniques for hit compound identification. As compared to traditional high-throughput screening, these techniques are complementary and effective in reducing the time and cost as well as identifying structurally diverse compounds.^17–19^ Both VS methods were used in the computational hit-finding approach (Figure 1A). In docking-based VS, multiple known crystal structures of SARS-CoV-2 Mpro as of early 2020 were used to consider protein flexibility. (See Experimental Section for details). In ligand-based VS, not only known SARS-CoV-2 Mpro inhibitors but also SARS-CoV-1 Mpro inhibitors were used as query compounds for similarity searches because SARS-CoV-1 Mpro inhibitors were also likely to inhibit SARS-CoV-2 Mpro due to the high sequence identity between them.^20^ A 1.5 M Takeda compound library was used for both structure- and ligand-based VS. After filtering by docking scores, similarity scores, and physicochemical properties for drug-likeness, 16,925 VS hit compounds were selected for the biochemical assay against the SARS-CoV-2 Mpro. We identified 21 hit compounds representing multiple structurally distinct scaffolds with IC_50_ < 30 μM. Next, we prioritized the three representative hit compounds (compounds **1–3**) for the hit-to-lead generation process (Figure 1B). Compound **1** was a known calpain inhibitor^21^ and served as a starting point for the rapid discovery of lead compounds that exhibited potent enzyme inhibitory activities and cellular antiviral activities with excellent pan-CoV coverage.^16^ Compound **2** was a peptide ketoamide inhibitor, which is known as a calpain inhibitor.^22^ The discovery of a derivative of compound **2** with a novel ketoamide core structure will be discussed in a future publication. Compound **3** was an attractive hit compound with moderate SARS-CoV-2 Mpro inhibition, which was a novel noncovalent inhibitor structurally distinct from well-known covalent inhibitors. In this article, we describe the drug discovery effort focusing on noncovalent compound **3**.

**Figure 1:**
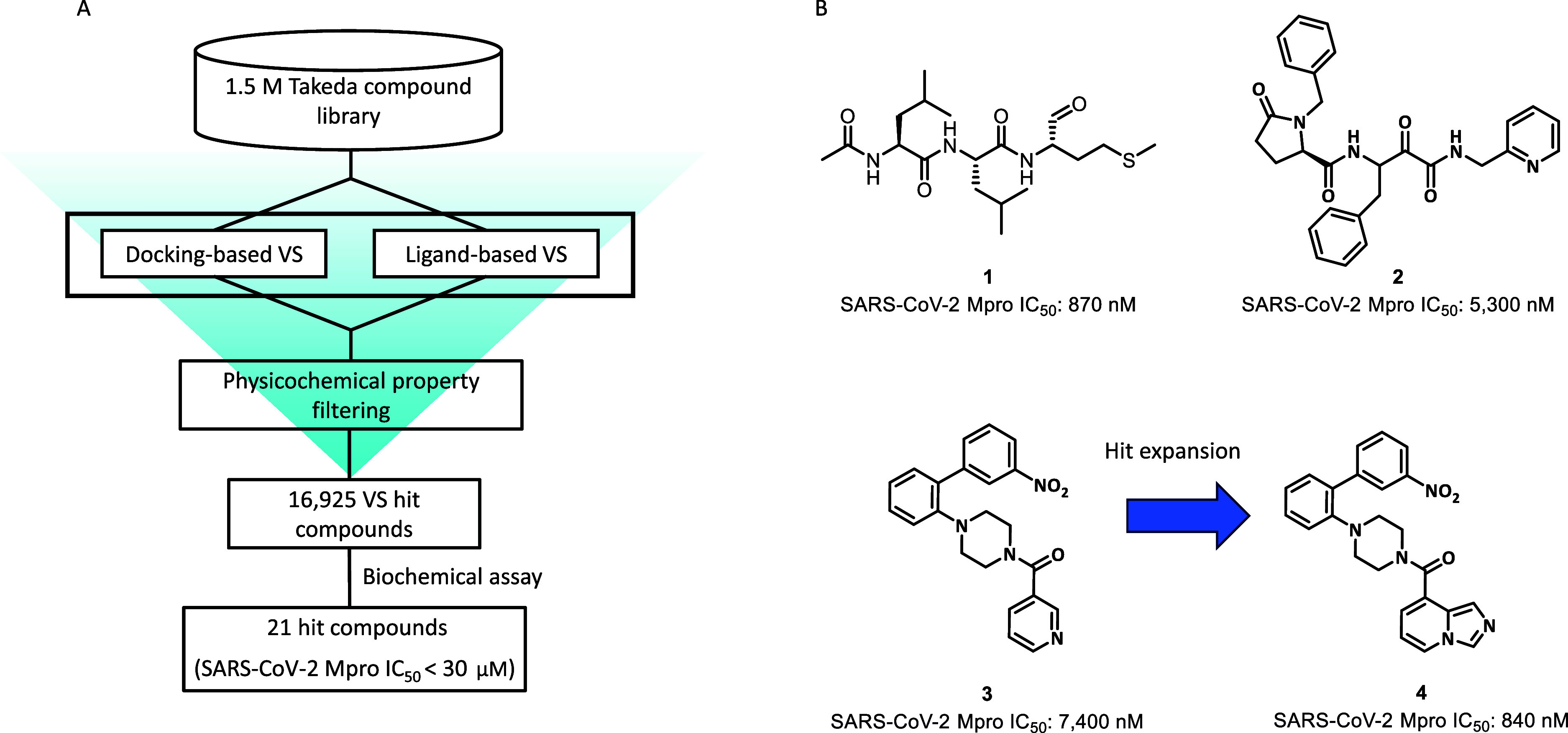
(A) VS workflow for hit compound identification. (B) Representative hit compounds (compounds **1–3**) and hit expansion of compound **3** to afford compound **4**.

### Hit Expansion and Structure-Based Drug Design Strategy (SBDD)

Initial SAR studies for hit expansion identified compound **4**, which demonstrated submicromolar inhibitory activity against SARS-CoV-2 Mpro (IC_50_ = 840 nM, Figure 1B) and showed potential pan-CoV Mpro inhibition, except for MERS (Table 1). In addition, compound **4** was highly selective against the human cathepsin B (H-CatB), which was used as a counter screen for promiscuous cysteine protease inhibition.

**Table 1:**
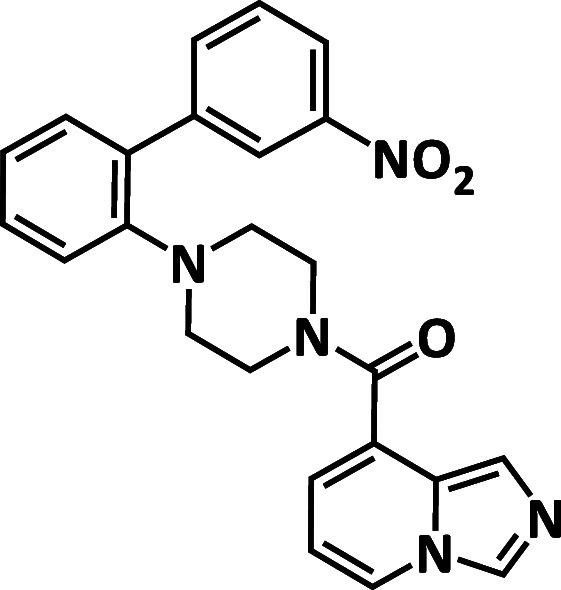
Pan-CoV Mpro Inhibition and Inhibitory Activity against Human Cathepsin B (H-CatB) of Compound **4**

	enzyme IC_50_ (nM)
SARS-CoV-2 Mpro	840
SARS-CoV-1 Mpro	620
MERS Mpro	>50,000
229E Mpro	1200
HKU1 Mpro	1000
NL63 Mpro	3700
0C43 Mpro	2300
H-CatB	>50,000

The SBDD was performed subsequently to improve pan-CoV Mpro inhibition. A crystal structure of the SARS-CoV-2 Mpro in complex with compound **4** was solved at a 1.4 Å resolution (Figure 2A). Crystallographic information suggested that the imidazopyridine moiety of compound **4** was bound in the S1 pocket with a hydrogen bond to His163, and the biaryl moiety was bound in the S2 pocket. A carbonyl linking the imidazopyridine to the central piperazine linker was positioned on the perimeter of the oxyanion hole, forming a direct hydrogen bond with Gly143 and a water-mediated contact with Cys145. The nitrophenyl moiety had van der Waals interaction with the side chain of Met49, while the phenyl moiety pointed toward solvent. This binding mode explains the SAR studies that the inhibitory activity against SARS-CoV-2 Mpro drastically decreased with the removal of the nitrophenyl moiety, while other modifications of this structural group did not have much impact on inhibition (Table S1, compounds **5–9**). The binding mode for compound **4** also helped explain the lack of MERS Mpro inhibition, likely due to the steric hindrance of the nitrophenyl moiety with Leu49 and Met25 in MERS (Figure 2B). Based on these observations, the design strategies to enhance pan-CoV Mpro inhibition were (1) to remove the nitrophenyl moiety to enable MERS Mpro inhibition and 2) to introduce substituents aiming at the S3 pocket to enhance pan-CoV Mpro inhibition (Figure 2C). Based on the overlay of crystal structures of SARS-CoV-2 Mpro in complex with compound **4** and the known peptide inhibitor and aldehyde inhibitor,^21,23^ P3 [using the Schechter–Berger nomenclature][Bibr ref24] alkyl and P3 aromatic series with an amide linker were designed at the 3 position on a piperidine core for chemical stability (Figure 2D,E).

**Figure 2:**
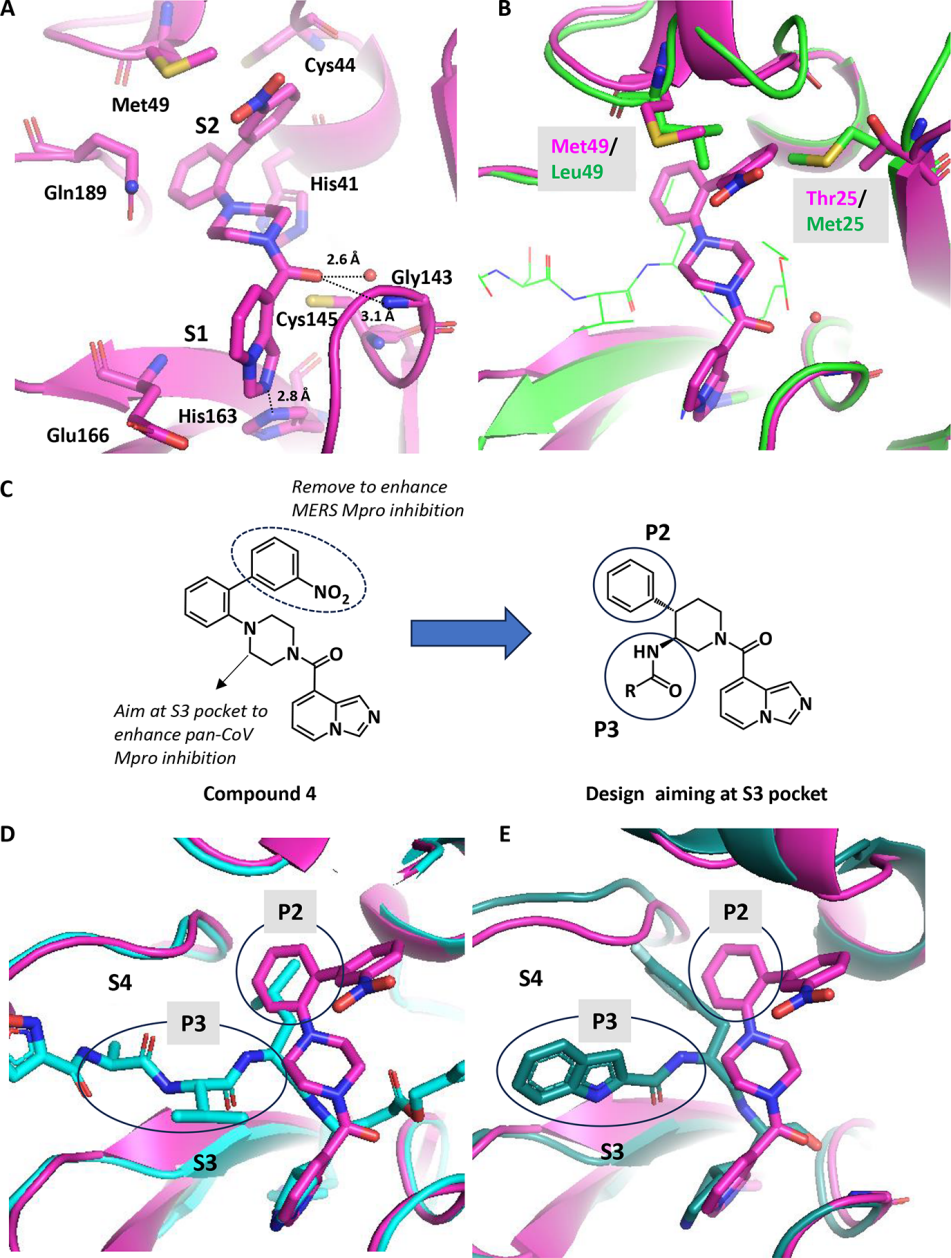
(A) Crystal structure of SARS-CoV-2 Mpro in complex with compound **4**. (B) Overlay of the crystal structure of SARS-CoV-2 Mpro in complex with compound **4** (magenta) and the crystal structure of MERS Mpro, showing steric clash of the nitrophenyl moiety with Leu49 and Met25 in MERS (green, PDB ID: 4RSP).^25^ (C) Design strategy to enhance MERS Mpro and pan-CoV Mpro inhibition. (D) Overlay of the crystal structure of SARS-CoV-2 Mpro in complex with compound **4** (magenta) and the crystal structure of SARS-CoV-2 in complex with a peptide inhibitor (cyan, PDB ID: 6LU7).^23^ (E) Overlay of the crystal structure of SARS-CoV-2 Mpro in complex with compound **4** (magenta) and the crystal structure of SARS-CoV-2 Mpro in complex with an aldehyde inhibitor (dark green, PDB ID: 8VQX).^16^

### FEP Calculations to Optimize Potency

#### Evaluation for Introducing P3 Substituents on the Piperidine Core

In the crystal structure of SARS-CoV-2 Mpro in complex with compound **4**, there was no apparent space to introduce substituents into the S3 pocket because of the conformational change of the Gln189 side chain (Figure 3A,B). Unsurprisingly, several designed analogs of compound **4** with P3 substituents failed to dock into the protein by ordinary Glide docking in which the protein structure is fixed. In order to evaluate if it was possible to introduce P3 substituents on the piperidine core that could cause induced-fit effects in the S3 pocket, we applied FEP calculations, as implemented in the FEP+ package (see Experimental Section for details). FEP calculations are a rigorous approach to predict protein–ligand binding affinity, accounting for induced-fit effects as part of the simulation.^14,15,26^

**Figure 3:**
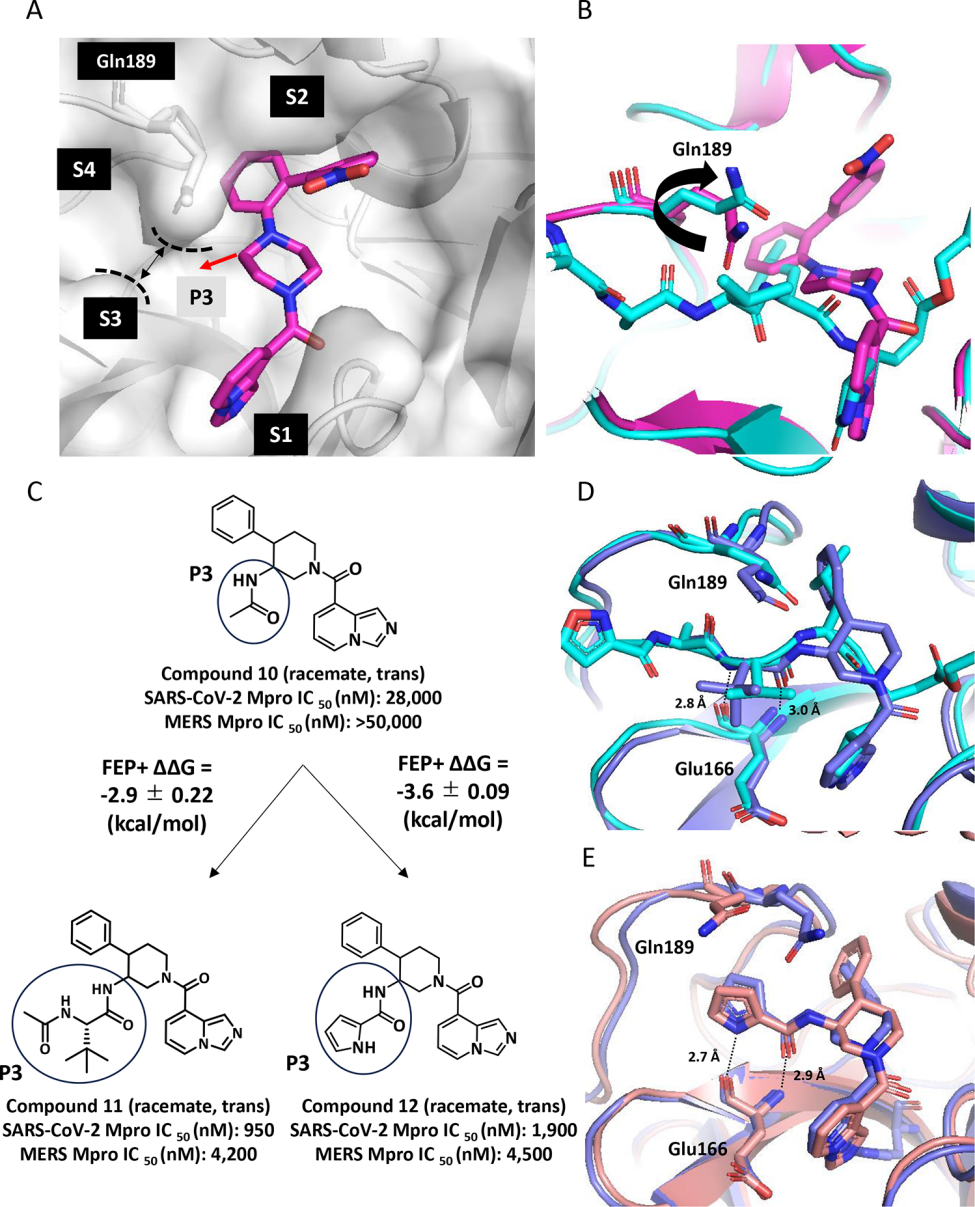
(A) Narrow space for accessing to the S3 pocket in the crystal structure of SARS-CoV-2 Mpro in complex with compound **4**. (B) Comparison of the conformation of the side chain of Gln189 in the crystal structure of SARS-CoV-2 Mpro in complex with compound **4** (magenta) and the known crystal structure of SARS-CoV-2 Mpro (PDB ID: 6LU7, cyan). (C) FEP+ calculations for design compounds with P3 substituents. (D) Overlay of the representative structure of SARS-CoV-2 Mpro in complex with compound **11** in FEP+ calculation (purple) and the crystal structure of SARS-CoV-2 Mpro in complex with a peptide inhibitor (PDB ID: 6LU7, cyan). (E) Overlay of the representative structure of SARS-CoV-2 Mpro in FEP+ calculation (purple) in complex with compound **12** and the crystal structure of SARS-CoV-2 Mpro in complex with compound **12** (pink).

In FEP+ calculations, compound **11** with the P3 alkyl moiety and compound **12** with the P3 aromatic moiety were predicted to improve the SARS-CoV-2 Mpro inhibition relative to compound **10** by inducing an induced-fit in the Gln189 side chain along with the formation of hydrogen bonds with the backbone of Glu166 (FEP+ ΔΔ*G* = −2.9 kcal/mol (**10** → **11**), −3.6 kcal/mol (**10** → **12**), Figure 3C–E). As expected, both compounds exhibited over 10-fold improvement in the SARS-CoV-2 Mpro inhibition compared to compound **10** as well as moderate MERS Mpro inhibition (**11** MERS Mpro IC_50_ = 4200 nM, **12** MERS Mpro IC_50_ = 4500 nM). Comparing the binding mode of compound **12** in the crystal structure with the one in the FEP+ calculation, a representative conformation of the side chain of Gln189 in the FEP+ calculation did not correspond to the one in the crystal structure (Figure 3E). This is probably because the side chain of Gln189 has large fluctuations and various conformations, as indicated from B-factors in the crystal structure and the FEP+ simulation (Figure S1). Although the P3 acetamide moiety of compound **10** had a binding mode similar to that of compounds **11** and **12**, the SARS-CoV-2 Mpro inhibition of compound **10** was weaker. It is likely that potency is significantly boosted by the addition of P3 substitutes that form a hydrogen bond with the backbone carbonyl oxygen of Glu166.

#### Exploration of Substituents on the P2 Phenyl Moiety to Optimize S2 Pocket Interaction

We next turned our attention to optimizing the P2 phenyl moiety to further improve the potency. Comparing the crystal structure of SARS-CoV-2 Mpro in complex with compound **12** to a known crystal structure of SARS-CoV-2 Mpro (PDB ID: 6LU7), we expected that side-chain rotamer changes on Met49 and Met165 could lead to the formation of small subpockets (Figure 4A) that could accommodate substituents in three positions (Figure 4B). Therefore, we used FEP+ to evaluate variously sized phenyl ring substituents in the context of a flexible binding pocket. Considering that the S2 pocket consisting of Met49, Met165, and His41 (Figure 4A) is hydrophobic, hydrophobic substituents at R^1^ (compounds **14**–**20**, Table 2) were initially evaluated using compound **13** as a reference ligand for FEP+ calculations. In this exploration, the P3 moiety was replaced from the electron-rich pyrrole ring to an imidazole ring to mitigate a concern of the oxidative metabolism and decrease the lipophilicity.^27^ We observed that a chlorine atom provided the lowest predicted binding free energy among them (compound **15**, FEP+ ΔΔ*G* = −2.6 kcal/mol). Next, keeping the chlorine atom at R^1^, the introductions of small substituents (F, Cl, and CN) at R^2^ or R^3^ were evaluated (compounds **21**-**26**). The 3,5-dichlorophenyl moiety (compound **21**, FEP+ ΔΔ*G* = −4.3 kcal/mol) and 3-chloro-5-fluorophenyl moiety (compound **23**, FEP+ ΔΔ*G* = −4.9 kcal/mol) had significantly lower predicted binding free energies. Following these predictions, compounds **15**, **21**, and **23** were synthesized. In line with the calculations, compound **15** showed over 5-fold improved potency, and compounds **21** and **23** showed about 100-fold improved potency compared to the reference compound **13** (SARS-CoV-2 Mpro IC_50_ = 170 nM (compound **15**), 21 nM (compound **21**), and 17 nM (compound **23**)).

**Figure 4:**
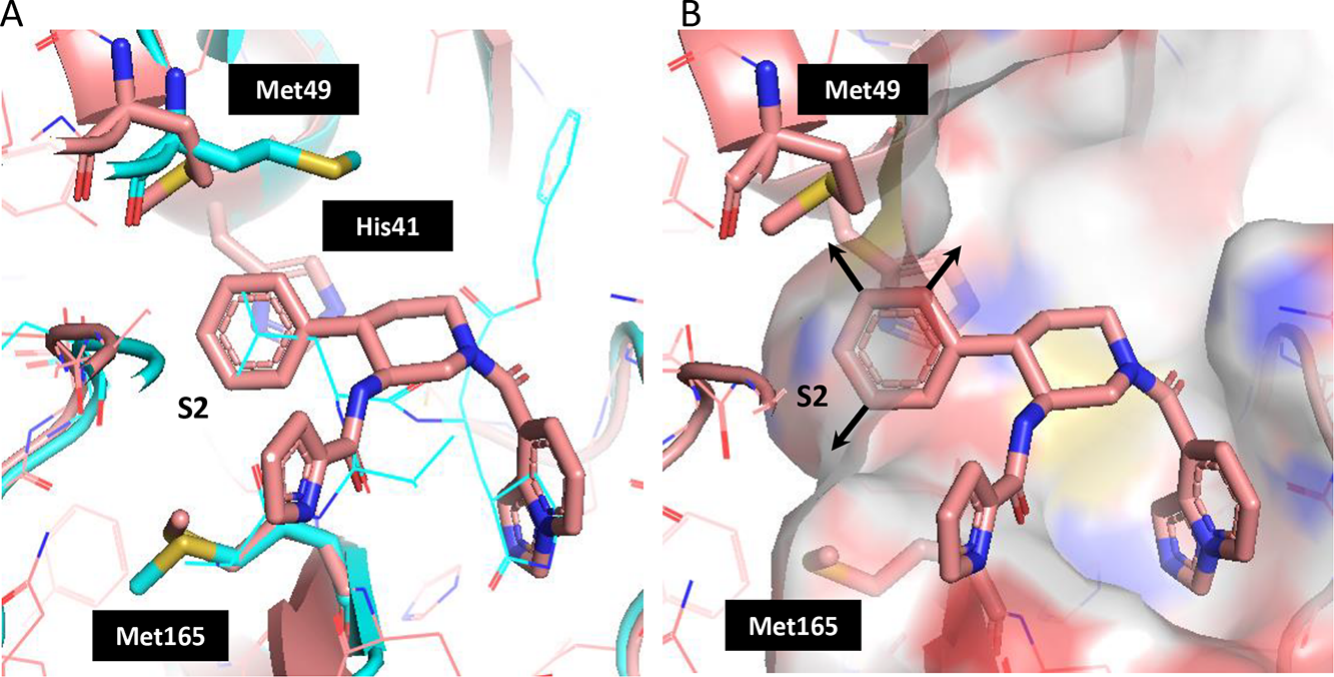
(A) Overlay of the crystal structure of SARS-CoV-2 Mpro in complex with compound **12** (pink) and the crystal structure of SARS-CoV-2 Mpro in complex with the peptide inhibitor (PDB ID: 6LU7, cyan). (B) Surface of the S2 pocket in the vicinity of the P2 phenyl group indicating possible substitution vectors. Corresponding substituent sites are indicated on the structure of FEP+ reference compound **13** (Table 2).

**Table 2:**
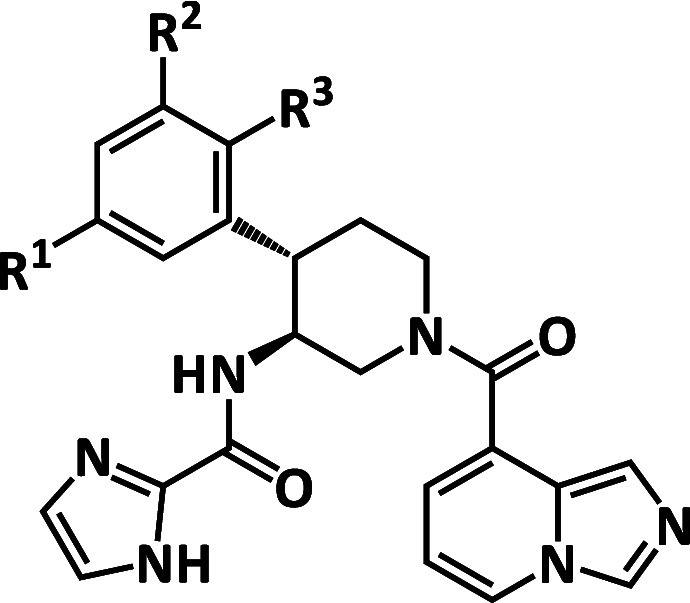
Exploration of the Subpocket to Boost Potency in the S2 Pocket

	R^1^	R^2^	R^3^	FEP+ (kcal/mol)	SARS-CoV-2 Mpro IC_50_ (nM)
13	H	H	H	0	1900
14	F	H	H	–1.4 ± 0.06	N.T.^a^
15^b^	CI	H	H	–2.6 ± 0.07	170
16	CN	H	H	–0.5 ± 0.08	N.T.
17	CH_3_	H	H	–0.6 ± 0.06	N.T.
18	CF_3_	H	H	1.3 ± 0.15	N.T.
19	Et	H	H	2.3 ± 0.10	N.T.
20	*n-*propyl	H	H	3.1 ± 0.13	N.T.
21	CI	F	H	–4.3 ± 0.06	21
22	CI	H	F	–2.6 ± 0.07	N.T.
23	CI	CI	H	–4.9 ± 0.08	17
24	CI	H	CI	–3.1 ± 0.09	N.T.
25	CI	CN	H	–3.0 ± 0.11	N.T.
26	CI	H	CN	–1.3 ± 0.15	N.T.

a N.T. means “not tested”,

b Racemate, trans.

#### Optimization of P3 Substituents

In general, drug candidates with fewer aromatic rings are likely to be easier to develop in terms of various developability parameters such as solubility, CYP inhibition, and hERG inhibition.^28^ Hence, we performed P3 alkyl series optimization while maintaining the 3-chloro-5-fluorophenyl moiety for the P2 group. Compound **27** was synthesized based on the SAR information that changing the P3 acetamide moiety of compound **11** to the P3 trifluoroacetamide moiety improved its membrane permeability (PAMPA *P*_app_, **11**: 3 nm/sec, CF_3_ analog: 131 nm/sec). Compound **27** demonstrated highly potent inhibitory activity against SARS-CoV-2 Mpro (IC_50_ < 12 nM). While it is unclear how much potency was improved for compound **27** compared to the P3 trifluoroacetamide analog (SARS-CoV-2 Mpro IC_50_ = 120 nM) of compound **11** because the IC_50_ value of compound **27** was below the detection limit of the biochemical assay, the introduction of both Cl atom on the 3 position and F atom on the 5 position of the P2 phenyl group even with the P3 alkyl group significantly improved the potency, which is consistent with the SAR, as shown in Table 2.

FEP+ calculations predicted that compound **28** with the P3 (*R*)-isomer would have a lower binding free energy than compound **27** with the P3 (*S*)-isomer (Figure 5A, FEP+ ΔΔ*G* = −3.6 kcal/mol). Comparing the predicted binding mode in the FEP+ calculation of compound **27** to compound **28** (Figure 5B,C), we attributed the predicted lower binding free energy of compound **28** to the following: (1) the side chain of Gln189 forms two hydrogen bonds with the P3 amide linker and the P3 trifluoroacetamide moiety, (2) the P3-(*R*) isopropyl moiety has better van der Waals interaction with the surrounding residues by occupying the S3 pocket more deeply, (3) the intramolecular interaction between the P3-(*R*) isopropyl group and the P2 phenyl group stabilizes the active conformation of compound **28**, and (4) the amide moiety of the P3-(*R*) isomer has water-mediated interactions with Glu166, while the P3-(*S*) isomer does not have the interaction due to the exposure of the hydrophobic isopropyl group (Figure 5D). Based on the FEP+ calculation, compound **28** was synthesized and showed higher cellular potency against the SARS-CoV-2 omicron variant (SARS-CoV-2 omicron EC_50_ = 130 nM (compound **27**), 28 nM (compound **28**)). No difference between the inhibitory activities of compound **27** and compound **28** against SARS-CoV-2 Mpro was observed and likely because the true potencies fall below the detection limit of the biochemical assay (Figure 5A). The overlay of the representative structure from the FEP+ calculation of compound **28** and the crystal structure of SARS-CoV-2 Mpro in complex with the analog compound **29** with the P1 naphthyridine group (SARS-CoV-2 Mpro IC_50_ < 12 nM) demonstrated that FEP+ calculations correctly predicted the induced-fit conformation of Gln189 (Figure 5E). Furthermore, compound **28** showed potent pan-CoV Mpro inhibition as well as high selectivity against several human cysteine proteases (caspase 2, cathepsin K, cathepsin L, and cathepsin S), serine proteases (chymotrypsin C, elastase, and thrombin), and aspartyl proteases (cathepsin D, pepsin) (Tables S2 and S3).

**Figure 5:**
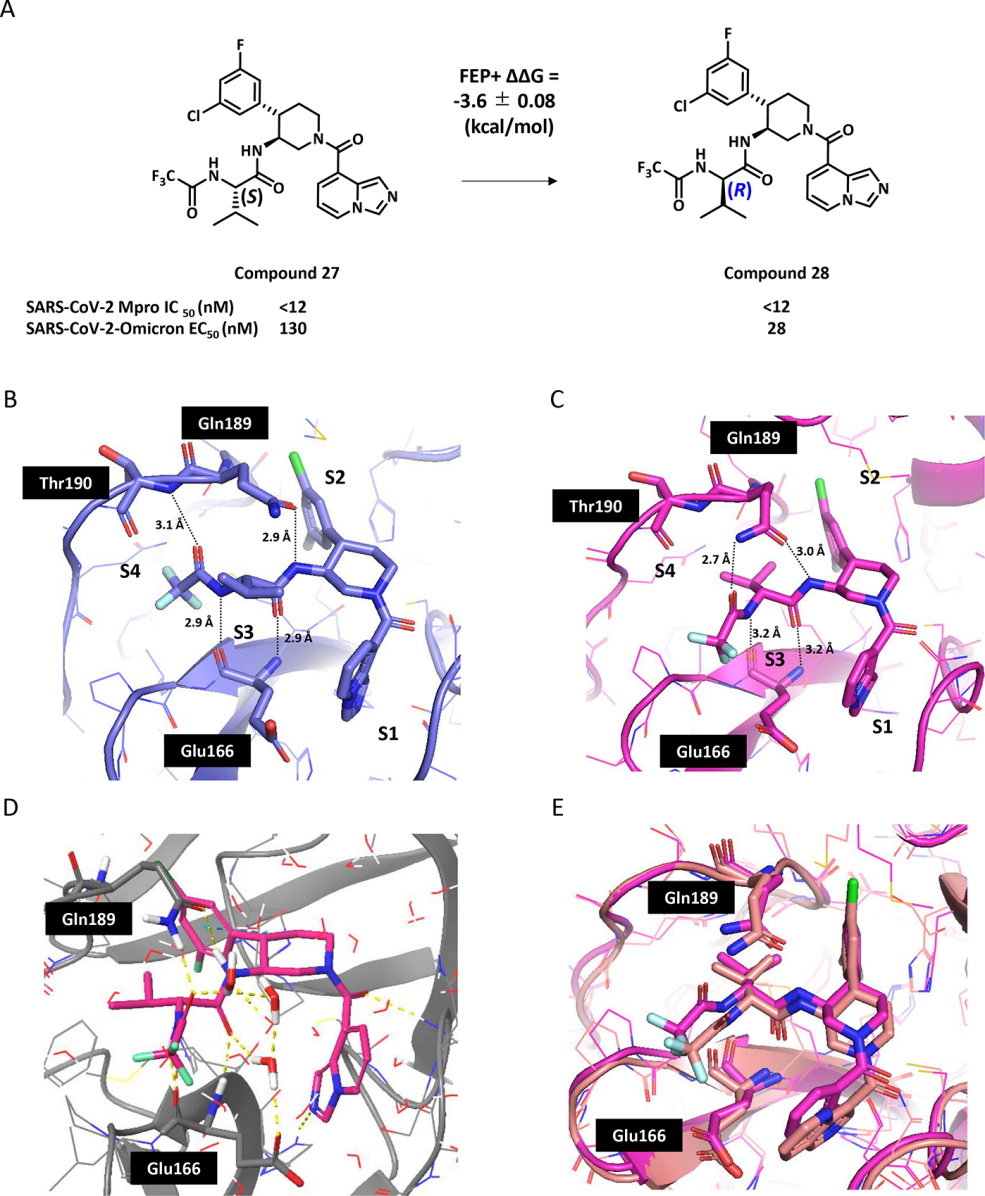
(A) Predicted binding free energy by FEP+ calculations and inhibitory activities of SARS-CoV-2 for compound **27** and compound **28**. (B), (C) Representative structure in the FEP+ calculation of compound **27** (purple) and compound **28** (magenta). Note the different rotamer states of the Gln189 side chain. (D) Predicted structure of compound **28** (magenta) with water-mediated interaction in FEP+ calculation. (E) Overlay of the representative structure in the FEP+ calculation of compound **28** (magenta) and the crystal structure of SARS-CoV-2 Mpro in complex with compound **29** (pink).

#### FEP+ Performance

Assessing FEP+ performance is valuable for quantifying the impact of prospective FEP+ calculations on this program. The utility of FEP+ was measured by calculating its overall prospective accuracy and throughput. In this study, FEP+ calculations for 7330 compounds were performed, of which 85 compounds were synthesized. All the FEP+ calculations (*n* = 85) were performed prior to synthesis and then compared to their subsequently determined experimental values. A strong, statistically significant association was observed between the predicted and experimental free energies, with Spearman’s ρ = 0.75 (*p* < 0.0001) (Figure 6A). In addition, the distributions of the predicted binding free energies for the synthesized and nonsynthesized compounds are significantly different (Figure 6B, unpaired *t* test, *p* < 0.0001). These results highlight the use of FEP+ potency predictions to prioritize potent compounds for synthesis, which in turn increased the efficiency of this study.

**Figure 6:**
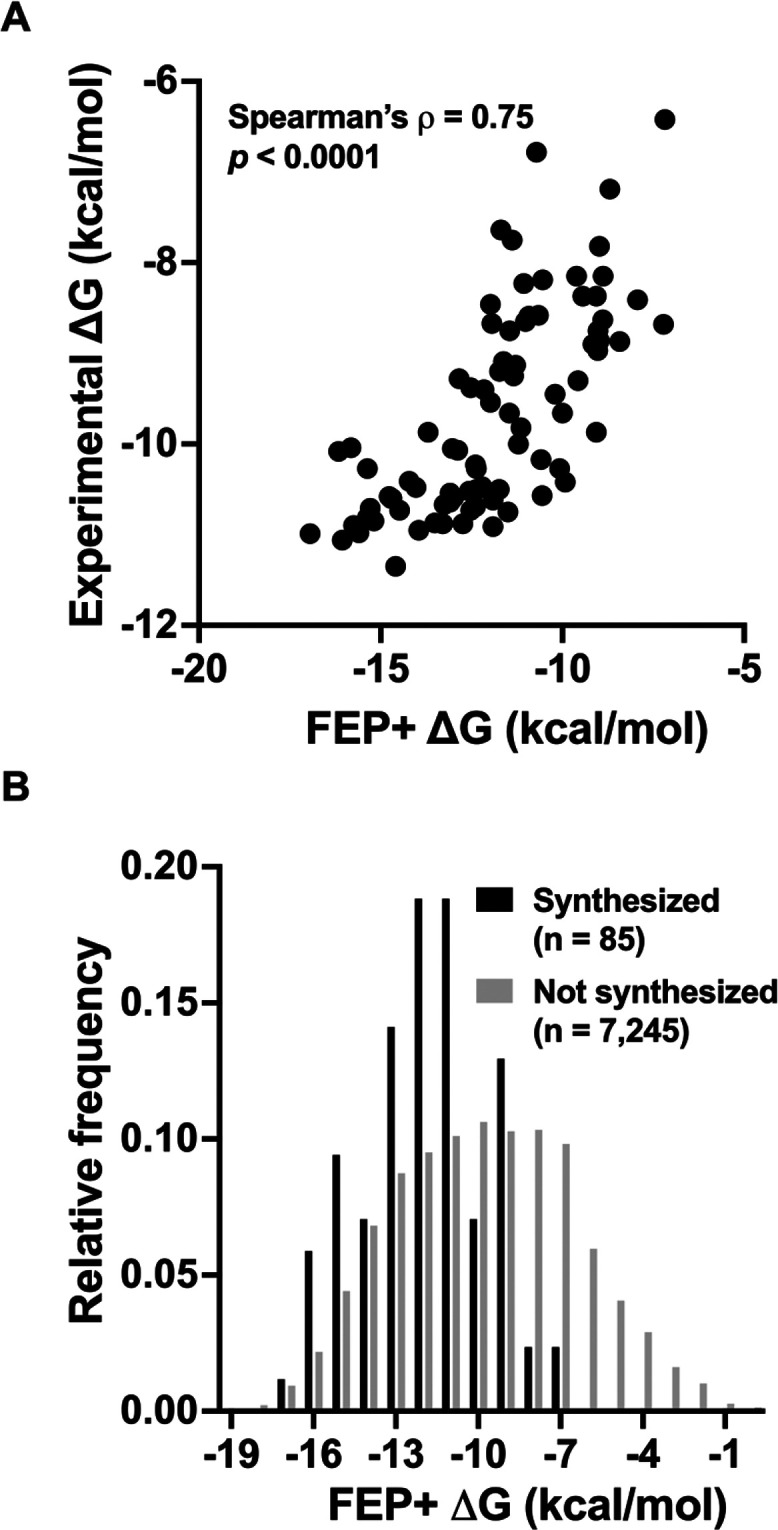
(A) Prospective performance of FEP+ calculations. (B) Distributions of FEP+ predicted binding free energies for compounds that were synthesized (black bars) and those that were not synthesized (gray bars). The experimental Δ*G* (binding free energy) was derived from the available experimental SARS-CoV-2 Mpro IC_50_ by using equation Δ*G*_exp_ ≈ *RT* ln (IC_50_).^29^

### Optimized Lead Compound 30

Although the potency of compound **28** was high, the metabolic stability in both human and mouse microsomes was low (Table 3). To improve the metabolic stability, compound **30** with a lower lipophilicity as compared to compound **28** was synthesized. Compound **30** demonstrated higher metabolic stability in both human and mouse microsomes as shown in Table 3.

**Table 3:**
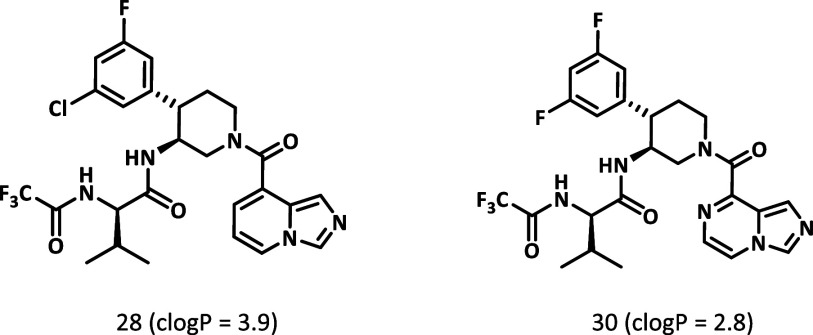
Improvement of Microsome Stability by Modification of P1 and P2 Moieties

	microsome stability (μL/min/mg)	
mouse	300	81
human	157	35

The in vitro pharmacology profile of compound **30** was characterized alongside those of nirmatrelvir and ensitrelvir (Table 4). Compound **30** showed a superior pan-CoV inhibition profile compared to ensitrelvir (Table 4A). The P1’ substituent of ensitrelvir, which is absent in compound **30** and nirmatrelvir, has interactions with the side chain of Met49 in SARS-CoV-2 Mpro.^30^ In MERS Mpro, the methionine is mutated to leucine, while in other human coronaviruses 229E Mpro and NL63 Mpro, the methionine is mutated to threonine (Figure S2). The side chains of leucine and threonine are shorter than methionine; hence, less van der Waal interaction with ensitrelvir is likely, which results in decreased potency against MERS, 229E, and NL63. In contrast, nirmatrelvir is known to have pan-CoV antiviral efficacy. This could be attributed to the peptide structure of nirmatrelvir mimicking the Mpro substrate, which is highly conserved across different coronaviruses.^31,32^ Thus, from the perspective of pan-CoV Mpro inhibition, this study demonstrated that it was advantageous that compound **30** binds to the S1, S2, and S3 pockets, but not to the S1’ pocket.

**Table 4:**
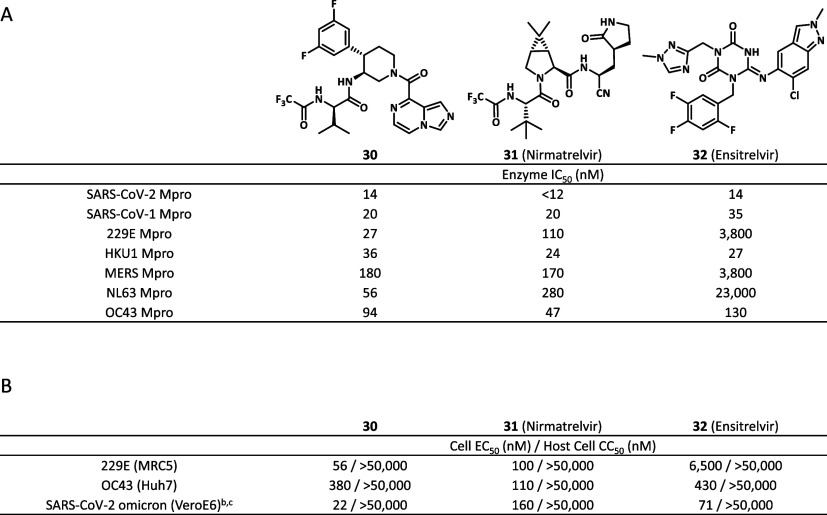
In Vitro Pharmacology Profile of Compound **30** and Marketed Inhibitors^a^

a (A) In vitro enzymatic inhibitory activities against human coronavirus Mpro. (B) In vitro cellular antiviral activities against human coronaviruses 229E, OC43, and SARS-CoV-2 omicron variant.

b Cellular activity in Vero E6 cells containing the P-glycoprotein (Pgp) inhibitor CP-100356.

c ln the assay, the mean and error for remdesivir as positive control are 47 ± 10 (nM).

Regarding cellular antiviral activity, compound **30** was potent against 229E, OC43, and SARS-CoV-2 omicron variant (Table 4B). Notably, compound **30** demonstrated approximately 8-fold improvement relative to nirmatrelvir and >3-fold improvement relative to ensitrelvir in the SARS-CoV-2 omicron variant (SARS-CoV-2 omicron EC_50_ = 22 nM (compound **30**), 160 nM (nirmatrelvir), and 71 nM (ensitrelvir)). The excellent cellular antiviral activity of compound **30** is likely due to the optimized interactions in the S2 pocket and the unique interaction of the P3-(*R*)-valine substituent in the S3 pockets.

Compound **30** had a favorable ADME/Tox profile with respect to CYP inhibition, cytotoxicity, and hERG inhibition (Tables 5A and Table S4). Compound **30** also demonstrated a clean safety profile with in vitro studies involving genotoxic potential (Table S4) and off-target selectivity (IC_50_ > 10 μM) against 47 targets with 78 readouts.^33^ On the other hand, pharmacokinetics studies in mouse revealed that oral bioavailability (F = 14%, Table 5B) may need improvement.

**Table 5:** In Vitro ADME/Tox Profile and In Vivo Mouse PK Profile of Compound **30** ^a^

**compound 30 ADME/Tox profile**	
thermodynamic solubility (μM) pH 1.2/6.8	1690/176
log *D* pH 7.4	2.2
PAMPA P_app_ (nm/sec)	73
MDCK-N1H-MDR1 P_app(a,b)_ (nm/sec)/ ER	<1.0/>37
PPB FU m/h	0.42/0.42
CYP %inh at 10 μM 1A2/2C8/2C9/2D6/3A4	–5.2/12/-2.0/34/29
HepG2 IC_50_ (μM) 72hGlu/24hGlu/24hGal	>100/>100/>100

**compound 30 mouse pharmacokinetics**	
IV Cl (mL/min/kg)	64
IV Vdss (L/kg)	0.64
IV t1/2 (h)	0.18
PO %F	14
PO *C*_max_ (ng/mL)	260
PO plasma AUC_0‑last_ (ng·h/mL)	1,100
PO lung AUC_0‑last_ (ng·h/mL)	160
AUC lung/plasma ratio	0.15

a PK measured in female C578*L*/6J mice, fed state, dosed at 1 mg/kg IV and 30 mg/kg PO.

## Chemistry

A general preparation of the piperidine-based Mpro inhibitors is illustrated by the synthetic route to compound **30** in Scheme 1. First, 3,5-difluorobenzaldehyde **33** was reacted with phosphanylidene **34** to generate the corresponding cinnamaldehyde **35** in 71% yield. Compound **35** was then condensed with *tert*-butyl *N*-(2-nitroethyl)­carbamate in the presence of the Hayashi-Jorgensen organocatalyst **37** ^34^ to deliver the dihydropyridine compound **38**. The organocatalyst-mediated conjugate addition and cyclization delivered products with high diastereoselectivity. In most cases, only the trans-diastereomer was isolated. Enantioselectivity was modest (data not shown) and variable, depending on the phenyl substitution pattern, which necessitated chiral separation at a later stage in the synthesis. It is likely that enantioselectivity could be improved through the optimization of the conjugate addition/cyclization reaction. However, we decided to forgo reaction optimization to rapidly progress multiple analogs through a conserved synthetic protocol.

**Scheme 1:**
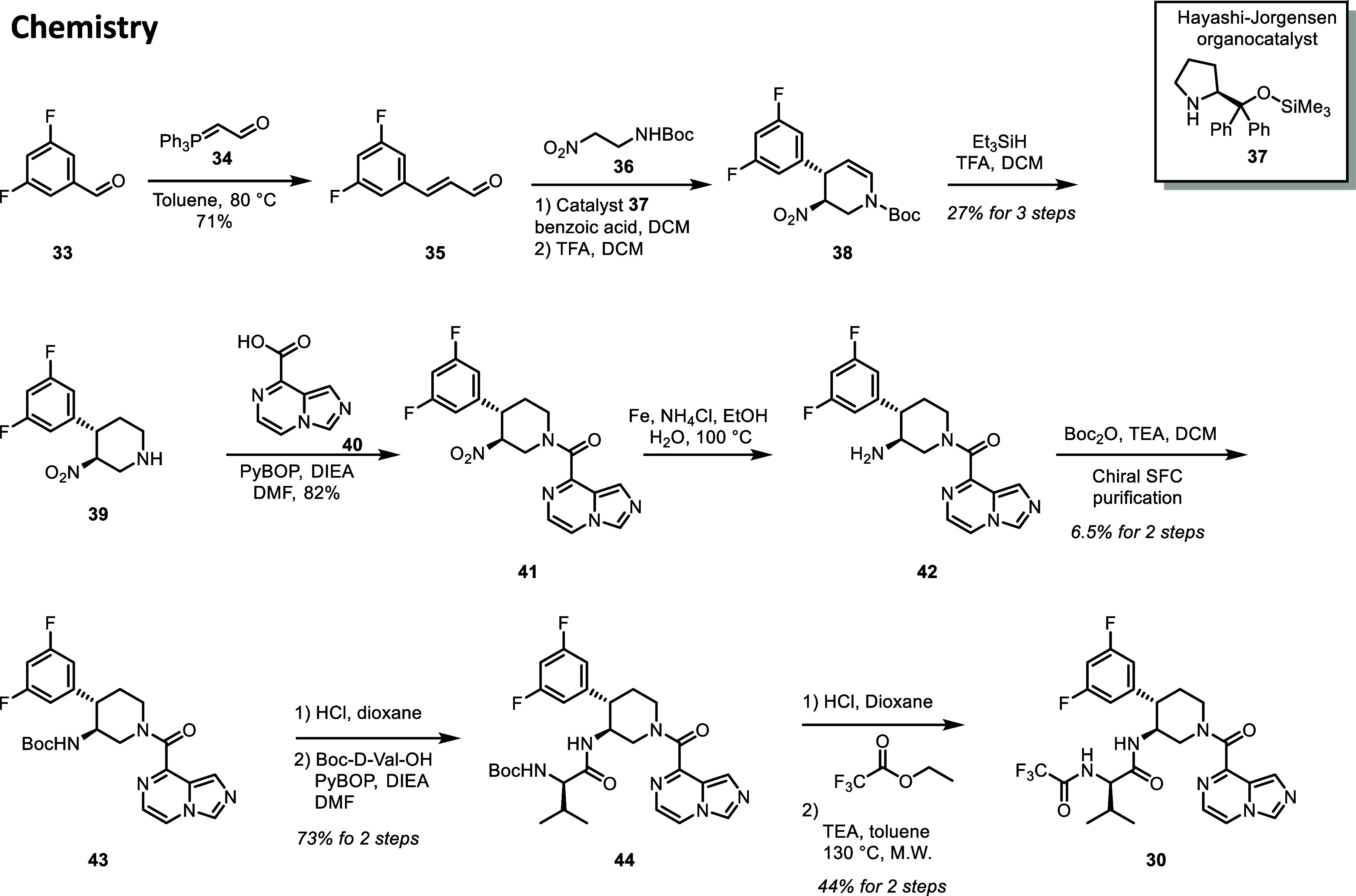
Synthesis of Compound **30**

Removal of the Boc-protecting group and reduction of the double bond in compound **38** were achieved in a single step by treatment with triethylsilane and trifluoroacetic acid in dichloromethane. The trans-disubstituted piperidine **39** was isolated in 27% yield from compound **35**. Next, the piperidine nitrogen was acylated by imidazo­[1,5-*a*]­pyrazine-8-carboxylic acid **40**, providing compound **41** in 82% yield. The nitro group in compound **41** was reduced with iron and ammonium chloride, and the resulting amine was protected with a Boc group to facilitate chiral separation. The bioactive 3S,4S enantiomer **43** was isolated in 6.5% yield for over steps from compound **41** after chiral SFC purification.

With the pure bioactive enantiomer of **43** in hand, the Boc-protecting group was removed with hydrochloric acid in dioxane, and the resulting amine was acylated with Boc-d-valine in 73% yield over two steps. Boc-deprotection, followed by trifluoroacetylation, delivered the final compound **30** in 44% yield over two steps.

## Conclusions

We identified a novel noncovalent SARS-CoV-2 Mpro inhibitor from the virtual screening. Hit-to-lead optimization was accomplished following structure-guided scaffold morphing and was accelerated by using FEP+ calculations. FEP+ calculations accurately predicted induced-fit conformations of Met49/Met165 (S2 pocket) and Gln189 (S3 pocket), which guided the optimization of P2 and P3 moieties resulting in the identification of highly potent compound **30** with pan-CoV Mpro inhibition. Further optimization of this chemical series to deliver a clinical candidate will focus on improving metabolic stability and permeability to maximize oral absorption and plasma half-life. This noncovalent chemical series is structurally differentiated from the marketed Mpro inhibitors (nirmatrelvir and ensitrelvir) and shows higher cellular potency against the SARS-CoV-2 omicron variant (8-fold vs nirmatrelvir, > 3-fold vs ensitrelvir). Compound **30** could be an important addition to the repertoire of tools to address potential drug resistance development and future pandemic preparedness.

## Experimental Section

### Chemistry

#### General Procedures

Ensitrelvir and nirmatrelvir were synthesized as described in the literature.^30,32^ The experimental procedures for the synthesis of compounds **3–29** are described in the Supporting Information. Concentrations were performed under vacuum on a Büchi rotary evaporator. Silica gel flash purifications were run on Biotage Isolera instruments using solvents as described below. Preparative HPLC was run on a SHIMADZU LH-40&MS-2020 instrument using columns and solvents as described below. Analytical HPLC was performed in one of six methods as described in the Supporting Information. NMR was run on a Bruker/AVANCE NEO 400 MHz. NMR δ values were reported in ppm. The purities of compounds submitted for biological evaluation were >95% as determined by analytical HPLC, unless otherwise noted. Reagents and materials were obtained from commercial suppliers and used without additional purification. Reactions were run in anhydrous solvent under ambient atmosphere, unless otherwise noted.

#### (*E*)-3-(3,5-Difluorophenyl)­acrylaldehyde (35)

A mixture of 3,5-difluorobenzaldehyde (5.00 g, 35.2 mmol) and 2-(triphenyl-λ^5^-phosphanylidene)­acetaldehyde (10.7 g, 35.2 mmol) in toluene (30 mL) was degassed and purged with N_2_. The mixture was stirred at 80 °C for 12 h under a N_2_ atmosphere. The reaction mixture was concentrated directly, and the product was purified by silica gel flash chromatography (4% EtOAc in PE). The title compound (4.20 g, 71% yield) was obtained as a light yellow solid. ^1^H NMR (DMSO-*d*_6_, 400 MHz) δ_H_ = 9.69 (d, *J* = 7.6 Hz, 1H), 7.74–7.67 (m, 1H), 7.60–7.52 (m, 2H), 7.41–7.33 (m, 1H), 7.03–6.95 (m, 1H).

#### (*trans*)-*tert*-Butyl 4-(3,5-difluorophenyl)-3-nitro-3,4-dihydropyridine-1­(2*H*)-carboxylate (38)

To a mixture of *tert*-butyl *N*-(2-nitroethyl)­carbamate (7.69 g, 40.4 mmol) in DCM (80 mL) were added benzoic acid (494 mg, 4.24 mmol), (*S*)-2-(diphenyl­((trimethylsilyl)­oxy)­methyl)­pyrrolidine (526 mg, 1.62 mmol), and (*E*)-3-(3,5-difluorophenyl)­acrylaldehyde (3.40 g, 20.2 mmol). The mixture was stirred at room temperature for 10 h. The mixture was diluted with DCM (90 mL) and cooled to 0 °C. TFA (5.70 g, 50.0 mmol) was added, and the mixture was stirred at room temperature for 5 h. The mixture was diluted with DCM (200 mL) and washed with saturated aqueous NaHCO_3_ (200 mL x 2). The organic layer was dried over Na_2_SO_4_ and concentrated to give the crude product (11.2 g, crude) as a brown/black oil, which was used in the next step without purification.

#### (*trans*)-4-(3,5-Difluorophenyl)-3-nitropiperidine (39)

To a solution of (*Trans*)-*tert*-butyl 4-(3,5-difluorophenyl)-3-nitro-3,4-dihydropyridine-1­(2*H*)-carboxylate (11.2 g, crude) in DCM (50 mL) were added Et_3_SiH (7.65 g, 65.8 mmol) and TFA (22.5 g, 197 mmol) at 0 °C. The mixture was stirred at room temperature for 3 h. The mixture was concentrated to give a crude residue, which was diluted with water (150 mL) and extracted with PE (100 mL x 3). The pH of the aqueous phase was adjusted to 8 with saturated aqueous NaHCO_3_ and then was extracted with DCM (200 mL x 3). The combined organic extracts were dried over anhydrous Na_2_SO_4_ and concentrated to a crude residue. The product was purified by silica gel flash chromatography (56% EtOAc in PE). The title compound (2.70 g, 27% yield over two steps) was obtained as a blackish brown oil. ESI-MS [M + H]^+^ calculated. for C_11_H_12_F_2_N_2_O_2_: 387.1, found 386.1.

#### [(*trans*)-4-(3,5-Difluorophenyl)-3-nitro-1-piperidyl]-imidazo­[1,5-*a*]­pyrazin-8-yl-methanone (41)

To a solution of (*trans*)-4-(3,5-difluorophenyl)-3-nitropiperidine (2.00 g, 33.0 mmol) and imidazo­[1,5-*a*]­pyrazine-8-carboxylic acid (942 mg, 5.78 mmol) in DMF (10 mL) were added PyBOP (6.45 g, 12.4 mmol) and DIEA (3.20 g, 24.8 mmol). The mixture was stirred at room temperature for 2 h. The mixture was diluted with water (50 mL) and extracted with EtOAc (50 mL x 3); then, the combined organic extracts were washed with brine (25 mL x 2), dried over Na_2_SO_4_, and concentrated. The product was purified by silica gel flash chromatography (88% EtOAc in PE). The title compound (2.87 g, 82%) was obtained as a yellow oil. ESI-MS [M + H]+ calculated. for C_18_H_15_F_2_N_5_O_3_ 388.1, found 388.2.

#### [(*trans*)-3-Amino-4-(3,5-difluorophenyl)-1-piperidyl]-imidazo­[1,5-*a*]­pyrazin-8-yl-methanone (42)

To a solution of [(*trans*)-4-(3,5-difluorophenyl)-3-nitro-1-piperidyl]-imidazo­[1,5-*a*]­pyrazin-8-yl-methanone (2.77 g, 7.15 mmol) in water (8 mL) and EtOH (25 mL) were added Fe (1.60 g, 28.6 mmol) and NH_4_Cl (1.53 g, 28.6 mmol). The mixture was stirred at 100 °C for 2 h. The mixture was filtered through a Celite pad and washed with DCM/MeOH (1:1, 300 mL x 3). The filtrate was concentrated, and the crude product (4.3 g, crude) was used without further purification. ^1^H NMR (DMSO-*d*_6_, 400 MHz) δ_H_= 8.69–8.67 (s, 1H), 8.45–8.41 (m, 1H), 7.94–7.92 (d, *J* = 8.0 Hz, 1H),7.63–7.59 (m, 1H), 7.15–7.12 (m, 3H), 4.06–4.00 (m, 1H), 3.03–2.89 (m, 7H), 1.91–1.71 (m, 2H).

#### *tert*-Butyl ((3*S*,4*S*)-4-(3,5-difluorophenyl)-1-(imidazo­[1,5-*a*]­pyrazine-8-carbonyl)­piperidin-3-yl)­carbamate (43)

To a solution [(*trans*)-4-(3,5-difluorophenyl)-3-nitro-1-piperidyl]-imidazo­[1,5-*a*]­pyrazin-8-yl-methanone (4.20 g, crude) in DCM (45 mL) were added Boc_2_O (5.13 g, 23.5 mmol) and TEA (3.57 g, 35.3 mmol). The mixture was stirred at room temperature for 12 h. Water (50 mL) was added to the mixture. The resulting mixture was extracted with DCM (100 mL x 3). The combined organic phase was dried over anhydrous Na_2_SO_4_, filtered, and concentrated. The product was first purified by silica gel column chromatography (95% EtOAc in PE), followed by chiral SFC (column: DAICEL CHIRALPAK AD (250 mm × 30 mm, 10um); mobile phase: [0.1%NH_3_H_2_O IPA]; B%: 35%–35%, min). The title compound (351 mg, 6.5%) was obtained as a yellow solid.

#### *tert*-Butyl ((*R*)-1-(((3*S*,4*S*)-4-(3,5-difluorophenyl)-1-(imidazo­[1,5-*a*]­pyrazine-8-carbonyl)­piperidin-3-yl)­amino)-3-methyl-1-oxobutan-2-yl)­carbamate (44)

To a solution of *tert*-butyl ((3*S*,4*S*)-4-(3,5-difluorophenyl)-1-(imidazo­[1,5-*a*]­pyrazine-8-carbonyl)­piperidin-3-yl)­carbamate (351 mg, 0.76 mmol) in DCM (4 mL) was added HCl/dioxane (4 M, 11 mL). The mixture was stirred at room temperature for 2 h. The mixture was concentrated directly, and the crude deprotected intermediate (443 mg, crude) was used in the next step without purification.

To a solution of the crude deprotected intermediate (40 mg, crude) and Boc-d-Val-OH (24 mg, 0.11 mmol) in DMF (1 mL) were added DIEA (72 mg, 0.56 mmol) and PyBOP (87 mg, 0.17 mmol) at 0 °C. The mixture was stirred at room temperature for 1 h. Water (15 mL) was added, and the reaction mixture was extracted with EtOAc (20 mL x 3). The combined organic extracts were washed with brine (20 mL x 3), dried over anhydrous Na_2_SO_4_, and concentrated. The product was purified by silica gel flash chromatography (94% EtOAc in PE). The title compound (50 mg, 73% yield over two steps) was obtained as a yellow solid. ESI-MS [M + H]^+^ calculated. for C_28_H_34_F_2_N_6_O_4_: 557.3, found: 557.2.

#### (*R*)-N-((3*S*,4*S*)-4-(3,5-Difluorophenyl)-1-(imidazo­[1,5-*a*]­pyrazine-8-carbonyl)­piperidin-3-yl)-3-methyl-2-(2,2,2-trifluoroacetamido)­butanamide (30)

To a mixture of *tert*-butyl ((*R*)-1-(((3*S*,4*S*)-4-(3,5-difluorophenyl)-1-(imidazo­[1,5-*a*]­pyrazine-8-carbonyl)­piperidin-3-yl)­amino)-3-methyl-1-oxobutan-2-yl)­carbamate (50 mg, 0.09 mmol) in DCM (1 mL) was added HCl/dioxane (4 M, 1 mL). The mixture was stirred at room temperature for 1 h. The reaction mixture was concentrated directly, providing the crude deprotected intermediate as a yellow solid, which was used in the next step without purification.

The crude deprotected intermediate (40 mg, crude), ethyl 2,2,2-trifluoroacetate (125 mg, 0.88 mmol), and TEA (53 mg, 0.53 mmol) were taken up into a microwave tube in toluene (1 mL). The sealed tube was heated to 130 °C for 4 h under microwave irradiation. The reaction mixture was concentrated directly, and the product was purified by prep-HPLC (column: Welch Xtimate C18 150 × 30 mm x 5 um; mobile phase: [water (NH_4_HCO_3_)-ACN]; B%: 18%–58%, 36 min). The title compound (22.1 mg, 44% yield over 2 steps, 97.2% purity) was obtained as a yellow solid. ^1^H NMR (MeOD, 400 MHz) δ_H_ = 8.59 (d, *J* = 4.4 Hz, 1H), 8.33–8.27 (m, 1H), 7.92 (d, *J* = 13.2 Hz, 1H), 7.65–7.59 (m, 1H), 7.00–6.92 (m, 2H), 6.83–6.75 (m, 1H), 4.32–3.82 (m, 3H), 3.30–2.71 (m, 4H), 2.11–1.65 (m, 3H), 0.74–0.59 (m, 3H), 0.51–0.38 (m, 3H). ESI-MS [M + H]^+^ calculated. for C_25_H_25_F_5_N_6_O_3_: 553.2, found: 553.2.

#### Main Protease Molecular Biology, Protein Expression, and Purification for Biochemical Assays

Mpro proteins were cloned, expressed in *E. coli*, and purified by WuXi AppTec Co., Ltd. Proteins were expressed in *E. coli* BL21 transformed with pGEX-6p-1 expression vectors containing the Mpro sequence within the BamH I/Xho I insertion site. Transformants were grown in Terrific Broth containing ampicillin at 200 rpm in a shaker overnight. Cell pellets were suspended using lysis buffer (20 mM Tris pH 8.0, 300 mM NaCl, 5% glycerol, 5 mM imidazole, 0.2 mM TCEP, 0.2 mM PMSF) supplemented with one protease inhibitor cocktail tablet and benzonase and disrupted by ultrasonication at 4 °C, followed by centrifugation at 48000 g for 40 min. The supernatant was passed through a 0.45 μm filter and loaded on a 5 mL HisTrap FF crude column. The column was washed with lysis buffer; then, the protein was eluted with elution buffer (20 mM Tris pH 8.0, 300 mM NaCl, 5% glycerol, 300 mM imidazole, 0.2 mM TCEP, 0.2 mM PMSF). The protein was then digested by Pierce HRV 3C Protease to remove the C-terminal his-tag in dialysis buffer (20 mM Tris, pH8.0, 300 mM NaCl, 5% glycerol, 0.2 mM TCEP) overnight. Postdigestion protein was further purified using a 5 mL HisTrap FF column collecting the flow-through washing with lysis buffer and elution buffer. The target Mpro was then purified from the flow-though by SEC using a HiLoad G200 column eluting with SEC buffer (20 mM Tris pH7.5, 100 mM NaCl, 20% glycerol, 1 mM TCEP). The target Mpro protein was validated on NuPage gels from samples of the collected SEC peaks. Purified target proteins were concentrated by using a spin concentrator at 4 °C, aliquoted, and stored at −80 °C.

#### Main Protease Substrates

Main protease substrates were synthesized by GenScript Biotech, and the substrates were dissolved in 10 mM stock in DMSO. SARS-CoV-2, SARS-CoV-1, MERS, HCoV-OC43, and HCoV-HKU1: Dabcyl-KTSAVLQSGFRKM-(Edans), HCoV-229E: Dabcyl-YGSTLQAGLRKM-(Edans), HCoV-NL63: Dabcyl-YNSTLQSGLKKM-(Edans).

#### Main Protease Biochemical Assays for Wild-Type

Compounds were three-fold serially diluted for 10 concentrations and added to an assay plate (384-well format) using ECHO, in duplicate wells. Two μM of GC376 was used as 100% inhibition control, and DMSO was used as the no inhibition control. Twenty-five μL of Mpro proteins were added to the assay plates containing compounds. The compounds and Mpro proteins were preincubated at room temperature for 30 min. Then, 5 μL of the Mpro substrate was added to the appropriate assay plates. The final concentrations of the protein and substrate were: 25 nM SARS-CoV-2 Mpro +25 μM substrate, 50 nM HCoV-229E Mpro +12.5 μM substrate, 50 nM HCoV-NL63 12.5 μM substrate, 25 nM SARS-CoV-1 Mpro +25 μM substrate, 25 nM HCoV-OC43 Mpro +12.5 μM substrate, 12.5 nM HCoV-HKU1 Mpro +12.5 μM substrate, or 100 nM MERS Mpro +25 μM substrate.

Each activity testing point had a relevant background control to normalize the fluorescence interference of the compound. After 60 min of incubation at 30 °C, the fluorescence signal (RFU) was detected using a microplate reader SpectraMax M2e (Molecular Devices) at Ex/Em = 340 nm/490 nm. The inhibition activity was calculated using the formula: Inhibition% = [(CPD-BGCPD)-(ZPE -BGZPE)/ (HPE-BGHPE)-(ZPE -BGZPE)] x 100%, where CPD: signal of test compounds wells, containing compound + enzyme + substrate; ZPE: average of signals of zero percent effective control wells, containing enzyme + substrate, no compound; HPE: average of signals of hundred percent effect control wells, containing GC376 + enzyme + substrate; and BG: compound background control wells, containing compound + substrate and no enzyme. The IC_50_ values of compounds were calculated with GraphPad Prism software using the nonlinear regression model of a log­(inhibitor) vs response–variable slope (four parameters).

#### Cellular Infectivity Assays for Wild-Type

SARS-CoV-2/HeLa-ACE2 infectivity was measured by using a high-content screening assay performed by Calibr, a division of the Scripps Research Institute. Compounds were acoustically transferred into 384-well clear-bottom plates (Greiner, Part. No. 781090–2B), and HeLa-ACE2 cells were seeded in the plates in 2% FBS at a density of 1.0 × 103 cells per well. Plated cells were transported to the BSL3 facility, where SARS-CoV-2 (strain USA-WA1/2020 propagated in Vero E6 cells) diluted in assay media was added to achieve ∼ 30 to 50% infected cells (MOI ∼ 1). Plates were incubated for 24 h at 34 °C and 5% CO2 and then fixed with a final concentration of 4% formaldehyde. Fixed cells were stained with human polyclonal sera as the primary antibody, goat antihuman H+L conjugated Alexa 488 (Thermo Fisher Scientific A11013) as the secondary antibody, and antifade-46-diamidino-2-phenylindole (DAPI; Thermo Fisher Scientific D1306) to stain DNA, with PBS 0.05% Tween 20 washes between fixation and subsequent primary and secondary antibody staining. Plates were imaged using the ImageXpress Micro Confocal High-Content Imaging System (Molecular Devices) with a 10× objective, with four fields imaged per well. Images were analyzed using the Multi-Wavelength Cell Scoring Application Module (MetaXpress), with DAPI staining identifying the host-cell nuclei (the total number of cells in the images) and the SARS-CoV-2 immunofluorescence signal, leading to the identification of infected cells.

Compounds were counter-screened for cytotoxicity in uninfected cells. Compounds were acoustically transferred into 1,536-well plates (Corning No. 9006BC). HeLa-ACE2 cells were maintained as described for the infection assay and seeded in the assay-ready plates at 400 cells/well in DMEM with 2% FBS. Plates were incubated for 24 h at 37 °C 5% CO2. To assess cell viability, 2 mL of 50% CellTiter Glo (Promega No. G7573) diluted in water was added to the cells, and luminescence was measured on an EnVision Plate Reader (PerkinElmer).

The results of both the primary antiviral assay and the host cell cytotoxicity counter screen were uploaded to Genedata Screener, Version 16.0. Data were normalized to neutral (DMSO) minus inhibitor controls (2.4 μM remdesivir for antiviral effects and 10 μM puromycin for infected host cell toxicity). For the uninfected host cell cytotoxicity counter screen, 40 μM puromycin (Sigma) was used as the positive control. Compounds were tested in technical triplicates, and inhibition curves were fitted with the four-parameter Hill Equation.

HCoV-229E/MRC5 and HCoV-OC43/Huh7 infectivity was measured using CPE assays performed by WuXi AppTec Co., Ltd. MRC5 and Huh7 cells were obtained from ATCC and AppTec, respectively. MRC5 cells were maintained in Minimum Essential Medium (Sigma) supplemented with 10% FBS (Excell), 1% l-glutamine (Gibco), 1% NEAA (Gibco), and 1% penicillin-streptomycin (Hyclone). Minimum Essential Medium supplemented with 5% FBS, 1% l-glutamine, 1% NEAA, and 1% penicillin-streptomycin was used as the assay medium.

Huh7 cells were maintained in Dulbecco’s modified eagle medium (Gibco) supplemented with 10% FBS, 1% l-glutamine, 1% NEAA, and 1% penicillin-streptomycin.

Cells were seeded in 96-well plates (20,00 MRC5 cells per well; 8,000 Huh7 cells per well) in 100 μL per well of assay medium and cultured at 37 °C and 5% CO_2_ overnight. The next day, the test compound was diluted with the assay medium and then added to the cells (50 μL per well). Then, 50 μL per well of assay medium diluted with virus was added. The final volume of the cell culture was 200 μL per well. The final concentrations of DMSO in the EC_50_ and CC_50_ test plates were 0.5% and 1%, respectively. The resulting cell culture was incubated at 35 °C for 3 days (229E) or 33 °C for 7 days (OC43) and 5% CO_2_ when virus infection in the virus control (cells infected with the virus, without compound treatment) displayed significant CPE. The CPE was measured by CellTiter-Glo following the manufacturer’s manual. The antiviral activity of compounds was calculated based on the protection of the virus-induced CPE at each concentration normalized by the virus control. The cytotoxicity of compounds was assessed under the same conditions in parallel but without virus infection. Cell viability was measured with CellTiter-Glo following the manufacturer’s manual.

The antiviral activity and cytotoxicity of compounds were expressed as % inhibition and % viability, respectively, and calculated using the formulas below:

Inhibition(%)=(CPD−VC)/(CC−VC)*100%

Viability(%)=(CPD−MC)/(CC−MC)*100%

CPD: values of the sample-treated wells.

VC: average value of virus control.

CC: average value of cell control (cells without virus infection or compound treatment).

MC: average value of medium control (medium only) wells.

EC_50_ and CC_50_ values were calculated using the GraphPad Prism software using the nonlinear regression model of log­(inhibitor) vs response, variable slope (four parameters).

#### Cellular Infectivity Assays for SARS-CoV-2 Omicron

SARS-CoV-2 omicron/Vero E6 infectivity was measured using the cytopathic effect (CPE) assay performed by Wuhan Institute of Virology, Chinese Academy of Sciences. SARS-CoV-2 omicron B.1.1.529 strain and Vero E6 cells were provided by the Wuhan Institute of Virology, Chinese Academy of Sciences.

Vero E6 cells were maintained in Dulbecco’s modified eagle medium (DMEM, Gibco) supplemented with 10% fetal bovine serum (FBS, Gibco) and 1% penicillin-streptomycin (Beyotime). DMEM supplemented with 2% FBS and 1% penicillin-streptomycin was used as the assay medium.

Vero E6 cells were seeded in 96-well plates at a density of 10,000 cells per well, in 100 μL per well of assay medium, and cultured at 37 °C and 5% CO_2_ overnight. The next day, test compounds were four-fold serially diluted with the assay medium and then added to the cells along with the Pgp inhibitor CP-100356. Then, cells were immediately infected with the SARS-CoV-2 B.1.1.529 strain at an MOI = 0.1. The final volume of the cell culture was 200 μL per well. The final concentrations of DMSO and CP-100356 in the cell culture medium were 0.5% and 2 μM, respectively. The resulting cell culture was incubated at 37 °C and 5% CO_2_ for 4 days when virus infection in the virus control (cells infected with the virus, without compound treatment) displayed significant CPE. The CPE was measured by CellTiter-Glo following the manufacturer’s manual. The antiviral activity of compounds was calculated based on the protection of the virus-induced CPE at each concentration normalized by the virus control.

The antiviral activity and cytotoxicity of compounds were assessed in the same manner as those for the wild-type.

### Virtual Screening (VS)

#### Compound Preparation for VS

The Takeda compound library comprising 1.5 M compounds was used for VS. To build three-dimensional (3D) conformers with all possible isomer and ionization states, LigPrep in the 2019–1 release of the Schrödinger modeling suite^35^ was used at 7.0 ± 2.0 pH units using the OPLS2005 force field.

#### Docking-Based VS

Four crystal structures of SARS-CoV-2 Mpro (PDB ID: 6LU7,^24^ 6M03,^36^ 6Y2G,^37^ and 5R7Y^38^) were used as docking templates. The protein structures were prepared using the Protein Preparation Wizard on Maestro of the 2019–1 release of the Schrödinger modeling suite with default settings. General Glide SP docking^39^ was conducted to identify the noncovalent inhibitors using the four SARS-CoV-2 Mpro proteins, and covalent docking was also conducted to identify covalent inhibitors with the protein of the crystal structure of SARS-CoV-2 Mpro (PDB ID: 6LU7). The docking score was normalized with the linear function by the heavy atom count (HAC) of the docked molecule to account for the correlation between the docking score and molecular size.^40^ When using the protein of 6LU7, GlideScore was normalized by GScore +0.057*HAC. When using the protein of 6Y2G, GlideScore was normalized by GScore +0.062*HAC. When using the protein of 5R7Y, GlideScore was normalized by GScore +0.035*HAC. In noncovalent docking, compounds with GlideScore ≤ −6.5 were selected. In covalent docking, the compounds with the score ≤ −4.5 were selected. Physicochemical property filtering was conducted by using the following criteria: 200 ≤ molecular weight ≤ 600, −2 ≤ AlogP ≤ 5 using the Pipeline Pilot version 2017.^41^

#### Ligand-Based VS

To identify noncovalent inhibitors, multiple similarity search ways with the known SARS-CoV-1 and SARS-CoV-2 Mpro inhibitors as of early 2020 ^42–46^ were conducted by using 1) ECFP-4 (extended connectivity fingerprints),^47^ 2) chemically advanced template search (CATS) descriptor based on topological pharmacophore models,^48–50^ and 3) MCS-based search with atom/bond/mismatches. The similarity search using ECFP-4 was calculated in the Pipeline Pilot with the cutoff value of the Tanimoto coefficient (Tc, Tc > 0.7). CATS with the cutoff value of the distance (CATS distance <10) was calculated by the CopyCATS tool, and an MCS-based search was conducted in the internal tool. To identify covalent inhibitors, a substructure search with the group that covalently binds to cysteine residues was conducted. The query compounds used in the similarity/substructure search are listed in the Supporting Information. Physicochemical property filtering was conducted in the same manner as that of the docking-based VS.

#### FEP+ Calculation

The protein structures for simulations were prepared using the Protein Preparation Wizard on Maestro with default settings. All of the FEP+ simulations were carried out using the 2021–4 release of the Schrödinger modeling suite with default settings. For evaluating the introduction of P3 substituents on the piperidine core, the crystal structure of SARS-CoV-2 Mpro in complex with compound **4** was used, and the P3 substituents on the piperidine core were manually drawn in Maestro as initial structures for the FEP+ calculation. For the exploration of phenyl substituents to optimize S2 pocket interaction, the crystal structure of SARS-CoV-2 Mpro in complex with compound **12** was used, and the P2 substituents on the phenyl ring were manually drawn on Maestro as initial structures. For FEP+ calculations, compounds **27** and **28** were run manually, and 7,330 compounds were run automatically in LiveDesign (LiveDesign Version 8.11.1. New York, NY: Schrödinger, Inc.).^35^ The crystal structure of SARS-CoV-2 Mpro in complex with compound **12** was used as the reference. Input poses were generated using core constrained docking with Glide SP. For these calculations, default settings were employed other than increasing the number of windows to 24 and including the P3 substituent beyond the P3 amide bond in the hot region to enhance sampling. All calculations were run for 5 ns, except for follow up calculations for **27** and **28** that were extended to 15 ns.

#### Log *D*_pH 7.4_ (Log D) Studies

Determination Log D, which is a partition coefficient between 1-octanol and aqueous buffer pH 7.4, of the compounds was measured using a chromatographic method, as previously described.^51^

#### Thermodynamic Solubility Measurement by the Shake-Flask Method

To measure the thermodynamic solubility, 0.4 mg of the drug substance was weighed into the Whatman Mini-UniPrepTM Syringeless Filter Device containing a 0.45 μM polytetrafluoroethylene filter membrane. Four hundred microliters of JP1 (pH 1.2) or JP2 (pH 6.8) were added to the vials. The vials were incubated at room temperature with shaking at 800 rpm for 16 h and centrifuged at 25 °C for 20 min at 4000 rpm. The centrifuged samples were filtrated by compressing the vials and diluted if needed. The drug concentration of the filtrates was determined by HPLC. HPLC analysis was conducted with an ACQUITY UPLC BEH C18 column (Waters Corp., Massachusetts, U.S.A).

### Animal Experiments

All animal experiments were performed following the protocols and approved by the Institutional Animal Care and Use Committee in a contractor (WuXi AppTec, Ethics Approval Number: SZ20220609-Mice).

#### Mouse PK Studies

A single bolus intravenous (IV) dose of compound **30** (1.0 mg/kg nominal in 20% hp-β-cyclodextrin in 0.05 M methanesulfonic acid, pH 3) was administered by tail vein injection (1 mL/kg) to female C57BL/6J mice (n = 3). Compound **30** was also administered orally (PO) via gavage (30 mg/kg nominal as a suspension in 0.5% Tween and 0.5% methylcellulose) to female C57BL/6J mice. In the IV group, blood samples were collected serially at 0.083, 0.25, 0.5, 1, 2, 4, and 7 h post dose. In the PO group, lung tissues and blood samples were collected terminally at 0.5, 1, 3, 7, and 24 h post PO dose (n = 3 per time point). Plasma and lung concentrations were accessed by LC-MS/MS.

#### Plasma Protein Binding

Plasma protein binding (PPB) was obtained by using equilibrium dialysis. Samples were analyzed by LC-MS/MS after a 6-h incubation. Fraction unbound (fu) was calculated using the following equation: fu = F/T, where F is the free compound concentration as determined by the calculated concentration on the buffer side, and T is the total compound concentration as determined by the calculated concentration on the matrix side. Average fu was reported based on three replicates.

#### Metabolic Stability Studies

In vitro oxidative metabolic studies of the test compounds were conducted using human and mouse hepatic microsomes. The hepatic microsome, NADPH-generating system, and compounds (1 μM) were mixed and incubated at 37 °C for 15 and 30 min. The remaining compound concentration was assessed by LC-MS/MS. Metabolic clearance was determined by the disappearance of the parent compound from the reaction mixtures. All incubations were carried out in duplicate.

#### PAMPA Permeability Studies

Permeability across the artificial membrane was assessed using a 96-well filter plate: STIRWELLTM PAMPA Sandwich. After addition of test compounds at 10 μM to the donor side, it was incubated for 3 h at room temperature. The compound signal was determined by LC-MS/MS, and the apparent permeability (Papp) was calculated.

#### MDCK-MDR1 Permeability Studies

The transcellular transport study was performed using cultured human MDR1-expressing MDCK cells. Transcellular transport was initiated by the addition of 1 μM test compounds either to the apical or the basolateral side and incubation for 60 min. Compound concentration on each side was determined by LC-MS/MS, followed by the calculation of the permeability rate, Papp, and efflux ratio (ER).

#### CYP Inhibition Studies

CYP inhibition potency of compound **30** was evaluated using a human hepatic microsome with CYP isoform specific substrates (CYP1A2:5 μM tacrine, CYP2C8:5 μM paclitaxel, CYP2C9:300 μM tolbutamide, CYP2D6:4 μM dextromethorphan, CYP3A4:2 μM midazolam). The hepatic microsome, NADPH-generating system, substrates, and compounds (10 μM) were mixed and incubated at 37 °C for 10 min. Percent (%) inhibition was calculated based on the remaining CYP activity compared to the mixture without an inhibitor.

#### Crystallization of SARS-CoV-2 Mpro with Compounds 4, 12, and 29

Crystals were grown at 20 °C by equilibrating the protein solution against 0.1 M bis-tris pH 6.5, 32% (w/v) polyethylene glycol 2000 monomethyl ether, by the hanging drop vapor diffusion method. Apo crystals were soaked with 5 to 20 mM ligand in mother liquor for up to 1 h. For cryoprotection, crystals were transferred to the mother liquor supplemented with 25% ethylene glycol.

Diffraction data for the complexes with compounds **4** and **12** were collected at the Advanced Light Source (ALS). Images were processed using XDS^52^ for integration and AIMLESS^53^ for scaling. The initial model was obtained by molecular replacement with the program PHASER^54^ using PDB entry 6Y2E as the search model. After an initial round of refinement with REFMAC,^55^ refinement of the structure was completed using the program PrimeX in the Maestro software suite.^56^ The coordinates and structure factors have been deposited in the Protein Data Bank with accession codes 9DDG (compound **4**) and 9DDF (compound **12**).

Diffraction data for the complex with compound **29** were collected from single cryogenically protected crystals at the Swiss Light Source (SLS). Images were processed using autoPROC,^57^ which involved the use of the additional software XDS for integration and AIMLESS for scaling. The initial model was obtained by molecular replacement with the program PHASER using PDB entry 6Y2E as the search model. Several cycles of model building with COOT^58^ and refinement with REFMAC were performed for improving model quality. The coordinates and structure factors have been deposited in the Protein Data Bank with accession code 9NU6 (compound **29**). The data collection and structure refinement statistics are summarized in Table S5.

## Supplementary Material















## Notes

In vitro enzyme activities for compound **4** and compound **28**, selectivity against human proteases for compound **28**, conformational flexibility of Gln189, overlay of crystal structures of SARS-CoV-2, MERS, 229E, and NL63 Mpro, in vitro safety profile for compound **30**, protein crystal structure determination statistical summary, compound synthesis schemes for compounds **3**-**29**, analytical HPLC and NMR spectra for compound **3**-**30**, and experimental procedures for human proteases and in vitro safety (PDF)
Crystal structure for compound **4** (PDB)
Crystal structure for compound **12** (PDB)
Crystal structure for compound **29** (PDB)
Molecular formula strings (CSV)
Query compounds used in similarity/substructure search (CSV)
Experimental Δ*G* and FEP+ Δ*G* of 85 synthesized compounds for FEP+ performance (CSV)

## Notes

PDB code for SARS-CoV-2 Mpro in complex with compound **4** is 9DDG. PDB code for SARS-CoV-2 Mpro in complex with compound **12** is 9DDF. PDB code for SARS-CoV-2 Mpro in complex with compound **29** is 9NU6. Authors will release the atomic coordinates upon article publication.

## Abbreviations

ACN: acetonitrile
CATS: chemically advanced template search
cLogP: calculated n-octanol/water partition coefficient
COVID: coronavirus disease
CPE: cytopathic effect
DAPI: 4′,6-diamidino-2-phenylindole
DCM: dichloromethane
DMEM: Dulbecco’s modified eagle medium
ER: efflux ratio
EtOA: ethyl acetate
EtOH: ethanol
FBS: fetal bovine serum
FEP: free energy perturbation
HAC: heavy atom count
HPLC: high-performance liquid chromatography
LC-MS: liquid chromatography mass spectrometry
MD: molecular dynamics
NEAA: nonessential amino acids
NMR: nuclear magnetic resonance
PAMPA: parallel artificial membrane permeability assay
PBS: phosphate buffered saline
PE: petroleum ether
PK: pharmacokinetics
PMSF: phenylmethylsulfonyl fluoride
PPB: plasma protein binding
SEC: size exclusion chromatography
SLS: Swiss Light Source
Tris: tris­(hydroxymethyl)­aminomethane
VS: virtual screening
