# Supplementary material for: Discovery of Highly Potent Noncovalent Inhibitors of SARS-CoV‑2 Main Protease through Computer-Aided Drug Design
Source: J Med Chem. 2025 Oct 12;68(20):21330–45. doi: 10.1021/acs.jmedchem.5c01199 (PMC12557384; doi:10.1021/acs.jmedchem.5c01199)
Supplement: Supplementary file 1 [file jm5c01199_si_001.pdf]

Supporting information

Discovery of highly potent noncovalent inhibitors of SARS-CoV-2 main protease through computer-aided drug design

Atsutoshi Okabe[a*], Daniel W. Carney[b], Michiko Tawada[a], Thamina Akther[a], Jumpei Aida[a], Terufumi Takagi[a], Douglas R. Dougan[b], Abba E. Leffler[c], Jeffrey A. Bell[c], Leah Frye[d], Eugene R. Hickey[c], Mallareddy Komandla[b], Will Tao[b], Jangir Selimkhanov[b], Kazuko Yonemori[a], Edcon Chang[b], Kumar Saikatendu[b], and Atsuko Ochida[a*]

[a] Takeda Pharmaceutical Company Limited, 26-1 Muraoka-Higashi 2-chrome, Fujisawa, Kanagawa 251-8555, Japan

[b] Takeda Development Center Americas, Inc., 9625 Towne Centre Drive, San Diego, CA 92121, United States

[c] Schrödinger, Inc., 1540 Broadway, New York, New York 10036, United States

[d] Schrödinger, Inc., 101 SW Main Street, Suite 1300, Portland, Oregon 97204, United States

*Corresponding author, E-mail: atsutoshi.okabe@takeda.com (Atsutoshi Okabe), atsuko.ochida@takeda.com (Atsuko Ochida)

Contents

Table S1. SAR analysis on nitrophenyl moiety of compound **4**……………………………………………..S3

Table S2. *In vitro* enzyme activities against human coronavirus Mpro of compound **28**…………………………………………………………………………………………….…S4

Table S3. Selectivity of compound **28** against human proteases……………………………………….S5

Figure S1. Conformational flexibility of Gln189 represented through B-factor and FEP+ simulation…..S6

Figure S2. Overlay of crystal structures of SARS-CoV-2 Mpro, MERS Mpro, 229E Mpro and NL63 Mpro…………………………………………………………………………………………...S7

Table S4. *In vitro* safety profile of compound **30**……..…………………………………….……….S8

Table S5. Protein crystal structure determination statistical summary……………………………………S9

Experimental procedures for the synthesis of compounds **3-29**……………………………………S10-S34

Experimental procedures for analytical HPLC…………………………………………………….S35

1 H-NMR spectra for compound **3-29**…………………………………………………………...S36-S52

1 H-NMR and 13C-NMR spectra for compound **30**…………………………………………………S53

Analytical HPLC of compound **30**………………………………………………………………….S54-S55

Experimental procedures for human protease panel…………………………………………......S56-S60
Experimental procedures for *in vitro* safety…………………………………………………..S61-S62

Table S1. SAR analysis on nitrophenyl moiety of compound **4**

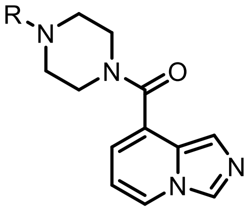

	R	SARS-CoV-2 Mpro IC_{50} (nM)
4	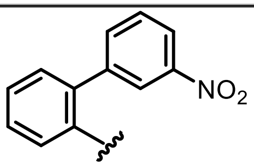	840
5	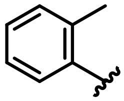	36,000
6	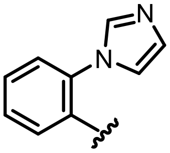	4,500
7	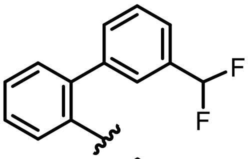	3,000
8	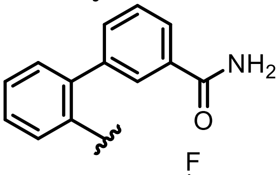	1,400
9	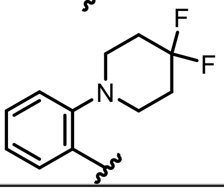	1,400

Table S2. *In vitro* enzyme activities against human coronavirus Mpro of compound **28**

	Enzyme IC_{50} (nM)
SARS-CoV-2 Mpro	<12
SARS-CoV-1 Mpro	13
229E Mpro	29
HKU1 Mpro	16
MERS Mpro	61
NL63 Mpro	49
OC43 Mpro	47

Table S3. Selectivity of compound **28** against human proteases

Protease	IC_{50} (μM)
Chymotrypsin C	>100
Human Caspase 2	>100
Human Cathepsin B	>100
Human Cathepsin D	>100
Human Cathepsin K	>100
Human Cathepsin L	>100
Human Cathepsin S	>100
Human Elastase	>100
Human Pepsin	>100
Human Thrombin	>100

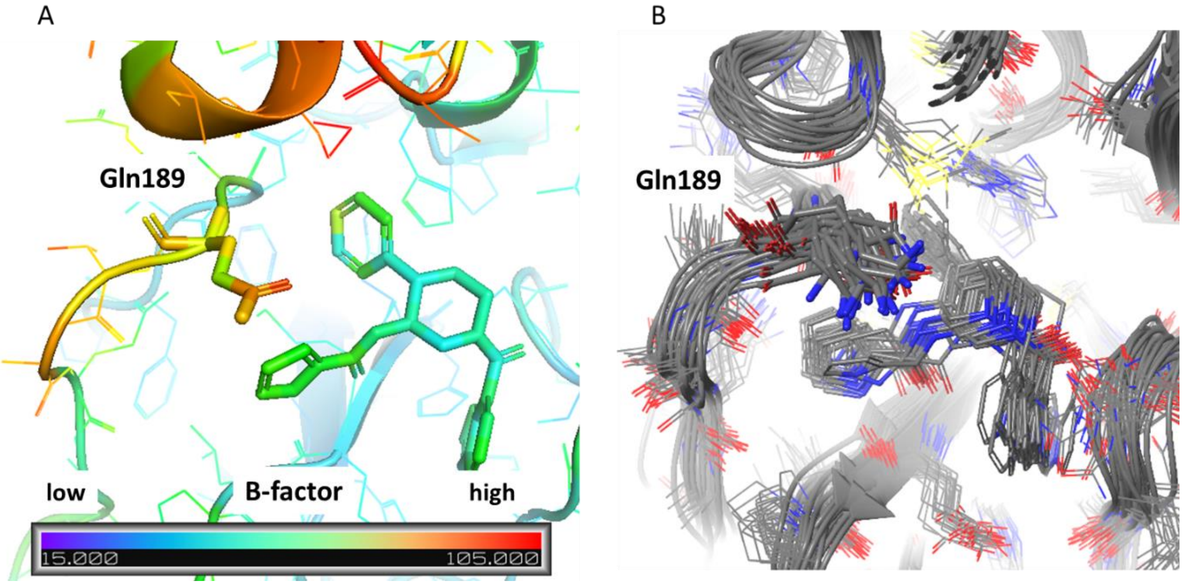

Figure S1. Conformational flexibility of Gln189 represented through (A) B-factor in the crystal structure of SARS-CoV-2 Mpro in complex with compound **12**, (B) overlay of snapshots in FEP+ simulation for compound **12**. Blue and green B-factor coloration suggest regions of little movement, while red and orange colors suggest regions of greater movement in the crystal structure.

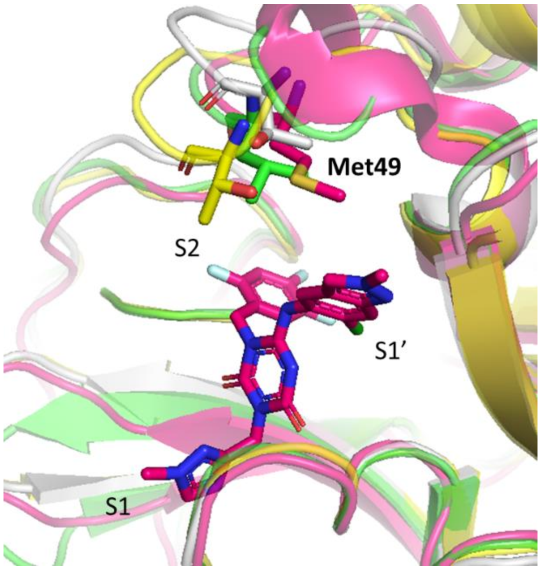

Figure S2. Overlay of crystal structures of SARS-CoV-2 Mpro (PDB ID: 7VU6, magenta), MERS Mpro (PDB ID: 4RSP, green), 229E Mpro (PDB ID: 2ZU2, white) and NL63 Mpro (PDB ID: 5GWY, yellow). Only ensitrelvir in 7VU6 is shown among ligand structures in the crystal structures.

Table S4. *In vitro* safety profile of compound **30**

In vitro safety assays	Results
hERG inhibition assay	IC_{50} > 30 μM
Nav1.5 inhibition assay	IC_{50} > 30 μM
hLiMT (human liver microtissue) test	IC_{50} > 100 μM
In vitro micronucleus test	Negative

Table S5. Protein crystal structure determination statistical summary

Compound	**4**	**12**	**29**
**Data Collection**			
Space group	C 1 2 1	C 1 2 1	P 2_1 2_1 2_1
a,b,c (Å)	97.94, 81.79, 51.74	97.11, 81.94, 54.83	100.60, 103.70, 67.68
β (°)	114.60	117.11	90.00
Resolution range (Å)	44.52-1.40	48.81-1.55	56.68 -1.965
Unique observations	74117	54559	43985
Completeness	99.6	98.4	86.0
Multiplicity	3.7	4.2	7.4
R_{merge}	0.048	0.038	0.108
$\langle I/\sigma(I)\rangle$	10.3	15.373	10.479
**Refinement**			
Copies in ASU	1	1	2
R_{work}, R_{free}	0.207, 0.220	0.225, 0.251	0.173, 0.218
Number of atoms	5374	5107	4986
Mean B factor (Å^2)	28.44	36.19	36.59
RMSD (bonds) (Å)	0.0118	0.0116	0.0064
RMSD (angles) (°)	1.746	1.746	1.733
**PDB ID**	9DDG	9DDF	9NU6

Experimental procedures for synthesis of compound 3-29

***Tert*-butyl 4-(3'-nitro-[1,1'-biphenyl]-2-yl)piperazine-1-carboxylate**

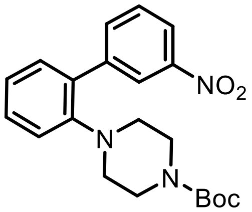

A mixture of *tert*-butyl 4-(2-bromophenyl)piperazine-1-carboxylate (500 mg, 1.5 mmol), $Pd(Ph_3P)_4$ (169 mg, 0.15 mmol), (3-nitrophenyl)boronic acid (489 mg, 2.9 mmol), 2 N aqueous Na_2CO_3 (2.2 ml, 2.2 mmol) in DME (18 ml) and water (2 ml) was stirred at 100 °C under MW irradiation for 1 h. The reaction mixture was filtered and concentrated to a crude residue. The product was purified silica gel flash chromatography (20% EtOAc in hexane). The title compound (195 mg, 35% yield) was obtained as an orange oil. ESI-MS $[M+H-100]^+$ calcd. for $C_{21}H_{25}N_3O_4$: 284.1, found: 284.1.

(4-(3'-nitro-[1,1'-biphenyl]-2-yl)piperazin-1-yl)(pyridin-3-yl)methanone (3)

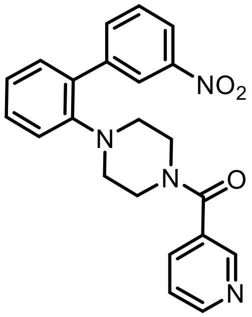

To a solution of *tert*-butyl 4-(3'-nitro-[1,1'-biphenyl]-2-yl)piperazine-1-carboxylate (158 mg, 0.56 mmol) in THF (2 ml) and MeOH (1 ml) was added HCl (4 N in CPME) (2 ml, 0.37 mmol) at 0°C. The reaction mixture was stirred at room temperature for 18 h and then was concentrated to provide the crude deprotected intermediate, which was used in the next step without purification.

To a mixture of nicotinic acid (69 mg, 0.56 mmol), HATU (321 mg, 0.84 mmol), and the crude deprotected intermediate (0.56 mmol) in DMF (2.0 ml, 25.8 mmol) was added DIPEA (0.197 ml, 1.13 mmol). The reaction mixture was stirred at room temperature for 2 h, and then was diluted with water and filtered. The product was purified from the filtered solid by prep-HPLC (column: YMC-Actus Triant C18 50 x 20 mm x 5 µm) mobile phase: [water (NH_4HCO_3)-ACN]; B%: 40%-60%, 7 min). The title compound (43 mg, 0.11 mmol, 20% yield over 2 steps) was obtained as a yellow solid. ^1H NMR (300 MHz, $CDCl_3$) δ 2.70-2.86 (2H, m), 2.95 (2H, br s), 3.27-3.41 (2H, m), 3.60-3.81 (2H, m), 7.11 (1H, dd, J = 8.0, 1.0 Hz), 7.16-7.23 (1H, m), 7.30 (1H, d, J = 1.7 Hz), 7.33-7.37 (1H, m), 7.38-7.42 (1H, m), 7.59 (1H, t, J = 7.9 Hz), 7.72 (1H, dt, J = 7.8, 2.0 Hz), 7.94 (1H, dt, J = 7.7, 1.4 Hz), 8.14-8.19 (1H, m), 8.62-8.68 (3H, m). ESI-MS $[M+H]^+$ calcd. for $C_{22}H_{20}N_4O_3$: 389.2, found: 389.2.

imidazo[1,5-a]pyridin-8-yl(4-(3'-nitro-[1,1'-biphenyl]-2-yl)piperazin-1-yl)methanone (4)

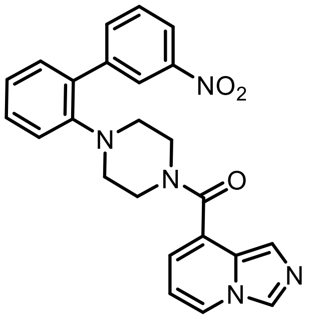

To a solution of tert-butyl 4-(3'-nitro-[1,1'-biphenyl]-2-yl)piperazine-1-carboxylate (158 mg, 0.31 mmol) in THF (2 ml) and MeOH (1 ml) was added HCl (4N in CPME) (2 ml, 0.37 mmol) at 0°C. The reaction mixture was stirred at room temperature for 18 h and then was concentrated to provide the crude deprotected intermediate, which was used in the next step without purification.
To a mixture of imidazo[1,5-a]pyridine-8-carboxylic acid (50.7 mg, 0.31 mmol), HATU (178 mg, 0.47 mmol) and the crude deprotected intermediate (0.31 mmol) in DMF (2.0 ml) was added DIPEA (0.11 ml, 0.63 mmol). The reaction mixture was stirred at room temperature for 2 h and then was diluted with water and filtered. The product was purified from the filtered solid by prep-HPLC (column: YMC-Actus Triant C18 50*20 mm*5 µm) mobile phase: [water (NH_4HCO_3)-ACN]; B%: 40%-60%, 7 min). The title compound (7.8 mg, 5.8 % yield over 2 steps) was obtained as an off white solid. ^{1}H NMR (300 MHz, $CDCl_3$) δ 2.70-3.02 (4H, m), 3.17-3.37 (2H, m), 3.61-3.87 (2H, m), 6.54-6.64 (1H, m), 6.75 (1H, d, J = 6.5 Hz), 7.11 (1H, d, J = 8.0 Hz), 7.20 (1H, d, J = 7.4 Hz), 7.29 (1H, d, J = 1.5 Hz), 7.36-7.46 (2H, m), 7.54-7.64 (1H, m), 7.91-7.96 (2H, m), 8.11-8.21 (2H, m), 8.62 (1H, s). ESI-MS $[M+H]^+$ calcd. for $C_{19}H_{20}N_4O$: 328.2, found: 428.1.

imidazo[1,5-a]pyridin-8-yl-[4-(o-tolyl)piperazin-1-yl]methanone (5)

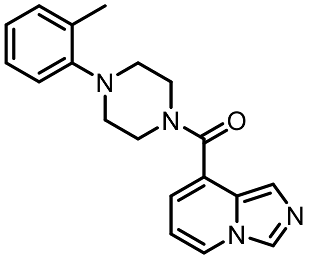

To a solution of 1-(*O*-tolyl)piperazine (50 mg, 0.28 mmol) and imidazo[1,5-a]pyridine-8-carboxylic acid (46 mg, 0.28 mmol) in DMF (2 mL) was added PyBOP (221 mg, 0.43 mmol) and DIEA (147 mg, 1.13 mmol). The mixture was stirred at room temperature for 2 h. The mixture was diluted with EtOAc (100 mL) and washed with brine (50 mL x 3). The organic layer was dried over Na_2SO_4, and concentrated to give crude product. The residue was purified by prep-HPLC (column: Phenomenex C18 75 x 30mm x 3um; mobile phase: [water (NH_4HCO_3)-ACN]; B%: 26%-56%, 10min). The title compound was obtained as a yellow solid (32 mg, 35% yield). ^{1}H NMR (MeOD, 400 MHz) δ_H = 8.43 (s, 1H), 8.33 (d, *J* = 7.2 Hz, 1H), 7.43 (s, 1H), 7.18-7.14 (m, 2H), 7.07 (s, 1H), 7.01-6.91 (m, 2H), 6.77 (s, 1H), 4.09-3.87 (m, 2H), 3.63-3.52 (m, 2H), 3.09-2.80 (m, 4H), 2.32 (s, 3H). ESI-MS $[M+H]^+$ calcd. for $C_{24}H_{21}N_5O_3$: 321.2, found: 321.2.

***tert*-butyl 4-(2-(1*H*-imidazol-1-yl)phenyl)piperazine-1-carboxylate**

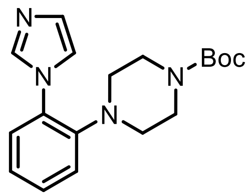

A mixture of *tert*-butyl 4-(2-bromophenyl)piperazine-1-carboxylate (500 mg, 1.47 mmol), imidazole (300 mg, 4.40 mmol), K_2CO_3 (405 mg, 2.93 mmol), (1*S*,2*S*)-(+)-1,2-diaminocyclohexane (51 mg, 0.439 mmol) and CuI (84 mg, 0.44 mmol) were taken up into a microwave tube in NMP (5 mL). The sealed tube was heated at 150 °C for 4 h under microwave irradiation. The reaction mixture was diluted with EtOAc (50 mL) and washed with water (20 mL x3). The organic phase was dried over anhydrous Na_2SO_4, filtered, and concentrated. The product was purified by silica gel flash chromatography (52% EtOAc in PE). The title compound (180 mg, 35.6% yield) was obtained as a yellow oil. ESI-MS $[M+H]^+$ calcd. for $C_{18}H_{24}N_4O_2$: 329.2, found: 329.2.

(4-(2-(1*H*-imidazol-1-yl)phenyl)piperazin-1-yl)(imidazo[1,5-a]pyridin-8-yl)methanone (6)

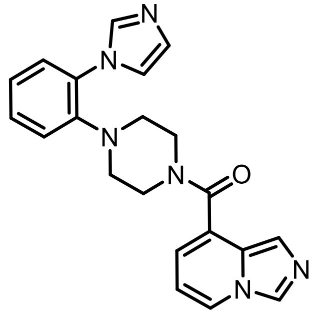

A mixture of *tert*-butyl 4-(2-(1*H*-imidazol-1-yl)phenyl)piperazine-1-carboxylate (180 mg, 0.55 mmol) in HCl/dioxane (2 mL) was stirred at room temperature for 10 h. The reaction mixture was concentrated to give the crude deprotected intermediate as a white solid, which was used without purification.

To a mixture of the crude deprotected intermediate (50 mg, 0.22 mmol) and imidazo[1,5-a]pyridine-8-carboxylic acid (36 mg, 0.22 mmol) in DMF (2 mL) was added PyBOP (171 mg, 0.33 mmol) and DIEA (142 mg, 1.10 mmol). The reaction was stirred at room temperature for 10 h. The reaction mixture was diluted with DCM (20 mL) and washed with water (10 mL x 3). The organic phase was dried over anhydrous Na_2SO_4, filtered and concentrated to give crude product. The crude product was purified by prep-HPLC (column: Phenomenex C18 75 x 30mm x 3um; mobile phase: [water (NH_4HCO_3)-ACN]; B%:12%-42%, 10min). The title compound (1.6 mg, 1.8% yield, 94.9% purity) was obtained as a yellow solid. ^1H NMR (DMSO-d_6, 400MHz) δ_H = 8.42 (s, 1H), 8.32 (d, J = 8.0 Hz, 1H), 8.06 (s, 1H), 7.51 (s, 1H), 7.42-7.35 (m, 3H), 7.35-7.24 (m, 3H), 6.92-6.89 (m, 1H), 6.78-6.74 (m, 1H), 3.81-3.74 (m, 2H), 3.51-3.38 (m, 2H), 2.88-2.75 (m, 4H). ESI-MS $[M+H]^+$ calcd. For $C_{21}H_{20}N_6O$: 373.2, found: 373.3.

4-(2-bromophenyl)piperazin-1-yl]-imidazo[1,5-a]pyridin-8-yl-methanone

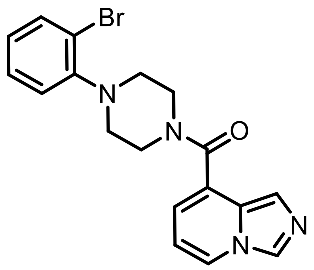

To a solution of *tert*-butyl 4-(2-bromophenyl)piperazine-1-carboxylate (700 mg, 2.05 mmol) in DCM (2 mL) was added HCl/dioxane (4 M, 10 mL). The reaction mixture was stirred at room temperature for 2 h. The reaction mixture was concentrated to give crude deprotected intermediate (500 mg, crude) as a brown oil, which was used without purification. To a solution of the crude deprotected intermediate (250 mg, 1.04 mmol) and imidazo[1,5-a]pyridine-8-carboxylic acid (168 mg, 1.04 mmol) in pyridine (5 mL) was added EDCI (398 mg, 2.07 mmol). The mixture was stirred at 25°C for 12 h. Water (30 mL) was added to the reaction mixture and the mixture was extracted with DCM (50 mL x3). The combined organic extracts were washed with saturated aqueous NH_4Cl (100 mL), dried over anhydrous Na_2SO_4, and concentrated to give crude product. The crude product was purified by silica gel flash chromatography (0-100% EtOAc in PE). The title compound (154 mg, 23% yield) was obtained as a yellow solid. ESI-MS $[M+H]^+$ calcd. for $C_{18}H_{17}BrN_4O$: 386.3, found: 387.0.

(4-(3'-(difluoromethyl)-[1,1'-biphenyl]-2-yl)piperazin-1-yl)(imidazo[1,5-a]pyridin-8-yl)methanone (7)

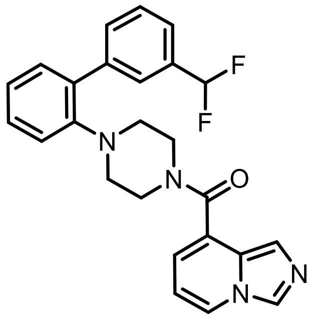

To a mixture of 4-(2-bromophenyl)piperazin-1-yl]-imidazo[1,5-a]pyridin-8-yl-methanone (50 mg, 0.13 mmol) and 3-(difluoromethyl)phenyl]boronic acid (67 mg, 0.39 mmol) in dioxane (4 mL) and water (1 mL) was added Pd(dppf)Cl_2 (10 mg, 0.01 mmol) and K_2CO_3 (36 mg, 0.26 mmol). The mixture was degassed and purged with N_2 and then the mixture was stirred at 80 °C for 2 h under N_2 atmosphere. The reaction was washed with water (50 mL) and the resulting mixture was extracted with EtOAc (50 mL x 3). The combined organic extracts were dried over anhydrous Na_2SO_4, filtered and concentrated to give crude product. The crude product was purified by prep-HPLC (column: Phenomenex C18 75 x 30 mm x 3 um; mobile phase: [water (NH_3H_2O+ NH_4HCO_3)-ACN]; B%: 35%-65%, 10min). The title compound (3.6 mg, 6.3% yield) was obtained as a yellow solid. ^{1}H NMR (MeOD, 400 MHz) δ_H =8.39 (s, 1H), 8.30 (d, *J* = 6.8 Hz, 1H), 7.87 (s, 1H), 7.74 (d, *J* = 8.0 Hz, 1H), 7.57-7.46 (m, 2H), 7.36-7.24 (m, 3H), 7.17-7.11 (m, 2H), 6.85-6.65 (m, 3H), 4.56 (s, 2H), 3.68 (s, 2H), 3.00-2.70 (m, 4H). ESI-MS $[M+H]^+$ calcd. for $C_{25}H_{22}F_2N_4O$: 433.5, found: 433.2.

***tert*-butyl 4-(3'-(methoxycarbonyl)-[1,1'-biphenyl]-2-yl)piperazine-1-carboxylate**

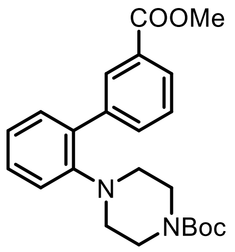

To a solution of *tert*-butyl 4-(2-bromophenyl)piperazine-1-carboxylate (550 mg, 1.61 mmol) and (3-methoxycarbonylphenyl)boronic acid (290 mg, 1.61 mmol) in dioxane (6 mL) and water (1.5 mL) was added Pd(dppf)Cl_2 (118 mg, 0.16 mmol) and K_2CO_3 (668 mg, 4.84 mmol). The mixture was degassed and purged with N_2, and then the mixture was stirred at 100 °C for 12 h under N_2 atmosphere. The reaction mixture was filtered via a celite pad and the filter cake was washed with DCM (100 mL). The resulting filtrate was concentrated, taken up in water (50 mL), and extracted with DCM (50 mL x 3). The combined organic extracts were dried over anhydrous Na_2SO_4 and concentrated. The crude product was purified by silica gel flash chromatography (4% EtOAc in PE). The title compound (500 mg, 75% yield) was obtained as a yellow oil. ESI-MS $[M+H]^+$ calcd. for $C_{23}H_{28}N_2O_4$: 397.2, found: 397.3.

***tert*-butyl 4-(3'-carbamoyl-[1,1'-biphenyl]-2-yl)piperazine-1-carboxylate**

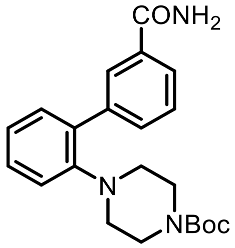

To a mixture of *tert*-butyl 4-(3'-(methoxycarbonyl)-[1,1'-biphenyl]-2-yl)piperazine-1-carboxylate (500 mg, 1.26 mmol) in THF (2.5 mL) and water (2.5 mL) was added LiOH.H_2O (159 mg, 3.78 mmol). The mixture was stirred at room temperature for 12 h. The mixture was diluted with water (20 mL) and washed with PE (20 mL). The pH of the aqueous phase was adjusted to around 3 with 2 M HCl. The aqueous phase was diluted with water (20 mL) and extracted with DCM (30 mL x3). The combined organic extracts were dried over Na_2SO_4 and concentrated to give crude saponified intermediate as a brown solid (400 mg, crude), which was used without purification.

To a solution of the crude saponified intermediate (200 mg, 0.52 mmol) in DMF (2 mL) was added NH_4Cl (140 mg, 2.61 mmol), DIEA (203 mg, 1.57 mmol) and HATU (298 mg, 0.78 mmol). The mixture was degassed and purged with N_2 for 3 times. The mixture was stirred at 25 °C for 2 hr. Water (20 mL) was added to the reaction mixture and the mixture was extracted with EtOAc (20 mL x3). The combined organic extracts were washed with brine (30 mL x3), dried over anhydrous Na_2SO_4 and concentrated to give crude product. The crude product was purified by silica gel flash chromatography (29% EtOAc in PE). The title compound obtained as a colorless oil (190 mg, 93% yield). ESI-MS $[M+H]^+$ calcd. for $C_{22}H_{27}N_3O_3$: 382.2, found 382.2.

2'-(4-(imidazo[1,5-a]pyridine-8-carbonyl)piperazin-1-yl)-[1,1'-biphenyl]-3-carboxamide (8)

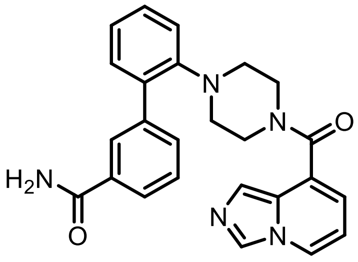

To a mixture of *tert*-butyl 4-(3'-carbamoyl-[1,1'-biphenyl]-2-yl)piperazine-1-carboxylate (190 mg, 0.50 mmol) in DCM (2 mL) was added HCl/dioxane (4 M, 2 mL). The mixture was stirred at room temperature for 1 h. The reaction mixture was concentrated directly to give crude deprotected intermediate (140 mg, crude), which was used without purification.

To a solution of the crude deprotected intermediate (50 mg, 0.18 mmol) and imidazo[1,5-a]pyridine-8-carboxylic acid (29 mg, 0.18 mmol) in DMF (1 mL) was added PyBOP (139 mg, 0.27 mmol) and DIEA (115 mg, 0.89 mmol). The mixture was stirred at room temperature for 1 h. Water (30 mL) was added and the resulting mixture was extracted with DCM (30 mL x 3). The combined organic extracts were washed with brine (50 mL x 3), dried over anhydrous Na_2SO_4 and concentrated to give crude product. The product was purified by prep-HPLC (column: C18-6 100 x 30 mm x 5 um; mobile phase: [water (FA)-ACN]; B%: 23%-43%, 15 min). The title compound (19.6 mg, 24% yield, 94.4% purity) was obtained as a white solid.^{1}H NMR (DMSO-d_6, 400 MHz) δ_H =9.28 (s, 1H), 8.51 (d, J = 7.2 Hz, 1H), 8.16-8.13 (m, 1H), 8.00 (m, 1H), 7.88 (s, 1H), 7.84-7.79 (m, 2H), 7.49 (t, J = 7.6 Hz, 1H), 7.37-7.27 (m, 3H), 7.17-7.09 (m, 3H), 7.01 (t, J = 7.2 Hz, 1H), 3.34-3.15 (m, 4H), 2.91-2.67 (m, 4H). ESI-MS $[M+H]^+$ calcd. for $C_{25}H_{23}N_5O_2$: 426.2, found: 426.1.

4,4-difluoro-1-(2-nitrophenyl)piperidine

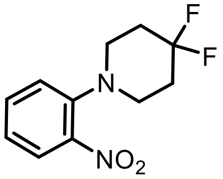

To a solution of 1-fluoro-2-nitrobenzene (256 µl, 3.54 mmol) and K_2CO_3 (490 mg, 3.54 mmol) in CH_3CN (145 mg, 3.54 mmol) was added 4,4-difluoropiperidine (747 µl, 5.32 mmol). The reaction mixture was heated under reflux for 3 hours. Then the reaction was cooled to room temperature and quenched by the addition of water (40 mL). The resulting mixture was extracted with EtOAc. The combined organic extracts were washed with brine (2 × 50 ml). The organic was dried over Na2SO4 and concentrated to give 4,4-difluoro-1-(2-nitrophenyl)piperidine (391 mg, 1.61 mmol, 46% yield) as an oil, which was used without further purification. ^{1}H NMR (300 MHz, $CDCl_3$) δ 2.16 (4H, tt, J = 13.8, 5.8 Hz), 3.12-3.22 (4H, m), 7.04-7.22 (2H, m), 7.51 (1H, ddd, J = 8.3, 7.2, 1.6 Hz), 7.78-7.85 (1H, m). ESI-MS $[M+H]^+$ calcd. for $C_{11}H_{12}F_2N_2O_2$: 243.1, found: 243.0.

2-(4,4-difluoropiperidin-1-yl)aniline

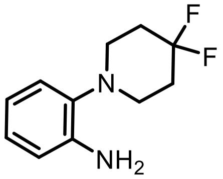

To a solution of 4,4-difluoro-1-(2-nitrophenyl)piperidine (600 mg, 2.48 mmol) and in MeOH (15 ml, 371 mmol) was added 10% Pd-C (791 mg, 0.74 mmol) and the reaction mixture was purged with hydrogen

gas from a balloon and stirred for 12 h. The reaction mixture was filtered through celite and the filtrate was concentrated to provide 2-(4,4-difluoropiperidin-1-yl)aniline (405 mg, 1.91 mmol, 77% yield), which was used in the next step without further purification. ^{1}H NMR (300 MHz, $CDCl_3$) δ 2.02-2.21 (4H, m), 3.01 (4H, br t, J = 5.5 Hz), 3.95 (2H, br s), 6.70-6.78 (2H, m), 6.94 (1H, d, J = 7.5 Hz), 6.97-7.04 (1H, m). ESI-MS $[M+H]^+$ calcd. for $C_{11}H_{14}F_2N_2$: 213.1, found: 213.0.

1-(2-(4,4-difluoropiperidin-1-yl)phenyl)piperazine

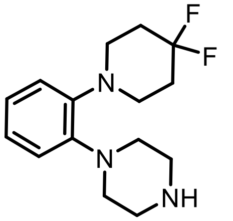

A mixture of 2-(4,4-difluoropiperidin-1-yl)aniline (200 mg, 0.94 mmol), 2-Chloro-*N*-(2-chloroethyl)ethanamine hydrochloride (168 mg, 0.94 mmol) and 2-(2-methoxyethoxy)ethanol (5 ml, 0.94 mmol) under an N_2 atmosphere was heated to 150 °C for 12 h. The reaction mixture was cooled to room temperature and diluted with MeOH (5 mL) followed by addition of Et_2O (100 mL). The resulting precipitate was isolated by filtration and washed with Et_2O to provide the product as an HCl salt. The HCl salt was converted to the free base amine by treatment with Na_2CO_3 solution and extraction with EtOAc (2 × 50 ml). The combined organic layers were dried over Na_2SO_4, and concentrated to provide 1-(2-(4,4-difluoropiperidin-1-yl)phenyl)piperazine (193 mg, 73% yield), which was used without further purification. ESI-MS $[M+H]^+$ calcd. For $C_{15}H_{21}F_2N_3$: 282.2, found 282.1.

(4-(2-(4,4-difluoropiperidin-1-yl)phenyl)piperazin-1-yl)(imidazo[1,5-a]pyridin-8-yl)methanone (9)

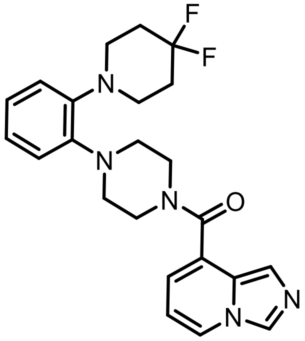

To a solution of 1-(2-(4,4-difluoropiperidin-1-yl)phenyl)piperazine (50 mg, 0.18 mmol) and imidazo[1,5-a]pyridine-8-carboxylic acid (58 mg, 0.36 mmol) in ethyl acetate (8 mL) was added a 50 wt. % solution of propanephosphonic acid anhydride in ethyl acetate (0.056 ml, 0.19 mmol) and DIPEA (0.031 ml, 0.18 mmol) at room temperature. The mixture was stirred at reflux for 4 h and then the mixture was filtered. The filtrate was concentrated and the residue was triturated with EtOAc/hexane. The product was purified by prep-HPLC (column: Phenomenex C18 75 x 30 mm x 3 um; mobile phase: [water (FA)-ACN]; B%: 10%-90%, 4 min). The title compound (7.8 mg, 8.1% yield) was isolated as a yellow solid. ^{1}H NMR (300 MHz, $CDCl_3$) δ 2.01-2.24 (4H, m), 3.13-3.30 (8H, m), 3.62-4.00 (4H, m), 6.92-7.15 (6H, m), 7.83 (1H, s), 8.31 (1H, br d, J = 7.1 Hz), 9.36 (1H, br s). ESI-MS [M+H]+ calcd. for $C_{23}H_{25}F_2N_5O$: 426.2, found: 426.2.

(*E*)-*N*-methyl-*N*-(2-nitrovinyl)aniline

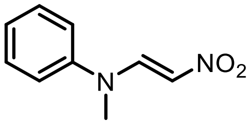

To a mixture of *N*-methylaniline (40.0 g, 373 mmol) in triethoxy methane (83.0 g, 560 mmol) was added CH_3NO_2 (49.0 g, 798 mmol) and TsOH.H_2O (710 mg, 3.73 mmol). The mixture was stirred at 110 °C for 4 h under N_2 atmosphere. The mixture was cooled to 0 °C and then the mixture was filtered via a filter paper. The filtered solid was washed with PE (100 mL x2) and then dried under reduced pressure. The title compound (23.0 g, 35% yield) was obtained as a yellow solid, which was used in the next step without further purification. ^{1}H NMR ($CDCl_3$, 400MHz) δ_H= 8.47 (d, J = 10.8 Hz, 1H), 7.42 (d, J = 7.6 Hz, 2H), 7.27-7.25 (m, 1H), 7.19 (d, J = 8.0 Hz, 2H), 6.83 (d, J = 10.0 Hz, 1H), 3.31 (s, 3H).

(*E*)-2-nitroethenamine

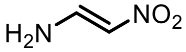

NH_3 was bubbled into in DCM (1000 mL) at -60 °C for 30 minutes. Then a solution of (*E*)-*N*-methyl-*N*-(2-nitrovinyl)aniline (23.0 g, 129 mmol) in DCM (200 mL) was added into the mixture dropwise at -60 °C. The mixture was stirred at -60 °C for 4 h. Excess NH_3 was purged by N_2. The mixture was filtered via a filter paper and the filtered solid was washed with PE (100 mL x2). The filtered solid was dried under reduced pressure. The title compound (10 g, 88% yield) was obtained as a yellow solid, which was used directly to the next step without further purification. ^{1}H NMR (DMSO-d_6 400MHz) δ_H= 8.76-8.52 (m, 2H), 7.11-7.03 (m, 1H), 6.39 (d, J = 6.8 Hz, 1H).

(*Z*)-*tert*-butyl (2-nitrovinyl)carbamate

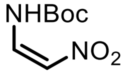

To a mixture of (*E*)-2-nitroethenamine (10.00 g, 113.55 mmol) in DCM (200 mL) was added DMAP (694 mg, 5.68 mmol) and Boc_2O (32.2 g, 148 mmol) at 0 °C. The mixture was allowed to warm up to room temperature and was stirred for 10 h. The mixture was diluted with DCM (200 mL), washed with 2N HCl (100 mL x 2) and brine (50 mL x 3). The organic layer was dried over Na_2SO_4 and concentrated to give crude product. The crude product was triturated with PE (200 mL) at 0 °C. The title compound was obtained as a yellow solid (20 g, 94% yield). ^{1}H NMR (DMSO-d_6 400MHz) δ_H= 10.67 (s, 1H), 8.16 (d, J = 10.8 Hz, 1H), 7.13 (d, J = 11.6 Hz, 1H), 1.48 (s, 9H).

***tert*-butyl (2-nitroethyl)carbamate**

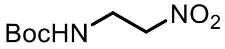

To a mixture of (*Z*)-tert-butyl (2-nitrovinyl)carbamate (19.00 g, 100.97 mmol) in MeOH (200 mL) was added $NaBH_4$ (38.7 g, 1.02 mol) in portions at 0°C. The mixture was allowed to warm up to room temperature and was stirred for 1 h. The mixture was quenched with water (100 mL) and extracted with DCM (80 mL x 3). The combined organic extracts were dried over Na_2SO_4 and concentrated to give crude product. The title compound was obtained as a brown oil (15 g, 78% yield) which was used in the next step without further purification. ^{1}H NMR (DMSO-d_6 400MHz) δ_H= 7.15 (s, 1H), 4.61-4.53 (m, 2H), 3.52-3.30 (m, 2H), 1.38 (s, 9H).

(*trans*)-*tert*-butyl 3-nitro-4-phenyl-3,4-dihydropyridine-1(2*H*)-carboxylate

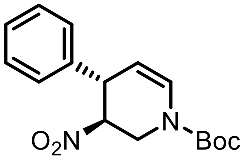

To a mixture of *tert*-butyl (2-nitroethyl)carbamate (8.63 g, 45.4 mmol) in DCM (60 mL) was added benzoic acid (554 mg, 4.54 mmol), [diphenyl-[(2*R*)-pyrrolidin-2-yl]methoxy]-trimethyl-silane (591 mg, 1.82 mmol) and (*E*)-3-phenylprop-2-enal (3.00 g, 22.7 mmol). The mixture was stirred at room

temperature for 10 h. The mixture was diluted with DCM (4 mL), then TFA (5.18 g, 45.4 mmol) was added into the mixture at 0 °C. The mixture was stirred at room temperature for 5 h. The mixture was diluted with DCM (80 mL) and washed with saturated aqueous $NaHCO_3$ (50 mL x 2). The organic layer was dried over Na_2SO_4 and concentrated to give crude product. The title compound was obtained as a yellow oil (8.0 g, crude) which was used in the next step without further purification. ESI-MS [M-100+H]$^+$ calcd. for $C_{16}H_{20}N_2O_4$: 205.3, found: 205.2.

(*trans*)-3-nitro-4-phenylpiperidine

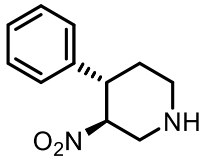

To a mixture of (*trans*)-*tert*-butyl 3-nitro-4-phenyl-3,4-dihydropyridine-1(2*H*)-carboxylate (8.00 g, 26.3 mmol) in DCM (100 mL) was added Et_3SiH (6.11 g, 52.6 mmol) and TFA (18.0 g, 158 mmol) at 0 °C. The mixture was stirred at room temperature for 5 h. The mixture was concentrated to give a residue, which was diluted with water (100 mL) and extracted with PE (80 mL x 3). The pH of the aqueous phase was adjusted to 8 with saturated aqueous $NaHCO_3$. The mixture was extracted with DCM (100 mL x 3). The combined organic extracts were dried over Na_2SO_4 and concentrated to give product. The title compound was obtained as a yellow oil (2.80 g, 39% yield for 2 steps) which was used in the next step without further purification. ESI-MS [M+H]$^+$ calcd for $C_{11}H_{14}N_2O_2$, 207.24, found 207.2.

imidazo[1,5-a]pyridin-8-yl((*trans*)-3-nitro-4-phenylpiperidin-1-yl)methanone

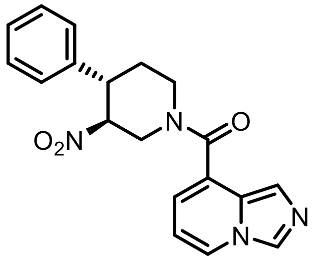

To a mixture of (*trans*)-3-nitro-4-phenylpiperidine (1.50 g, 7.27 mmol) and imidazo[1,5-a]pyridine-8-carboxylic acid (708 mg, 4.36 mmol) in pyridine (10 mL) was added EDCI (2.79 g, 14.6 mmol). The mixture was stirred at 50 °C for 1 h. The mixture was diluted with DCM (20 mL), washed with 1N HCl (10 mL x 2) and saturated aqueous $NaHCO_3$ (10 mL x 2). The combined organic layer was dried over Na_2SO_4 and concentrated to give crude product. The crude product was purified by silica gel flash chromatography (10% MeOH in DCM). The title compound was obtained as a yellow oil (300 mg, 20% yield) which was used directly to the next step without further purification. ^{1}H NMR (DMSO-d_6 400MHz) δ_H= 8.45 (s, 1H), 8.43-8.41 (m, 1H), 7.41-7.38 (m, 2H), 7.36-7.24 (m, 4H), 7.01-6.85 (m, 1H), 6.77-6.72 (m, 1H), 5.23-4.97 (m, 2H), 3.31-3.07 (m, 4H), 1.83-1.57 (m, 2H).

((*trans*)-3-amino-4-phenylpiperidin-1-yl)(imidazo[1,5-a]pyridin-8-yl)methanone

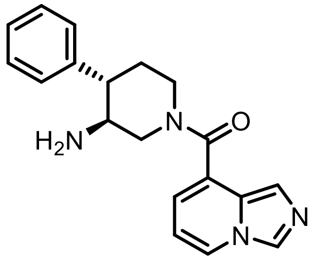

To a mixture of imidazo[1,5-a]pyridin-8-yl((*trans*)-3-nitro-4-phenylpiperidin-1-yl)methanone (300 mg, 0.86 mmol) in EtOH (3 mL) and water (3 mL) was added Fe (143 mg, 2.57 mmol) and NH_4Cl (137 mg,

2.57 mmol). The mixture was stirred at 80 °C for 2 h. The mixture was filtered through a celite pad and the pad was washed with DCM/MeOH (10:1, 30 mL x 3). The filtrate was washed with brine (30 mL x 2). The organic layer was dried over Na_2SO_4 and concentrated to give crude product. The product was purified by silica gel flash chromatography (50% MeOH in DCM). The title compound was obtained as a yellow solid (80 mg, 26% yield). ^{1}H NMR (DMSO-d_6 400MHz) δ_H= 8.47 (s, 1H), 8.40 (d, J = 6.8 Hz, 1H), 7.37-7.22 (m, 6H), 6.88-6.84 (m, 1H), 6.75-6.71 (m, 1H), 4.70-4.41 (m, 1H), 3.62-3.49 (m, 1H). 3.27-2.79 (m, 4H), 1.97-1.56 (m, 2H), 1.47-1.16 (m, 2H).

N-((*trans*)-1-(imidazo[1,5-a]pyridine-8-carbonyl)-4-phenylpiperidin-3-yl)acetamide (10)

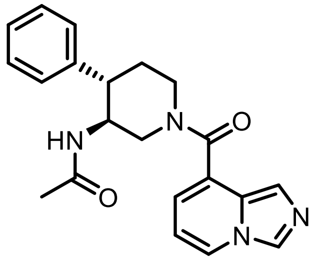

To a mixture of ((*trans*)-3-amino-4-phenylpiperidin-1-yl)(imidazo[1,5-a]pyridin-8-yl)methanone (40 mg, 0.12 mmol) and TEA (38 mg, 0.37 mmol) in DCM (2 mL) was added $(CH_3CO)_2O$ (15 mg, 0.15 mmol) at 0 °C. The mixture was allowed to warm up to room temperature and was stirred for 2 h. The mixture was concentrated to give crude product. The product was purified by prep-HPLC (column: Phenomenex Gemini-NX C18 75 × 30 mm × 3 µm; mobile phase: [water (10mM NH4HCO3)-ACN]; B%: 12%-42%, 10 min). The title compound (13.1 mg, 29% yield) was obtained as a light yellow solid. ^{1}H NMR (CD_3OD 400MHz) δ_H= 8.47-8.36 (m, 2H), 7.77-7.65 (m, 1H), 7.37-7.16 (m, 6H), 6.93 (d, J = 7.2 Hz, 1H), 6.72 (t, J = 6.8 Hz, 1H), 4.67-4.63 (m, 1H), 4.03-3.95 (m, 1H), 3.87-3.36 (m, 1H), 3.23-2.76 (m, 1H), 2.67-2.50 (m, 1H), 1.67-1.53 (m, 5H). ESI-MS $[M+H]^+$ calcd. for $C_{21}H_{22}N_4O_2$: 363.2, found: 363.1.

tert-butyl ((*S*)-1-(((*trans*)-1-(imidazo[1,5-a]pyridine-8-carbonyl)-4-phenylpiperidin-3-yl)amino)-3,3-dimethyl-1-oxobutan-2-yl)carbamate

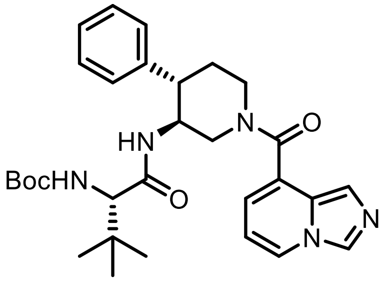

To a mixture of ((*trans*)-3-amino-4-phenylpiperidin-1-yl)(imidazo[1,5-a]pyridin-8-yl)methanone (500 mg, 1.56 mmol) and (2*S*)-2-(*tert*-butoxycarbonylamino)-3,3-dimethyl-butanoic acid (722 mg, 3.12 mmol) in DMF (6 mL) was added PyBOP (1.22 g, 2.34 mmol) and DIEA (807 mg, 6.24 mmol) at room temperature. The mixture was stirred at room temperature for 2 h. The reaction mixture was diluted with water (20 mL) and extracted with DCM (40 mL x 3). The combined organic extracts were washed with water (30 mL x 3), dried over Na_2SO_4, and concentrated to give crude product. The product was purified by silica gel flash chromatography (100% EtOAc). The title compound (800 mg, 85% yield) was obtained as yellow solid. ESI-MS $[M+H]^+$ calcd. for $C_{30}H_{39}N_5O_4$: 534.3, found: 534.3.

(*S*)-2-acetamido-*N*-((*trans*)-1-(imidazo[1,5-a]pyridine-8-carbonyl)-4-phenylpiperidin-3-yl)-3,3-dimethylbutanamide (11)

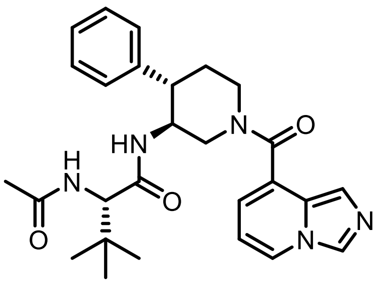

To a mixture of *tert*-butyl ((*S*)-1-(((*trans*)-1-(imidazo[1,5-a]pyridine-8-carbonyl)-4-phenylpiperidin-3-yl)amino)-3,3-dimethyl-1-oxobutan-2-yl)carbamate (800 mg, 1.50 mmol) in DCM (3 mL) was added HCl/dioxane (4 M, 10 mL) at room temperature. The mixture was stirred at room temperature for 1 h. The reaction mixture was concentrated directly providing a black/brown solid. The crude deprotected intermediate (700 mg, crude) was used in the next step without purification.

To a mixture of crude deprotected intermediate (60 mg, crude) in DCM (2 mL) was added TEA (42 mg, 0.42 mmol) and $(CH_3CO)_2O$ (19 mg, 0.18 mmol) at 0°C. The mixture was stirred at room temperature for 2 h. The mixture was concentrated directly and the product was purified by prep-HPLC (column: Welch Xtimate C18 150 x 25 mm x 5 um; mobile phase: [water (NH_4HCO_3)-ACN]; B%: 20%-55%, 12 min). The title compound (20.3 mg, 33% yield over 2 steps) was obtained as a light yellow solid**.** ^{1}H NMR (DMSO-d_6, 400MHz) δ_H = 8.53-8.32 (m, 2H), 8.02-7.67 (m, 1H), 7.65-7.52 (m, 1H), 7.36 (s, 1H), 7.29-7.10 (m, 5H), 7.00-6.88 (m, 1H), 6.71 (t, *J* = 6.8 Hz, 1H), 4.62 (s, 1H), 4.18-3.88 (m, 2H), 3.61-3.47 (m, 1H), 3.21-3.02 (m, 1H), 3.01-2.74 (m, 2H), 1.92-1.52 (m, 5H), 1.02-0.36 (m, 9H). ESI-MS $[M+H]^+$ calcd. for $C_{27}H_{33}N_5O_3$: 476.3, Found: 476.4.

***N*-((*trans*)-1-(imidazo[1,5-a]pyridine-8-carbonyl)-4-phenylpiperidin-3-yl)-1*H*-pyrrole-2-carboxamide (12)**

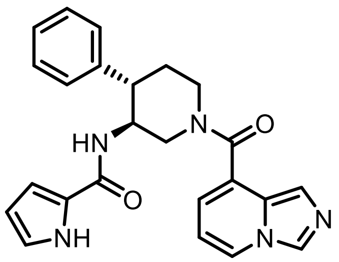

To a mixture of ((*trans*)-3-amino-4-phenylpiperidin-1-yl)(imidazo[1,5-a]pyridin-8-yl)methanone (40 mg, 0.12 mmol) and 1*H*-pyrrole-2-carboxylic acid (21 mg, 0.19 mmol) in pyridine (2 mL) was added EDCI (48 mg, 0.25 mmol). The mixture was stirred at 50 °C for 2 h. The mixture was diluted with DCM (20 mL) and washed with brine (30 mL x 2). The organic layer was dried over Na_2SO_4 and concentrated to give crude product. The product was purified by prep-HPLC (column: Phenomenex Gemini-NX C18 75×30mm×3µm; mobile phase: [water (10mM NH_4HCO_3)-ACN]; B%: 20%-50%, 10min). The title

compound (18.4 mg, 36% yield) was obtained as a light yellow solid. ^{1}H NMR (DMSO-d_6, 400MHz) δ_H= 11.35-11.17 (m, 1H), 8.52-8.37 (m, 2H), 7.80-7.72 (m, 1H), 7.41-7.10 (m, 6H), 6.98-6.93 (m, 1H), 6.76-6.53 (m, 3H), 6.09-5.94 (m, 1H). 4.73-4.62 (m, 1H), 4.27-4.16 (m, 1H), 3.85-3.50 (m, 1H), 3.27-2.63 (m, 3H), 2.08-1.73 (m, 2H). ESI-MS [M+H]$^+$ calcd. for $C_{24}H_{23}N_5O_2$: 414.2, found: 414.1.

***N*-((*trans*)-1-(imidazo[1,5-a]pyridine-8-carbonyl)-4-phenylpiperidin-3-yl)-1*H*-imidazole-2-carboxamide (13)**

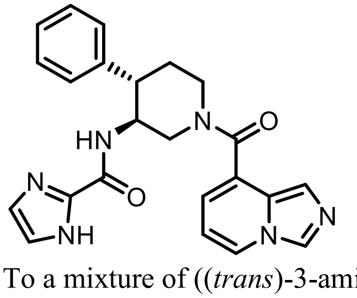

To a mixture of ((*trans*)-3-amino-4-phenylpiperidin-1-yl)(imidazo[1,5-a]pyridin-8-yl)methanone (30 mg, 0.09 mmol) and 1*H*-imidazole-2-carboxylic acid (21 mg, 0.19 mmol) in DMF (2 mL) was added DIEA (60 mg, 0.47 mmol) and PyBOP (73 mg, 0.14 mmol). The reaction mixture was stirred at room temperature for 12 h. The reaction mixture was concentrated directly to give crude product. The crude product was purified by prep-HPLC (column: Phenomenex C18 75 × 30 mm × 3 µm; mobile phase: [water (NH_4HCO_3)-ACN]; B%: 17%-47%, 10 min). The title compound (15.0 mg, 39% yield) was obtained as a light yellow solid. ^{1}H NMR (CD_3OD, 400 MHz) δ_H =8.44 (s, 1H), 8.33 (d, J = 6.8 Hz, 1H), 7.47 (s, 1H), 7.37-7.31 (m, 2H), 7.30-7.23 (m, 2H), 7.22-6.91 (m, 4H), 6.80 (d, J = 6.8 Hz, 1H), 4.5-3.5 (m, 3H), 3.16-2.91 (m, 2H), 2.19-1.70 (m, 2H), 1.29 (d, J = 6.4 Hz, 1H). ESI-MS [M+H]$^+$ calcd. for $C_{23}H_{22}N_6O_2$: 415.2; found: 415.4.

(*trans*)-*tert*-butyl 4-(3-chlorophenyl)-3-nitro-3,4-dihydropyridine-1(2*H*)-carboxylate

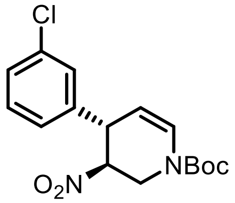

To a mixture of tert-butyl (2-nitroethyl)carbamate (4.57 g, 24.0 mmol) in DCM (40 mL) was added benzoic acid (293 mg, 2.40 mmol), [diphenyl-[(2*R*)-pyrrolidin-2-yl] methoxy]-trimethyl-silane (313 mg, 0.96 mmol) and (*E*)-3-(3-chlorophenyl) prop-2-enal (2.00 g, 12.0 mmol). The mixture was stirred at room temperature for 10 h. The mixture was diluted with DCM (40 mL), then TFA (2.74 g, 24.0 mmol) was added to the mixture at 0 °C. The mixture was stirred at room temperature for 5 h. The mixture was diluted with DCM (100 mL) and washed with saturated aqueous $NaHCO_3$ (100 mL x 2). The organic layer was dried over Na_2SO_4 and concentrated to give crude product (7.30 g, crude), which was used in the next step without further purification. ESI-MS [M-100+H]$^+$ calcd. for $C_{16}H_{19}ClN_2O_4$: 239.1, found: 239.1.

(*trans*)-4-(3-chlorophenyl)-3-nitropiperidine

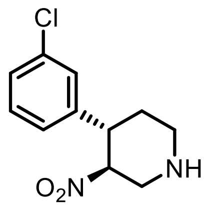

To a mixture of (*trans*)-*tert*-butyl 4-(3-chlorophenyl)-3-nitro-3,4-dihydropyridine-1(2*H*)-carboxylate (7.20 g, 21.3 mmol) in DCM (90 mL) was added Et_3SiH (4.94 g, 42.5 mmol) and TFA (14.5 g, 128 mmol) at 0 °C. The mixture was stirred at room temperature for 5 h. The mixture was concentrated to give a crude residue which was diluted with water (100 mL) and extracted with PE (80 mL x 3). The pH of the aqueous phase was adjusted to 8 with saturated aqueous $NaHCO_3$. The resulting mixture was extracted with DCM (130 mL x3). The combined organic extracts were dried over Na_2SO_4 and concentrated to give crude product as a black brown oil The (3.40 g, 49% yield for 2 steps), which was used directly in the next step without further purification. ESI-MS $[M+H]^+$calcd. for $C_{11}H_{13}ClN_2O_2$, 241.1, found 241.1.

((*trans*)-4-(3-chlorophenyl)-3-nitropiperidin-1-yl)(imidazo[1,5-a]pyridin-8-yl)methanone

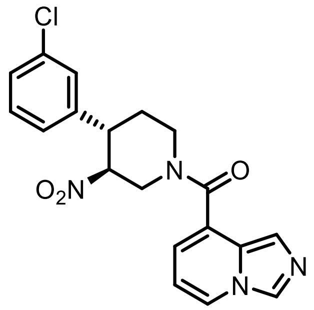

To a mixture of (*trans*)-4-(3-chlorophenyl)-3-nitropiperidine (500 mg, 2.08 mmol) and imidazo[1,5-a]pyridine-8-carboxylic acid (337 mg, 2.08 mmol) in DMF (15 mL) was added PyBOP (1.62 g, 3.12 mmol) and DIEA (805 mg, 6.23 mmol). The mixture was stirred at room temperature for 2 h. The mixture was diluted with water (50 mL) and extracted with EtOAc (50 mL x 3). The combined organic extracts were dried over Na_2SO_4 and concentrated to give crude product. The product was purified by silica gel flash chromatography (100% EtOAc). The title compound (580 mg, 55% yield) was obtained as a black brown oil. ^{1}H NMR (DMSO-d_6, 400MHz) δ_H= 8.49 (s, 1H), 8.46-8.38 (m, 1H), 7.61 (s, 1H), 7.51 (s, 1H), 7.40-7.24 (m, 4H), 6.76 (t, J = 6.8 Hz, 1H), 3.67-3.40 (m, 3H), 3.34-3.18 (m, 3H), 1.90-1.72 (m, 2H).

((*trans*)-3-amino-4-(3-chlorophenyl)piperidin-1-yl)(imidazo[1,5-a]pyridin-8-yl)methanone

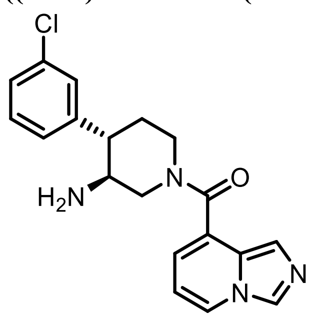

To a mixture of ((*trans*)-4-(3-chlorophenyl)-3-nitropiperidin-1-yl)(imidazo[1,5-a]pyridin-8-yl)methanone (580 mg, 1.51 mmol) in EtOH (6 mL) and water (6 mL) was added NH_4Cl (242 mg, 4.52 mmol) and Fe (253 mg, 4.52 mmol) at room temperature. The mixture was stirred at 100 °C for 4 h. The mixture was filtered via a celite pad. The pad washed with (DCM:MeOH=1:1, 30 ml x 5). The filtrate was concentrated to give product. The crude product was purified by silica gel flash chromatography on silica

gel (9% MeOH in DCM). The title compound (110 mg, 19% yield) was obtained as a yellow solid. ESI-MS [M+H]$^+$calcd. for $C_{19}H_{19}ClN_4O$: 355.1, found: 355.1.

_N_-((_trans_)-4-(3-chlorophenyl)-1-(imidazo[1,5-a]pyridine-8-carbonyl)piperidin-3-yl)-1H-imidazole-2-carboxamide (15)

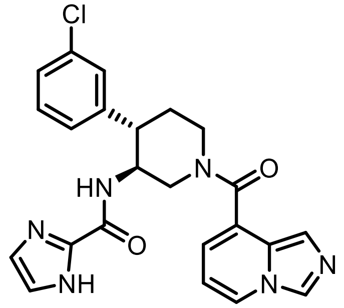

To a mixture of ((*trans*)-3-amino-4-(3-chlorophenyl)piperidin-1-yl)(imidazo[1,5-a]pyridin-8-yl)methanone (55 mg, 0.16 mmol) and 1*H*-imidazole-2-carboxylic acid (17 mg, 0.16 mmol) in DMF (3 mL) was added PyBOP (121 mg, 0.23 mmol) and DIEA (60 mg, 0.47 mmol). The mixture was stirred at room temperature for 2 h. The mixture was diluted with water (20 mL) and extracted with DCM (30 mL x 3). The combined organic extracts were dried over Na_2SO_4 and concentrated to give crude product. The product was purified by prep-HPLC (Phenomenex C18 75 × 30 mm × 3 μm; mobile phase: [water (NH_4HCO_3)-ACN]; B%: 22%-52%, 10 min). The title compound (29.4 mg, 41% yield) was obtained as a yellow solid. ^{1}H NMR (CD_3OD, 400MHz) δ_H= 8.43 (s, 1H), 8.33 (d, J = 7.2 Hz, 1H), 7.48 (s, 1H), 7.40-7.35 (m, 1H), 7.31-7.00 (m, 6H), 6.79 (t, J = 6.8 Hz, 1H), 4.71-4.17 (m, 2H), 4.10-3.55 (m, 1H), 3.32-2.84 (m, 3H), 2.18-1.73 (m, 2H). ESI-MS [M+H]$^+$ calcd. for $C_{23}H_{21}ClN_6O_2$: 449.1, found: 449.3.

(_E_)-3-(3-chloro-5-fluorophenyl)acrylaldehyde

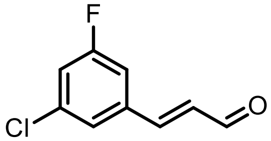

A solution of 3-chloro-5-fluorobenzaldehyde (5.00 g, 31.5 mmol) and 2-(triphenyl-λ5-phosphanylidene)acetaldehyde (10.6 g, 34.7 mmol) in toluene (50 mL) was degassed and purged with N_2, and then the mixture was stirred at 100 °C for 16 h under N_2 atmosphere. The mixture was concentrated to a crude residue and the product was purified by silica gel flash chromatography (0-10% EtOAc in PE. The title compound (4.10 g, 65% yield) was obtained as a yellow solid. ^{1}H NMR ($CDCl_3$, 400MHz) δ_H= 9.72 (d, J = 7.6 Hz, 1H), 7.39-7.33 (m, 2H), 7.17 (dd, J = 1.2, 8.8 Hz, 2H), 6.69 (dd, J = 7.6, 16.0 Hz, 1H)

(_trans_)-_tert_-butyl 4-(3-chloro-5-fluorophenyl)-3-nitro-3,4-dihydropyridine-1(2_H_)-carboxylate

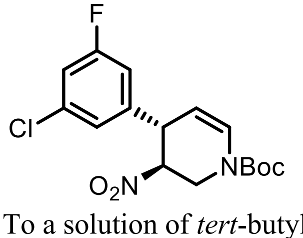

To a solution of *tert*-butyl *N*-(2-nitroethyl)carbamate (8.45 g, 44.4 mmol) in DCM (45 mL) was added benzoic acid (543 mg, 4.44 mmol), [diphenyl-[(2*R*)-pyrrolidin-2-yl]methoxy]-trimethyl-silane (579 mg, 1.78 mmol) and (*E*)-3-(3-chloro-5-fluorophenyl)acrylaldehyde (4.10 g, 22.2 mmol). The mixture was stirred at room temperature for 10 h. The mixture was diluted with DCM (45 mL), then TFA (5.07 g, 44.4 mmol) was added was added into the reaction mixture at 0°C. The mixture was stirred at room

temperature for 5 hrs. The mixture was diluted with DCM (50 mL) and washed with saturated aqueous $NaHCO_3$ (50 mL x 3). The organic layer was dried over Na_2SO_4 and concentrated. The title compound (12.7 g, crude) was obtained as a brown oil and was used without further purification. ESI-MS $[M+H-100]^+$ calcd. for $C_{16}H_{18}ClFN_2O_4$: 257.1; found: 257.1.

(*trans*)-4-(3-chloro-5-fluorophenyl)-3-nitropiperidine

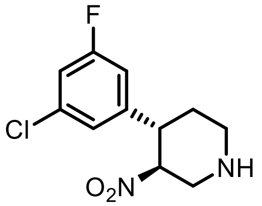

To a solution of (*trans*)-*tert*-butyl 4-(3-chloro-5-fluorophenyl)-3-nitro-3,4-dihydropyridine-1(2*H*)-carboxylate (12.7 g, 35.6 mmol) in DCM (130 mL) was added Et_3SiH (8.28 g, 71.2 mmol) and TFA (24.4 g, 214 mmol) at 0 °C. The mixture was stirred at room temperature for 5 h. The mixture was concentrated to a crude residue. The residue was diluted with water (100 mL) and extracted with PE (100 mL x3). The pH of the aqueous phase was adjusted to 8 with saturated aqueous $NaHCO_3$. The mixture was extracted with DCM (300 mL x 3). The combined organic extracts were dried over Na_2SO_4 and concentrated. The title compound (6.70 g, 73% yield over 2 steps) was obtained as a brown oil and used in the next step without purification. ESI-MS [M+H]+ calcd. for $C_{11}H_{12}ClFN_2O_2$: 259.1; found: 259.0.

((*trans*)-4-(3-chloro-5-fluorophenyl)-3-nitropiperidin-1-yl)(imidazo[1,5-a]pyridin-8-yl)methanone

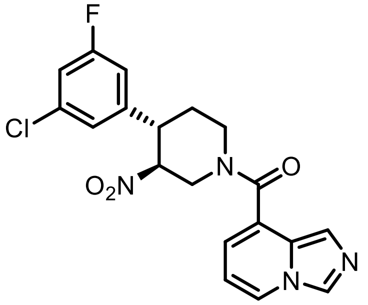

To a solution of (*trans*)-4-(3-chloro-5-fluorophenyl)-3-nitropiperidine (3.00 g, 11.6 mmol) and imidazo[1,5-a]pyridine-8-carboxylic acid (1.32 g, 8.12 mmol) in DMF (30 mL) was added PyBOP (7.85 g, 15.1 mmol) and DIEA (6.00 g, 46.4 mmol).The mixture was stirred at room temperature for 1 h. The mixture was diluted with EtOAc (60 mL) and washed with brine (30 mL). The organic layer was dried over anhydrous Na_2SO_4 and concentrated to crude residue. The product was purified by silica gel flash chromatography (0-12% MeOH in DCM). The title compound (5.70 g, 77% yield) was obtained as a brown oil. ESI-MS [M+H]+ calcd. for $C_{19}H_{16}ClFN_4O_3$: 403.1; found: 403.1

((*trans*)-3-amino-4-(3-chloro-5-fluorophenyl)piperidin-1-yl)(imidazo[1,5-a]pyridin-8-yl)methanone

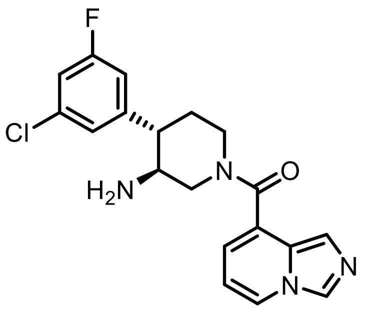

To a solution of ((*trans*)-4-(3-chloro-5-fluorophenyl)-3-nitropiperidin-1-yl)(imidazo[1,5-a]pyridin-8-yl)methanone (4.00 g, 9.93 mmol) in EtOH (50 mL) and water (50 mL) was added Fe (1.66 g, 29.8 mmol) and NH_4Cl (1.59 g, 29.8 mmol). The mixture was stirred at 100 °C for 2 h. The mixture was filtered through a celite pad and the pad was washed with DCM/MeOH (10:1, 100 mL x 3). The filtrate was washed with brine (100 mL x 2). The organic layer was dried over Na_2SO_4 and concentrated. The product was purified by silica gel flash chromatography (0-15% MeOH in DCM). The title compound (1.20 g, 30% yield) was obtained as a yellow solid. ESI-MS $[M+H]^+$ calcd. for $C_{19}H_{18}ClFN_4O$: 373.1; found: 373.1.

***tert*-butyl ((3*S*,4*S*)-4-(3-chloro-5-fluorophenyl)-1-(imidazo[1,5-a]pyridine-8-carbonyl)piperidin-3-yl)carbamate**

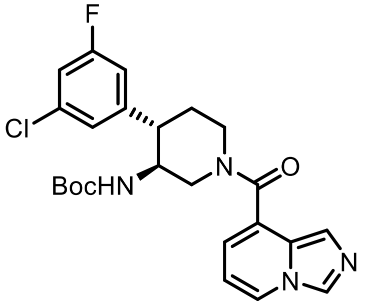

To a solution of ((*trans*)-3-amino-4-(3-chloro-5-fluorophenyl)piperidin-1-yl)(imidazo[1,5-a]pyridin-8-yl)methanone (1.20 g, 3.22 mmol) in DCM (13 mL) was added Boc_2O (1.05 g, 4.83 mmol) and TEA (1.63 g, 16.1 mmol). The mixture was stirred at room temperature for 2 h. The mixture was diluted with DCM (30 mL), washed with saturated aqueous NH_4Cl (20 mL x 3). The organic layer was dried over Na_2SO_4 and concentrated. The residue was purified by silica gel flash chromatography (0-10% MeOH in DCM). The product was further purified by chiral SFC (condition: column: DAICEL CHIRALPAK AS (250mm×30mm, 10μm); mobile phase: [0.1%NH_3H_2O ETOH]; B%: 40%-40%, min). The title compound (480 mg, 28% yield) was obtained as a yellow solid. ^1H NMR ($CDCl_3$, 400MHz) δ_H= 8.58 (s, 1H), 8.13 (d, J = 6.8 Hz, 1H), 7.53 (s, 1H), 7.05-6.92 (m, 3H), 6.86 (d, J = 9.2 Hz, 1H), 6.74 (s, 1H), 3.62 (d, J = 1.2 Hz, 1H), 3.09-2.79 (m, 3H), 2.60 (s, 3H), 2.08-1.89 (m, 1H), 1.77 (d, J = 2.8 Hz, 1H), 1.34-1.22 (m, 9H).

((3*S*,4*S*)-3-amino-4-(3-chloro-5-fluorophenyl)piperidin-1-yl)(imidazo[1,5-a]pyridin-8-yl)methanone

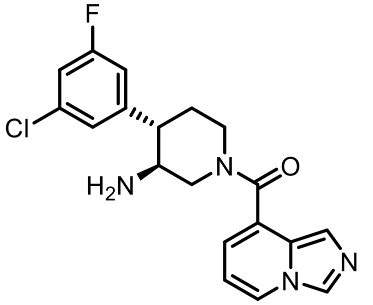

To a solution of tert-butyl ((3*S*,4*S*)-4-(3-chloro-5-fluorophenyl)-1-(imidazo[1,5-a]pyridine-8-carbonyl)piperidin-3-yl)carbamate (380 mg, 0.80 mmol) in dioxane (4 mL) was added HCl/dioxane (4 M, 4 mL) at. The mixture was stirred at room temperature for 1 h. The mixture was concentrated and the title compound (300 mg, crude) was used without purification.

***N*-((3*S*,4*S*)-4-(3-chloro-5-fluorophenyl)-1-(imidazo[1,5-a]pyridine-8-carbonyl)piperidin-3-yl)-1*H*-imidazole-2-carboxamide (21)**

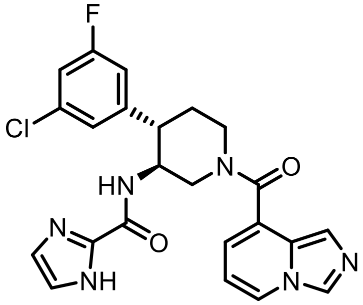

To a mixture of ((3*S*,4*S*)-3-amino-4-(3-chloro-5-fluorophenyl)piperidin-1-yl)(imidazo[1,5-a]pyridin-8-yl)methanone (30 mg, 0.81 mmol) and 1*H*-imidazole-2-carboxylic acid (9 mg, 0.81 mmol) in DMF (1 mL) was added PyBOP (55 mg, 0.105 mmol) and DIEA (42 mg, 0.322 mmol). The mixture was stirred at room temperature for 1 h. The mixture was concentrated to a crude residue. The residue was purified by prep-HPLC (column: C18-6 100 × 30mm × 5 µm; mobile phase: [water (FA)-ACN]; B%: 16%-46%, 15min). Compound 65 (8.3 mg, 22% yield over 2 steps) was obtained as a yellow solid. ^{1}H NMR (DMSO-*d$_6$*, 400MHz) δ_H= 13.12-12.75 (m, 1H), 8.60-8.22 (m, 3H), 7.43 (s, 1H), 7.34 (s, 1H), 7.30-7.11 (m, 3H), 6.98 (d, J = 6.4 Hz, 2H), 6.72 (t, J = 6.8 Hz, 1H), 4.83-4.52 (m, 1H), 4.46-3.98 (m, 1H), 3.24-2.79 (m, 4H), 1.95-1.67 (m, 2H). ESI-MS $[M+H]^+$ calcd. for $C_{23}H_{20}ClFN_6O_2$: 467.1; found: 467.1.

(*E*)-3-(3,5-dichlorophenyl)acrylaldehyde

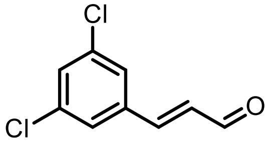

A solution of 3,5-dichlorobenzaldehyde (5.00 g, 31.5 mmol) and 2-(triphenyl-λ5-phosphanylidene)acetaldehyde (10.6 g, 34.7 mmol) in toluene (50 mL) was degassed and purged with N_2, and then the mixture was stirred at 80 °C for 12 h under N_2 atmosphere. The mixture was concentrated to a crude residue and the residue was purified by silica gel flash chromatography (0-3% EtOAc in PE). The title compound (4.16 g, 72% yield) was obtained as a brown oil. ^{1}H NMR (DMSO-*d$_6$*, 400MHz) δ_H = 9.67 (d, *J* = 7.6 Hz, 1H), 7.88 (d, *J* = 2.0 Hz, 2H), 7.72-7.68 (m, 2H), 7.03 (dd, *J* = 7.6, 16.0 Hz, 1H).

(*trans*)-*tert*-butyl 4-(3,5-dichlorophenyl)-3-nitro-3,4-dihydropyridine-1(2*H*)-carboxylate

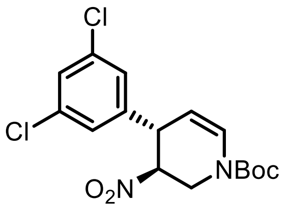

To a solution of *tert*-butyl *N*-(2-nitroethyl)carbamate (15.7 g, 82.7 mmol) in DCM (150 mL) was added (*E*)-3-(3,5-dichlorophenyl) prop-2-enal (4.16 g, 20.7 mmol), [diphenyl-[(2*R*)-pyrrolidin-2-yl]methoxy]-trimethyl-silane (538 mg, 1.65 mmol) and benzoic acid (505 mg, 4.14 mmol). The reaction mixture was stirred at room temperature for 12 h. Then a solution of TFA (4.72 g, 41.35 mmol) in DCM (50 mL) was added into the reaction at 0 °C and the reaction mixture was stirred at room temperature for 5 h. The reaction mixture was quenched with saturated aqueous $NaHCO_3$ (200 mL) and extracted with DCM (100 mL x 3). The combined organic extracts were dried over anhydrous Na_2SO_4, filtered and concentrated to give crude product. The crude product was used to the next step directly without purification. The title (22.0 g, crude) was obtained as a brown oil and used without purification.

(*trans*)-*tert*-butyl 4-(3,5-dichlorophenyl)-3-nitropiperidine-1-carboxylate

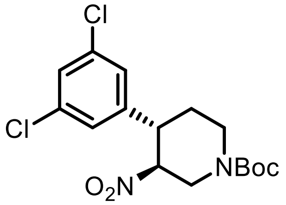

To a solution of (*trans*)-*tert*-butyl 4-(3,5-dichlorophenyl)-3-nitro-3,4-dihydropyridine-1(2*H*)-carboxylate (22.0 g, 80 mmol) in DCM (130 mL) was added Et_3SiH (18.6 g, 160 mmol) and TFA (54.7 g, 480 mmol) at 0 °C. The mixture was stirred at room temperature for 5 h. The mixture was concentrated to a crude residue. The residue was diluted with water (100 mL) and extracted with PE (100 mL x 3). The pH of the aqueous phase was adjusted to 8 with saturated aqueous $NaHCO_3$. The mixture was extracted with DCM (300 mL x 3). The combined organic extracts were dried over Na_2SO_4 and concentrated. The crude intermediate was used without purification

To a solution of the crude intermediate (5.91 g, 21.5 mmol) in DCM (500 mL) was added Boc_2O (9.38 g, 43.0 mmol) and TEA (6.52 g, 64.4 mmol). The mixture was stirred at room temperature for 12 h. The reaction mixture was diluted with water (60 mL) and extracted with DCM (40 mL x 3). The combined organic layers were dried over Na_2SO_4, filtered and concentrated to give a crude residue. The residue was purified by silica gel flash chromatography (0-6% Ethyl acetate in Petroleum ether). The title compound (1.27 g, 8.9% yield over 3 steps) was obtained as a green oil. ESI-MS $[M-56+H]^+$ calcd. for $C_{16}H_{20}Cl_2N_2O_4$: 319.1, found 319.0.

((*trans*)-4-(3,5-dichlorophenyl)-3-nitropiperidin-1-yl)(imidazo[1,5-a]pyridin-8-yl)methanone

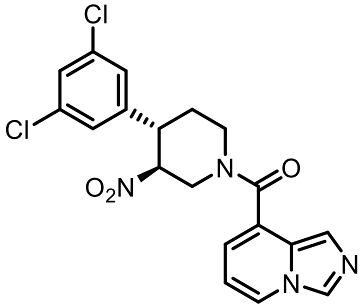

A mixture of (*trans*)-*tert*-butyl 4-(3,5-dichlorophenyl)-3-nitropiperidine-1-carboxylate (1.27 g, 3.38 mmol) in HCl/dioxane (4 M, 5 mL) was stirred at room temperature for 2 h under N_2 atmosphere. The reaction mixture was concentrated to provide the crude deprotected intermediate (1.26 g, crude) as a yellow solid, which was used without purification.

To a solution of the deprotected intermediate (1.16 g, 4.22 mmol) in DMF (10 mL) was added imidazo[1,5-a]pyridine-8-carboxylic acid (683 mg, 4.22 mmol), PyBOP (2.85 g, 5.48 mmol) and DIEA (2.72 g, 21.1 mmol). The mixture was stirred at room temperature for 2 h. The reaction mixture was diluted with water (40 mL) and extracted with EtOAc (60 mL). The combined organic extracts were washed with brine (60 mL), dried over Na_2SO_4, and concentrated to give a crude residue. The product was purified by silica gel flash chromatography (0-72% Ethyl acetate in PE. The title compound (616 mg, 35 % yield over 2 steps) was obtained as a yellow solid.

***tert*-butyl ((3*S*,4*S*)-4-(3,5-dichlorophenyl)-1-(imidazo[1,5-a]pyridine-8-carbonyl)piperidin-3-yl)carbamate**

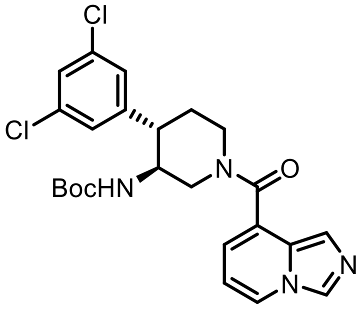

To a solution of ((*trans*)-4-(3,5-dichlorophenyl)-3-nitropiperidin-1-yl)(imidazo[1,5-a]pyridin-8-yl)methanone (616 mg, 1.47 mmol) in EtOH (5 mL) and water (5 mL) was added Fe (328 mg, 5.88 mmol) and NH_4Cl (314 mg, 5.88 mmol). The mixture was stirred at 100 °C for 2 h. The reaction mixture was filtered through a celite pad. The celite pad was washed with DCM and MeOH (1:1, 150 mL x 3). The filtrate was concentrated to give a crude intermediate, which was used without purification

To a solution of the crude intermediate in DCM (10 mL) was added Boc_2O (1.14 g, 5.21 mmol) and TEA (790 mg, 7.81 mmol). The mixture was stirred at room temperature for 12 h. The reaction mixture was diluted with water (40 mL) and extracted with DCM (80 mL). The combined organic extracts were dried over Na_2SO_4, filtered and concentrated to give a crude residue. The product was first purified by flash silica gel flash chromatography (0~87% Ethyl acetate in PE). Then the product was further purified by chiral SFC (column: DAICEL CHIRALPAK AD (250 mm x 30 mm, 10 um); mobile phase: [0.1%NH_3H_2O IPA]; B%: 40%-40%, min). The title compound (300 mg, 41% yield over 2 steps) was obtained as a brown solid. ^{1}H NMR (MeOD, 400MHz) δ_H = 8.45-8.30 (m, 2H), 7.45-7.35 (m, 4H), 6.94 (d, J = 4.0 Hz, 1H), 6.72 (t, J = 4.0 Hz, 1H), 4.75-4.25 (m, 1H), 3.80-3.45 (m, 2H), 3.10-2.70 (m, 3H), 1.90-1.65 (m, 2H), 1.23 (s, 9H).

((3*S*,4*S*)-3-amino-4-(3,5-dichlorophenyl)piperidin-1-yl)(imidazo[1,5-a]pyridin-8-yl)methanone

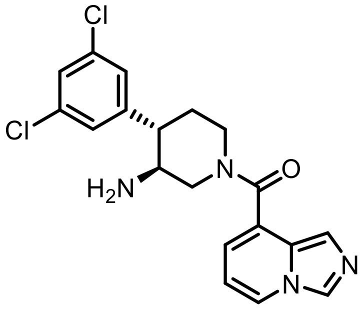

A mixture of *tert*-butyl ((3*S*,4*S*)-4-(3,5-dichlorophenyl)-1-(imidazo[1,5-a]pyridine-8-carbonyl)piperidin-3-yl)carbamate (300 mg, 0.61 mmol) in HCl/dioxane (4 M, 6 mL) was stirred at room temperature for 1 h. The reaction mixture was concentrated to provide the deprotected intermediate (300 mg, crude) as a white solid, which was used without purification.

***N*-((3*S*,4*S*)-4-(3,5-dichlorophenyl)-1-(imidazo[1,5-a]pyridine-8-carbonyl)piperidin-3-yl)-1*H*-imidazole-2-carboxamide (23)**

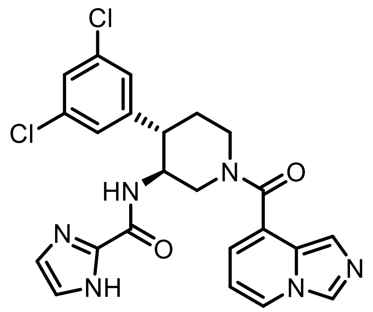

To a solution of ((3*S*,4*S*)-3-amino-4-(3,5-dichlorophenyl)piperidin-1-yl)(imidazo[1,5-a]pyridin-8-yl)methanone (30 mg, 0.07 mmol), 1*H*-imidazole-2-carboxylic acid (11 mg, 0.10 mmol) in DMF (3 mL) was added PyBOP (52 mg, 0.10 mmol) and DIEA (49 mg, 0.38 mmol). The mixture was stirred at room temperature for 12 h. The reaction mixture was concentrated to give a crude residue. The residue was purified by prep-HPLC (column: Xtimate C18 100 x 30 mm x 10 um; mobile phase: [water (FA)-ACN]; B%: 15%-45%, 10 min). The title compound (13.1 mg, 34% yield over 2 steps) was obtained as a white solid. ^{1}H NMR (DMSO-d_6, 400MHz) δ_H = 8.65 (s, 1H), 8.45 (d, *J* = 6.8 Hz, 1H), 7.58 (s, 1H), 7.48 (s, 2H), 7.37 (s, 1H), 7.17-7.01 (m, 3H), 6.8 (t, *J* = 6.8 Hz, 1H), 4.75-3.65 (m, 3H), 3.13-2.75 (s, 3H), 2.00-1.65 (m, 2H). ESI-MS [M+H]+ calcd. for $C_{23}H_{20}Cl_2N_6O_2$: 483.1, found: 483.3.

***tert*-butyl ((*S*)-1-(((3*S*,4*S*)-4-(3-chloro-5-fluorophenyl)-1-(imidazo[1,5-a]pyridine-8-carbonyl)piperidin-3-yl)amino)-3-methyl-1-oxobutan-2-yl)carbamate**

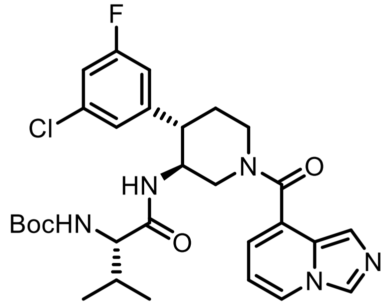

To a solution of ((3*S*,4*S*)-3-amino-4-(3-chloro-5-fluorophenyl)piperidin-1-yl)(imidazo[1,5-a]pyridin-8-yl)methanone (50 mg, 0.14 mmol) and Boc-L-Val-OH (30 mg, 0.14 mmol) in DMF (1 mL) was added PyBOP (91 mg, 0.18 mmol) and DIEA (52 mg, 0.40 mmol) at 0°C. The mixture was stirred at room temperature for 1 h. The mixture was diluted with EtOAc (3 mL) and washed with saturated aqueous LiCl (1 mL x3). The combined organic phase was dried over anhydrous Na_2SO_4, filtered and concentrated. The residue was purified by silica gel flash chromatography (0-100% EtOAc in PE). The title compound (60 mg, 63% yield) was obtained as a yellow solid. ^{1}H NMR ($CDCl_3$, 400MHz) δ_H = 8.68-7.64 (m, 1H), 7.59-7.26 (m, 2H), 7.25-6.63 (m, 5H), 5.00-4.61 (m, 1H), 4.17-4.00 (m, 1H), 3.83-3.55 (m, 1H), 3.21-2.67 (m, 2H), 2.18-1.94 (m, 3H), 1.93-1.79 (m, 2H), 1.48-1.29 (m, 9H), 0.90-0.41 (m, 6H).

(*S*)-2-amino-*N*-((3*S*,4*S*)-4-(3-chloro-5-fluorophenyl)-1-(imidazo[1,5-a]pyridine-8-carbonyl)piperidin-3-yl)-3-methylbutanamide

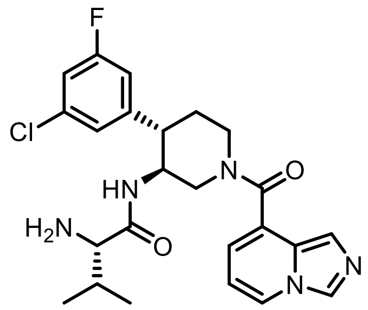

To a solution of *tert*-butyl ((*S*)-1-(((3*S*,4*S*)-4-(3-chloro-5-fluorophenyl)-1-(imidazo[1,5-a]pyridine-8-carbonyl)piperidin-3-yl)amino)-3-methyl-1-oxobutan-2-yl)carbamate (60 mg, 0.11 mmol) in dioxane (1 mL) was added HCl/dioxane (4 M, 500 uL) at room temperature. The mixture was stirred at 25°C for 1 h. Then the mixture was concentrated to provide the crude intermediate (50 mg, crude, HCl) as a yellow solid. ESI-MS $[M+H]^+$ calcd. for $C_{24}H_{27}ClFN_5O_2$: 472.2; found: 472.2.

(*S*)-*N*-((3*S*,4*S*)-4-(3-chloro-5-fluorophenyl)-1-(imidazo[1,5-a]pyridine-8-carbonyl)piperidin-3-yl)-3-methyl-2-(2,2,2-trifluoroacetamido)butanamide (27)

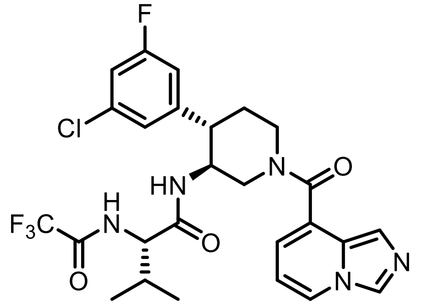

To a solution of (*S*)-2-amino-*N*-((3*S*,4*S*)-4-(3-chloro-5-fluorophenyl)-1-(imidazo[1,5-a]pyridine-8-carbonyl)piperidin-3-yl)-3-methylbutanamide (50 mg, 0.11 mmol) in toluene (2 mL) was added TEA (33 mg, 0.32 mmol) and ethyl 2,2,2-trifluoroacetate (31 mg, 0.22 mmol). The reaction mixture was stirred at 130 °C for 3 h. The mixture was filtered and concentrated to a crude residue. The product was purified by prep-HPLC (column: Welch Xtimate C18 150 × 30 mm × 5 µm; mobile phase: [water (NH_4HCO_3)-ACN]; B%: 27%-68%, 12min). The title compound (16.4 mg, 26% yield for 2 steps) was obtained as a light yellow solid. ^1H NMR (DMSO-d_6, 400MHz) δ_H= 9.54-9.20 (m, 1H), 8.45 (s, 1H), 8.39 (d, J = 7.2 Hz, 1H), 8.33-8.02 (m, 1H), 7.40 (s, 1H), 7.23 (s, 1H), 7.18-7.11 (m, 2H), 6.95 (d, J = 6.4 Hz, 1H), 6.71 (t, J = 6.8 Hz, 1H), 4.81-4.40 (m, 1H), 4.21-4.01 (m, 1H), 3.84 (d, J = 3.6 Hz, 1H), 3.21-2.69 (m, 4H), 1.94-1.65 (m, 3H), 0.88-0.61 (m, 6H). ESI-MS [M+H]+ calcd. for $C_{26}H_{26}ClF_4N_5O_3$: 568.2; found: 568.4.

***tert*-butyl ((*R*)-1-(((3*S*,4*S*)-4-(3-chloro-5-fluorophenyl)-1-(imidazo[1,5-a]pyridine-8-carbonyl)piperidin-3-yl)amino)-3-methyl-1-oxobutan-2-yl)carbamate**

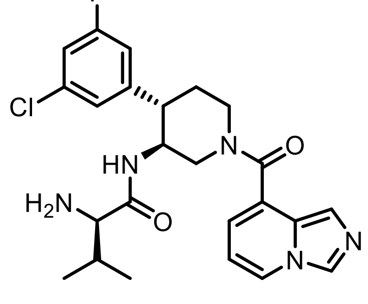

To a mixture of ((3*S*,4*S*)-3-amino-4-(3-chloro-5-fluorophenyl)piperidin-1-yl)(imidazo[1,5-a]pyridin-8-yl)methanone (50 mg, 0.13 mmol) and Boc-*D*-Val-OH (29 mg, 0.13 mmol) in DMF (2 mL) was added DIEA (87 mg, 0.67 mmol) and PyBOP (105 mg, 0.20 mmol). The reaction mixture was stirred at room temperature for 12 h, then the reaction mixture was diluted with EtOAc (10 mL) and washed with brine (5 mL x 3). The combined organic phases were dried over Na_2SO_4 and concentrated to provide a crude residue. The product was purified by silica gel flash chromatography (10% MeOH in DCM). The titled compound (80 mg, 92% yield) was obtained as a yellow oil. ESI-MS [M+H]+ calcd. for $C_{29}H_{35}ClFN_5O_4$: 572.2, found: 572.7.

(*R*)-*N*-((3*S*,4*S*)-4-(3-chloro-5-fluorophenyl)-1-(imidazo[1,5-a]pyridine-8-carbonyl)piperidin-3-yl)-3-methyl-2-(2,2,2-trifluoroacetamido)butanamide (28)

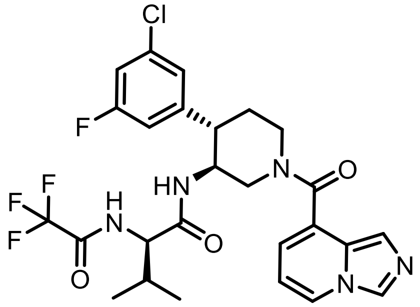

To a mixture of *tert*-butyl ((*R*)-1-(((3S,4S)-4-(3-chloro-5-fluorophenyl)-1-(imidazo[1,5-a]pyridine-8-carbonyl)piperidin-3-yl)amino)-3-methyl-1-oxobutan-2-yl)carbamate (80 mg, 0.14 mmol) in DCM (2 mL) was added HCl/dioxane (4 M, 1 mL). The reaction mixture was stirred at room temperature for 1 h. The reaction was concentrated directly to provide the crude deprotected intermediate (90 mg, crude) as a white solid, which was used in the next step without purification.

To a mixture of the crude deprotected intermediate (90 mg, crude) and ethyl 2,2,2-trifluoroacetate (54 mg, 0.38 mmol) in toluene (2 mL) was added TEA (58 mg, 0.57 mmol). The reaction mixture was stirred at 130 °C for 4 h. The reaction mixture was concentrated directly to give crude product. The crude product was purified by prep-HPLC (column: Phenomenex C18 75 x 30 mm x 3 um; mobile phase: [water (NH_3H_2O+NH_4HCO_3)-ACN]; B%: 30%-60%, 14 min). The title compound (16.4 mg, 15.0% yield over 2 steps) was obtained as a yellow solid. ^1H NMR (DMSO-d_6, 400MHz) δ_H = 9.43 (s, 1H), 8.53-8.35 (m, 2H), 8.33-8.13 (m, 1H), 7.42 (s, 1H), 7.36-7.18 (m, 3H), 6.95 (d, *J* = 14.8 Hz, 1H), 6.79-6.62 (m, 1H), 4.74-4.52 (m, 1H), 4.20-3.79 (m, 2H), 3.65-3.42 (m, 1H), 3.19-2.64 (m, 3H), 1.98-1.62 (m, 3H), 0.55 (s, 3H), 0.44-0.23 (m, 3H). ESI-MS [M+H]+ calcd. for $C_{26}H_{26}ClF_4N_5O_3$: 568.2, found: 568.4.

((*trans*)-4-(3-chloro-5-fluorophenyl)-3-nitropiperidin-1-yl)(1,6-naphthyridin-8-yl)methanone

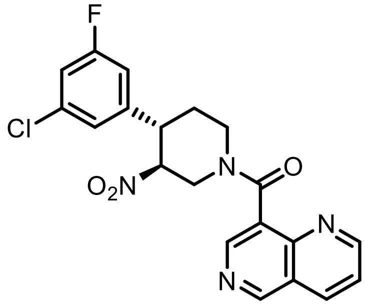

To a solution of (*trans*)-4-(3-chloro-5-fluorophenyl)-3-nitropiperidine (1.50 g, 5.80 mmol) in DMF (20 mL) was added 1,6-naphthyridine-8-carboxylic acid (605 mg, 3.48 mmol), DIEA (2.25 g, 17.4 mmol) and PyBOP (3.62 g, 6.96 mmol). The mixture was stirred at room temperature for 5 h. Water (50 mL) was

added to the reaction mixture and the resulting mixture was extracted with EtOAc (100 mL x 3). The combined organic extracts were washed with brine (100 mL x 5) dried over anhydrous Na_2SO_4, and concentrated to a crude residue. The product was purified by silica gel flash chromatography (90% EtOAc in PE). The title compound (1.10 g, 35% yield) was obtained as a brown oil. ESI-MS [M+H]+ calcd. for $C_{20}H_{16}ClFN_4O_3$: 415.1; found: 415.1

((*trans*)-3-amino-4-(3-chloro-5-fluorophenyl)piperidin-1-yl)(1,6-naphthyridin-8-yl)methanone

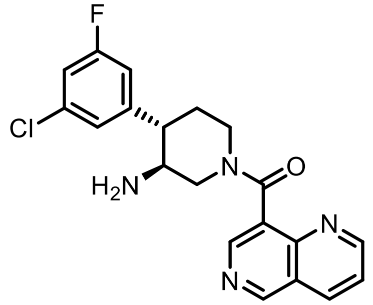

To a solution of ((*trans*)-4-(3-chloro-5-fluorophenyl)-3-nitropiperidin-1-yl)(1,6-naphthyridin-8-yl)methanone (0.90 g, 2.17 mmol) in EtOH (10 mL) and H_2O (10 mL) was added Fe (363 mg, 6.51 mmol) and NH_4Cl (348 mg, 6.51 mmol). The mixture was stirred at 100 °C for 3 h, then the reaction mixture was filtered through a celite pad washing with DCM : MeOH = 1:1 (300 mL). then the organic phase was partitioned and concentrated to dryness to give the crude intermediate (2.00 g, crude) as a yellow solid, which was used in the next step without purification.

***tert*-butyl ((3*S*,4*S*)-4-(3-chloro-5-fluorophenyl)-1-(1,6-naphthyridine-8-carbonyl)piperidin-3-yl)carbamate**

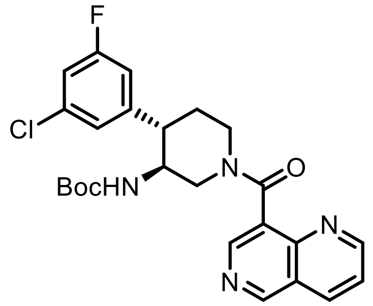

To a mixture of the crude intermediate (2.00 g, crude) in DCM (15 mL) was added Boc_2O (3.40 g, 15.6 mmol) and TEA (1.05 g, 10.4 mmol). The mixture was stirred at room temperature for 12 h, then the mixture was diluted with H_2O (30 mL) and extracted with DCM (30 mL x 3). The combined organic extracts were washed with brine (40 mL x 2), dried over anhydrous Na_2SO_4, and concentrated to give a crude residue. The product was first purified by silica gel flash chromatography (7% MeOH in DCM), then the product was further purified by prep-HPLC (column: Xtimate C18 150 x 40 mm x 10 um; mobile phase: [water (NH_4HCO_3)-ACN]; B%: 45%-85%, 10min). The title compound (300 mg, 14% yield over 2 steps) was obtained as a white solid.

***tert*-butyl ((*R*)-1-(((3*S*,4*S*)-4-(3-chloro-5-fluorophenyl)-1-(1,6-naphthyridine-8-carbonyl)piperidin-3-yl)amino)-3-methyl-1-oxobutan-2-yl)carbamate**

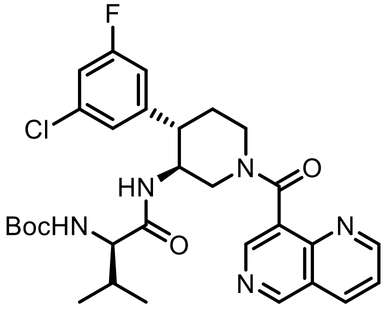

A solution of *tert*-butyl ((3*S*,4*S*)-4-(3-chloro-5-fluorophenyl)-1-(1,6-naphthyridine-8-carbonyl)piperidin-3-yl)carbamate (0.30 g, 0.61 mmol) in HCl/dioxane (4 M, 4 mL) was stirred at room temperature for 2 h. The reaction mixture was concentrated directly. To provide crude deprotected intermediate (270 mg, crude) as a white solid, which was used without purification.

To a solution of (2*R*)-2-(*tert*-butoxycarbonylamino)-3-methyl-butanoic acid (33 mg, 0.15 mmol) in DMF (2 mL) was added the crude deprotected intermediate (60 mg, crude), DIEA (80 mg, 0.62 mmol) and PyBOP (121 mg, 0.23 mmol). The mixture was stirred at room temperature for 3 h. The reaction mixture was diluted with H_2O (30 mL) and extracted with EtOAc (30 mL x 3). The combined organic extracts were washed with brine (60 mL x 5), dried over anhydrous Na_2SO_4, and concentrated. The residue was purified by silica gel flash chromatography (85% EtOAc in PE). The title compound (40 mg, 42% over 2 steps) was obtained as a white solid. ESI-MS [M+H]+ calcd. for $C_{25}H_{27}ClFN_5O_2$: 584.2; found: 584.3

(*R*)-*N*-((3*S*,4*S*)-4-(3-chloro-5-fluorophenyl)-1-(1,6-naphthyridine-8-carbonyl)piperidin-3-yl)-3-methyl-2-(2,2,2-trifluoroacetamido)butanamide (29)

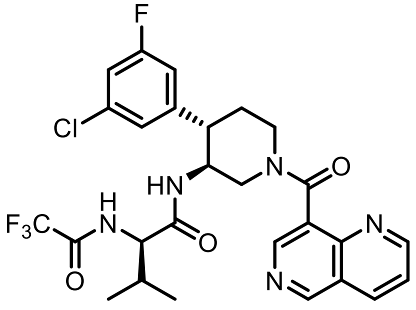

A mixture of *tert*-butyl ((*R*)-1-(((3*S*,4*S*)-4-(3-chloro-5-fluorophenyl)-1-(1,6-naphthyridine-8-carbonyl)piperidin-3-yl)amino)-3-methyl-1-oxobutan-2-yl)carbamate (40 mg, 0.06 mmol) in HCl/dioxane (4 M, 2 mL) was stirred at room temperature for 1 h. The reaction mixture was concentrated directly to give the crude deprotected product (30 mg, crude) as a yellow solid, which was used in the next step without purification.

To a mixture of the crude deprotected intermediate (30 mg, 0.06 mmol) in toluene (2 mL) was added ethyl 2,2,2-trifluoroacetate (88 mg, 0.61 mmol) and TEA (145 mg, 1.44 mmol). The mixture was degassed, purged with N_2, and stirred at 130 °C for 2 h in a microwave reactor. The reaction mixture was diluted with DMF (1 mL). The product was purified by prep-HPLC (column: Welch Xtimate C18 150 x 30mm x 5um; mobile phase: [water (NH_4HCO_3)-ACN]; B%: 30%-67%, 14min). The title compound (10.8 mg, 30% yield over 2 steps) was obtained as a white solid. ^{1}H NMR (DMSO-d_6, 400MHz) δ_H= 9.50 - 9.10 (m, 3H), 9.00-8.55 (m, 2H), 8.43-7.64 (m, 2H), 7.40-7.02 (m, 3H), 4.90-4.64 (m, 1H), 4.14-3.70

(m, 2H), 3.25-2.55 (m, 4H), 2.05-1.45 (m, 3H), 0.65-0.17 (m, 6H). ESI-MS [M+H]+ calcd. for $C_{27}H_{26}ClF_4N_5O_3$: 580.2; found: 580.2

Experimental procedures for analytical HPLC

(1) Analytical HPLC was performed with a photodiode array detector. The column was an Xbrige Shield RP-18 (50mm × 2.1 mm 5µm, Waters, America) with a temperature of 45 ℃ and a flow rate of 1.0 mL/min. Mobile phases A and B under a neutral condition were 0.02% NH_3H_2O in water and acetonitrile, respectively. The ratio of the mobile phase B was increased linearly from 0% to 60% over 6 min and 90% over the next 1 min. back to 0% acetonitrile (solvent B) in water and hold for 1.0 min.
(2) Analytical HPLC was performed with a photodiode array detector. The column was an Xbrige Shield RP-18 (50mm × 2.1 mm 5µm, Waters, America) with a temperature of 45 ℃ and a flow rate of 1.0 mL/min. Mobile phases A and B under a neutral condition were 0.02% NH_3H_2O in water and acetonitrile, respectively. The ratio of the mobile phase B was increased linearly from 10% to 80% over 6 min and 80% over the next 1 min. back to 10% acetonitrile (solvent B) in water and hold for 1.0 min.
(3) Analytical HPLC was performed with a photodiode array detector. The column was an Xtimate C18 (30mm × 2.1 mm 3µm, Welch, China) with a temperature of 45 ℃ and a flow rate of 1.0 mL/min. Mobile phases A and B under a neutral condition were 0.037% TFA in water and 0.018 % TFA in acetonitrile, respectively. The ratio of the mobile phase B was increased linearly from 0% to 60% over 6 min and 60% over the next 1 min. back to 0% acetonitrile (solvent B) in water and hold for 1.0 min.
(4) Analytical HPLC was performed with a photodiode array detector. The column was a Poroshell 120 EC-C18 (50mm × 3.0 mm 2.7µm, Agilent, America) with a temperature of 45 ℃ and a flow rate of 1.0 mL/min. Mobile phases A and B under a neutral condition were 0.037% TFA in water and 0.018 % TFA in acetonitrile, respectively. The ratio of the mobile phase B was increased linearly from 0% to 60% over 6 min and 60% over the next 1 min. back to 0% acetonitrile (solvent B) in water and hold for 1.0 min.
(5) Analytical HPLC was performed with a photodiode array detector. The column was a Poroshell 120 EC-C18 (50mm × 3.0 mm 2.7µm, Agilent, America) with a temperature of 45 ℃and a flow rate of 1.0 mL/min. Mobile phases A and B under a neutral condition were 0.037% TFA in water and 0.018 % TFA in acetonitrile, respectively. The ratio of the mobile phase B was increased linearly from 10% to 80% over 6 min and 80% over the next 1 min. back to 10% acetonitrile (solvent B) in water and hold for 1.0 min.
(6) Analytical HPLC was performed with a corona charged aerosol detector (CAD), a nanoquantity analyte detector (NQAD), or a photodiode array detector. The column was a Capcell Pak C18AQ (50 mm × 3.0 mm I.D., Shiseido, Japan) or Acquity UPLC BEH C18 (50 mm x 2.1 mm I.D., Waters, MA, USA) with a temperature of 50 °C and a flow rate of 0.5 mL/min. Mobile phases A and B under a neutral condition were a mixture of 5 mmol/L ammonium acetate and 5 mmol/L ammonium acetate in 98% acetonitrile, respectively. Mobile phases A and B under an acidic condition were a mixture of 0.2% formic acid in 10 mmol/L ammonium formate and 0.2% formic acid in acetonitrile, respectively. The ratio of the mobile phase B was increased linearly from 5 to 99% over 3.2 min and 99% over the next 0.4 min.

Compound Spectra

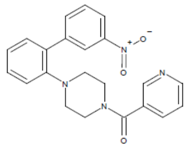

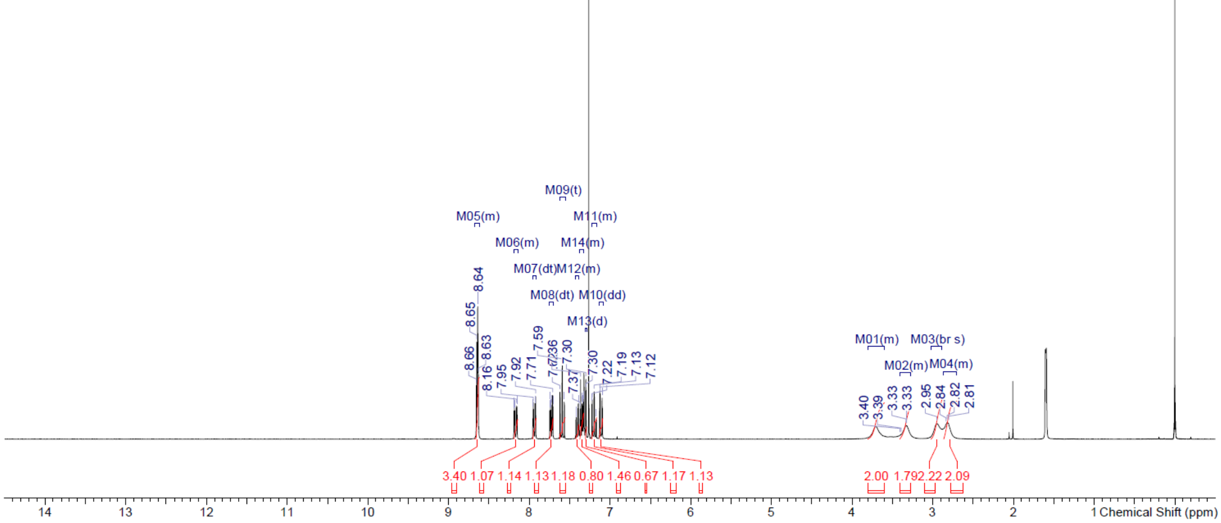

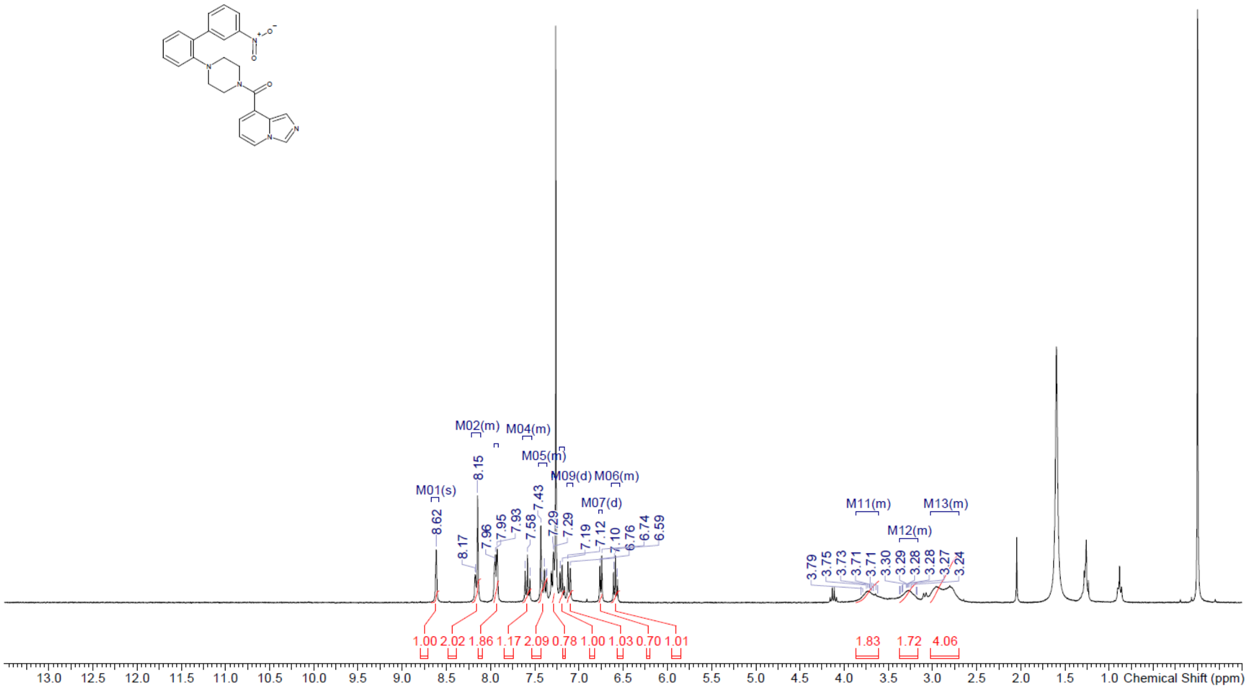
M01(s)
M02(m)
M04(m)
M05(m)
M09(d) M06(m)
M07(d)
M11(m)
M12(m)
M13(m)
Chemical Shift (ppm)

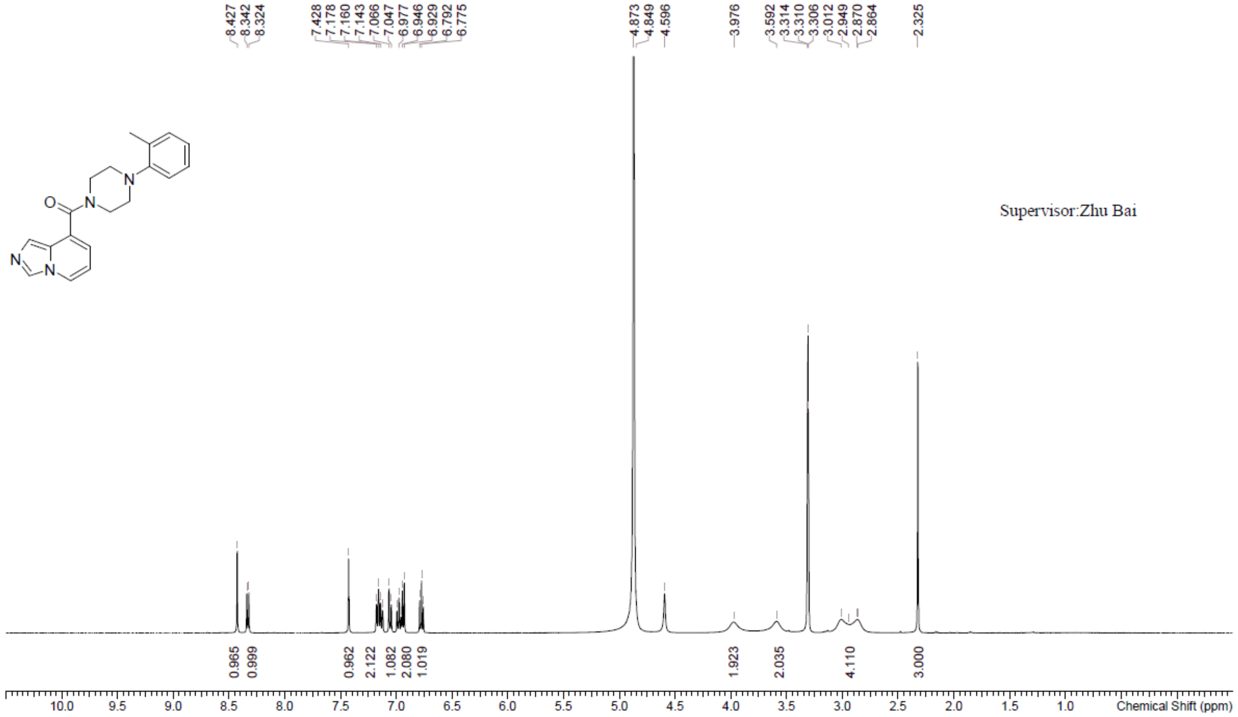
Supervisor:Zhu Bai
Chemical Shift (ppm)

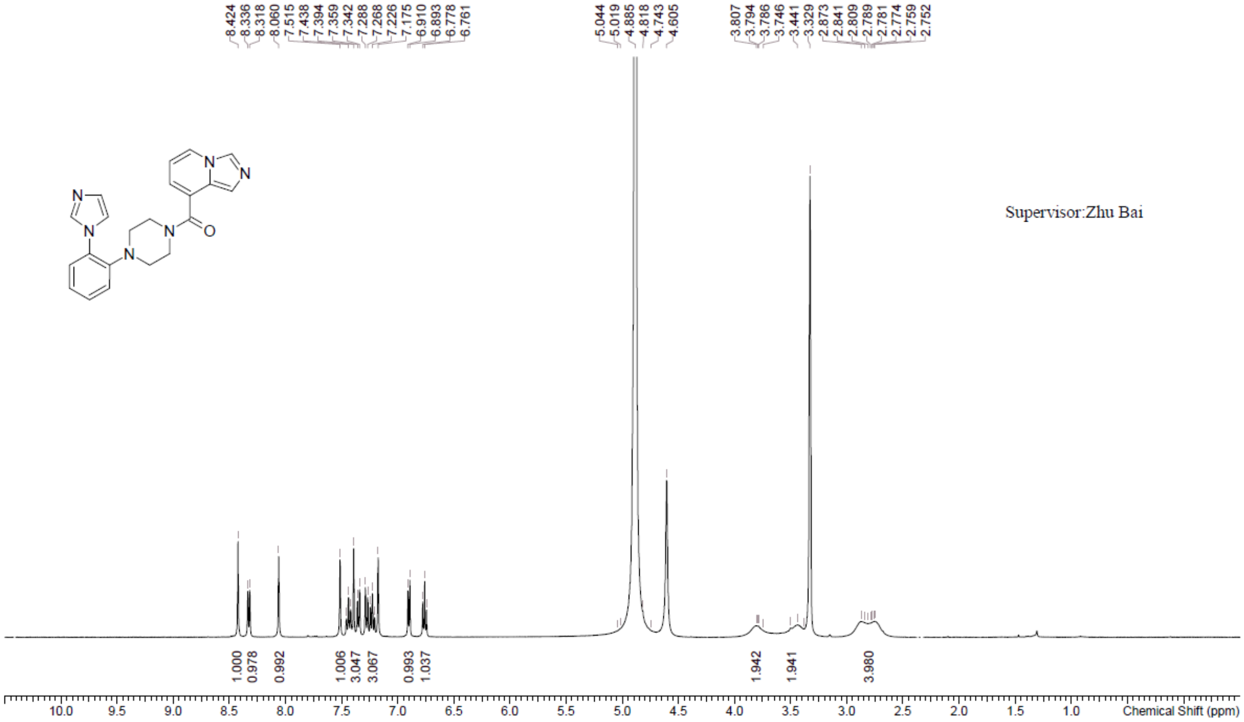
8.424 8.336 8.318 8.060 7.515 7.438 7.394 7.359 7.342 7.288 7.268 7.226 7.175 6.910 6.893 6.778 6.761
5.044 5.019 4.885 4.818 4.743 4.605
3.807 3.794 3.786 3.746 3.441 3.329 2.873 2.841 2.809 2.789 2.781 2.774 2.759 2.752
Supervisor:Zhu Bai
1.000 0.978 0.992 1.006 3.047 3.067 0.993 1.037 1.942 1.941 3.980
Chemical Shift (ppm)

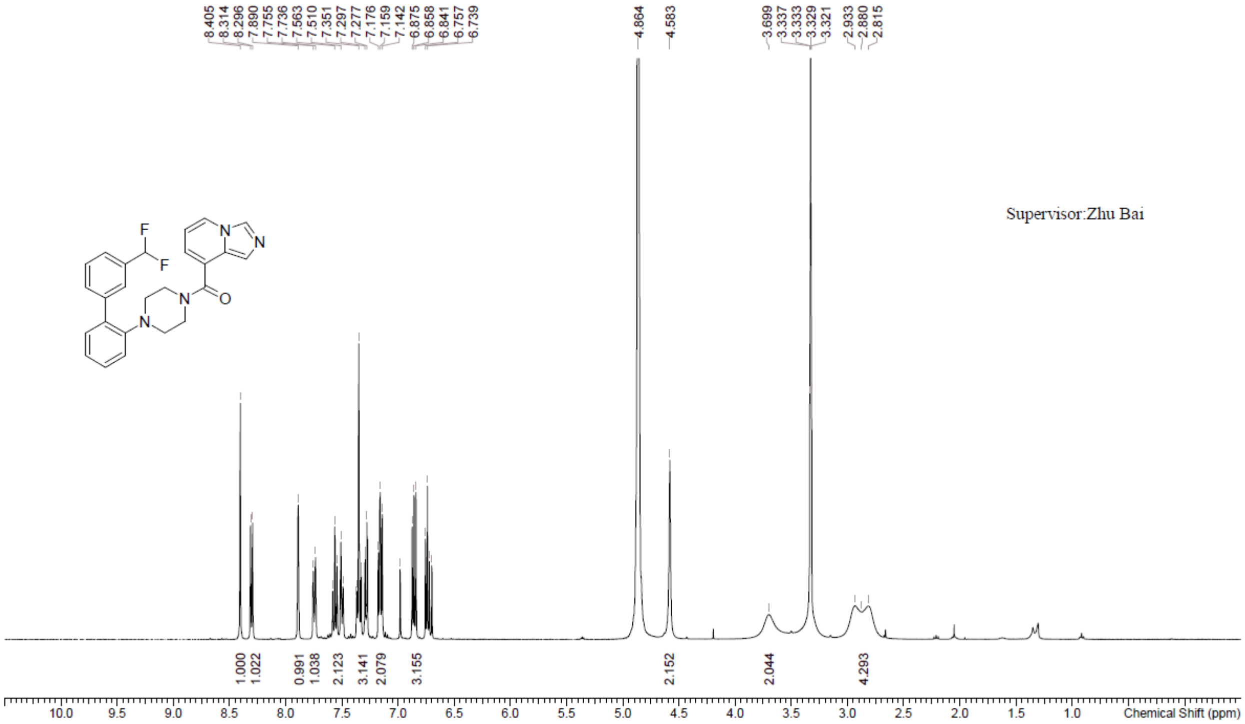
Supervisor:Zhu Bai
Chemical Shift (ppm)

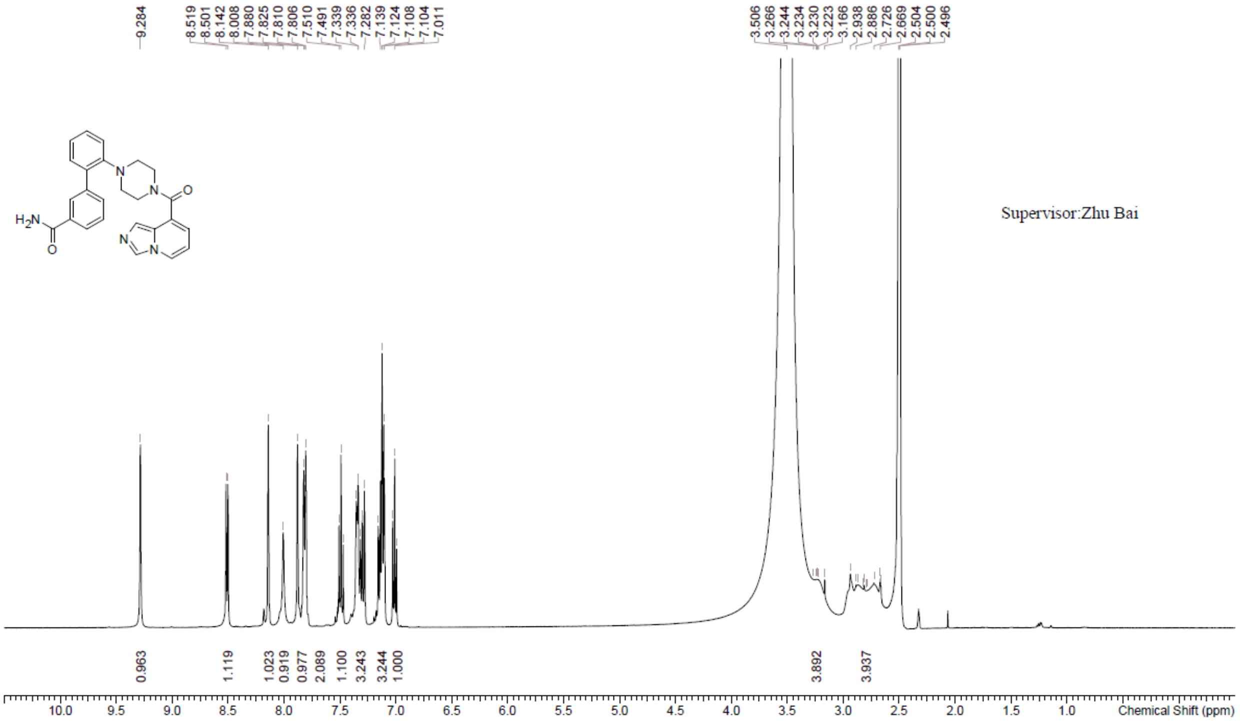
Supervisor:Zhu Bai
Chemical Shift (ppm)

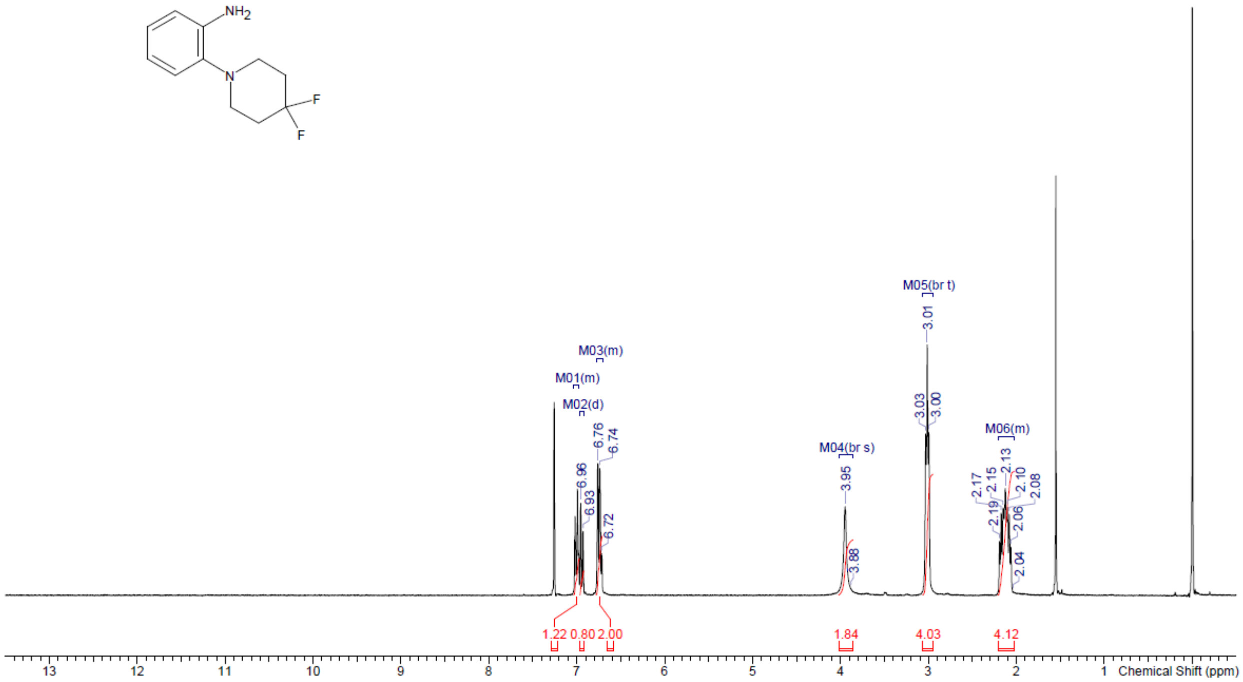
NH2
N
F
F
M05(br t)
M03(m)
M01(m)
M02(d)
M04(br s)
M06(m)
Chemical Shift (ppm)

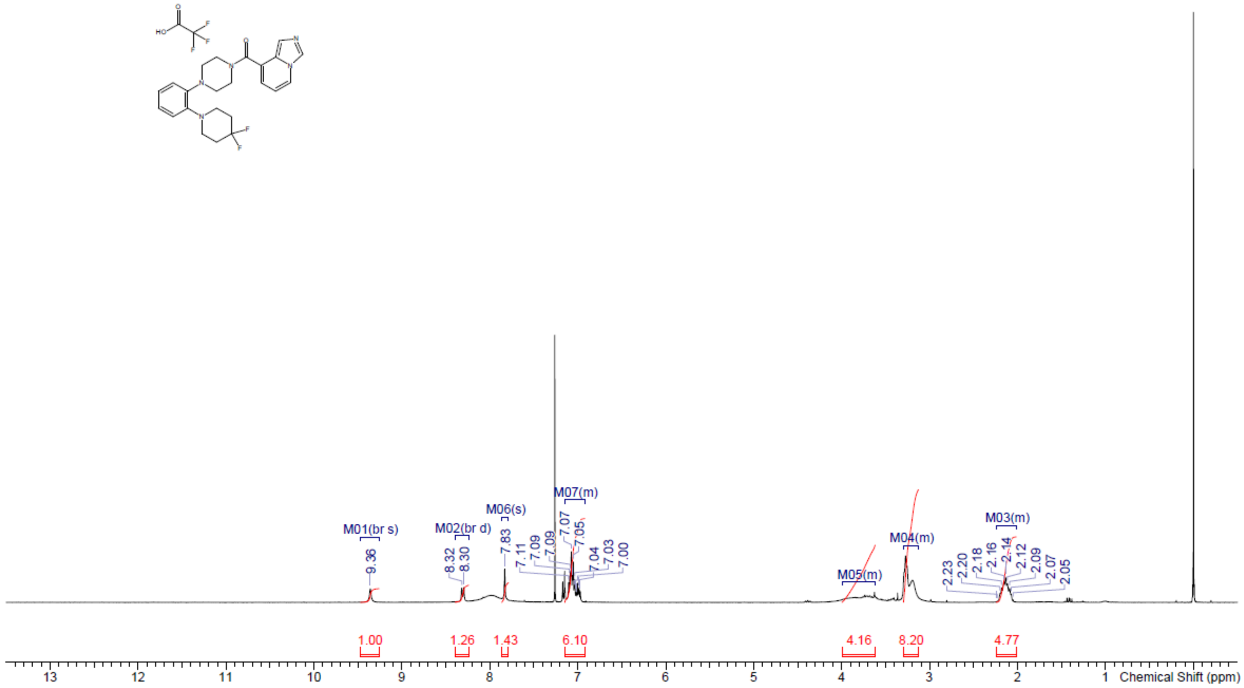
M01(br s)
M02(br d)
M06(s)
M07(m)
M04(m)
M05(m)
M03(m)
Chemical Shift (ppm)

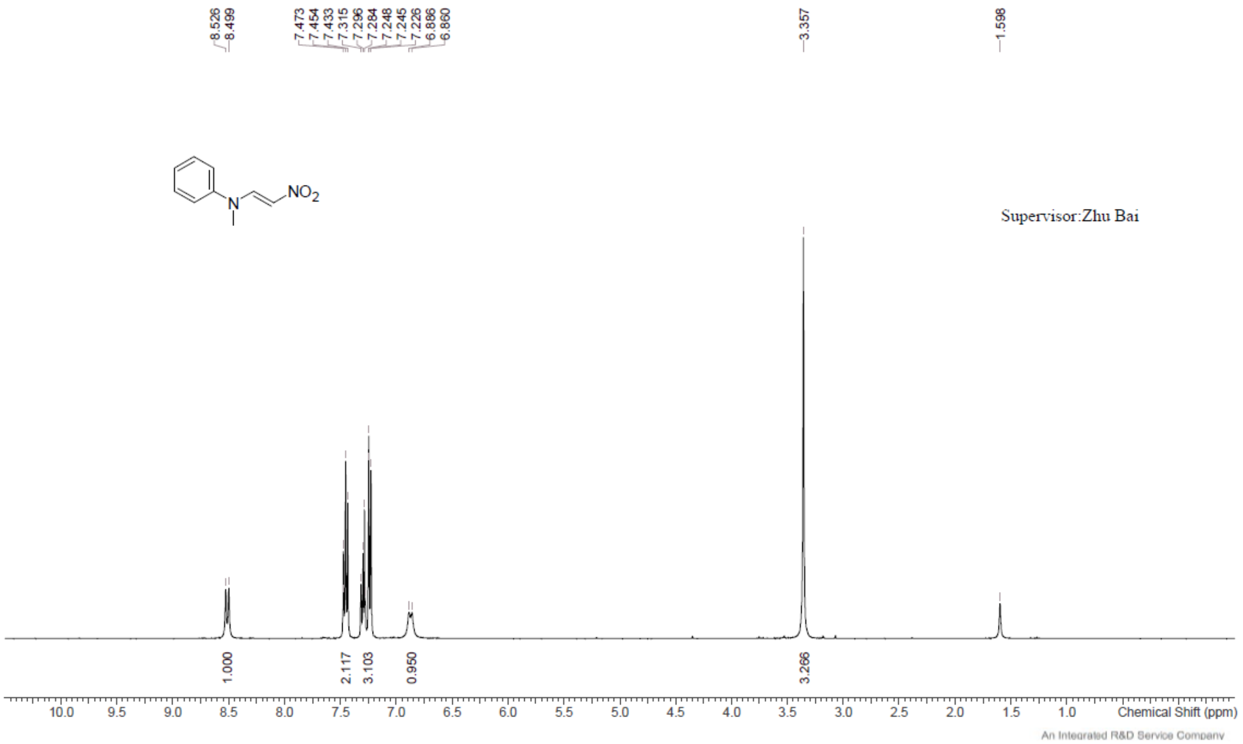
8.526
8.499
7.473
7.454
7.433
7.315
7.296
7.284
7.248
7.245
7.226
6.886
6.860
3.357
1.598
NO_2
N
Supervisor:Zhu Bai
1.000
2.117
3.103
0.950
3.266
10.0 9.5 9.0 8.5 8.0 7.5 7.0 6.5 6.0 5.5 5.0 4.5 4.0 3.5 3.0 2.5 2.0 1.5 1.0
Chemical Shift (ppm)
An Integrated R&D Service Company

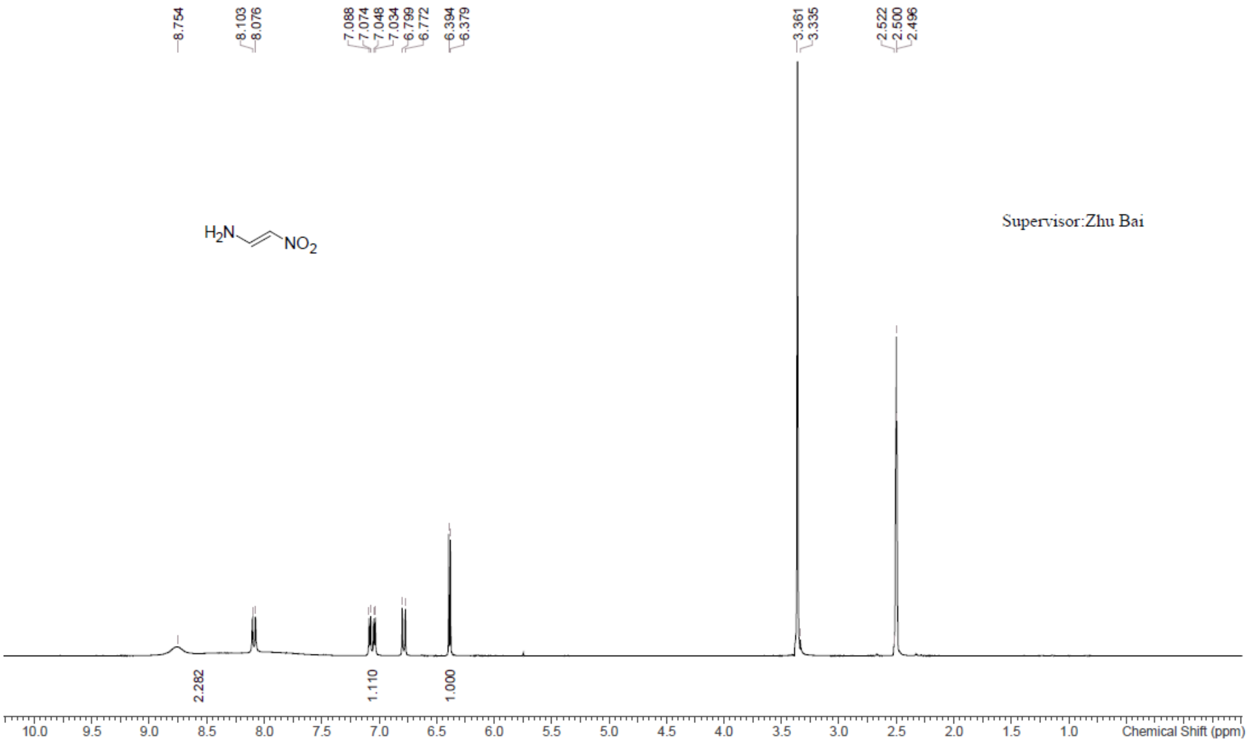
8.754
8.103
8.076
7.088
7.074
7.048
7.034
6.799
6.772
6.394
6.379
3.361
3.335
2.522
2.500
2.496
H_2N
NO_2
Supervisor:Zhu Bai
2.282
1.110
1.000
10.0 9.5 9.0 8.5 8.0 7.5 7.0 6.5 6.0 5.5 5.0 4.5 4.0 3.5 3.0 2.5 2.0 1.5 1.0
Chemical Shift (ppm)

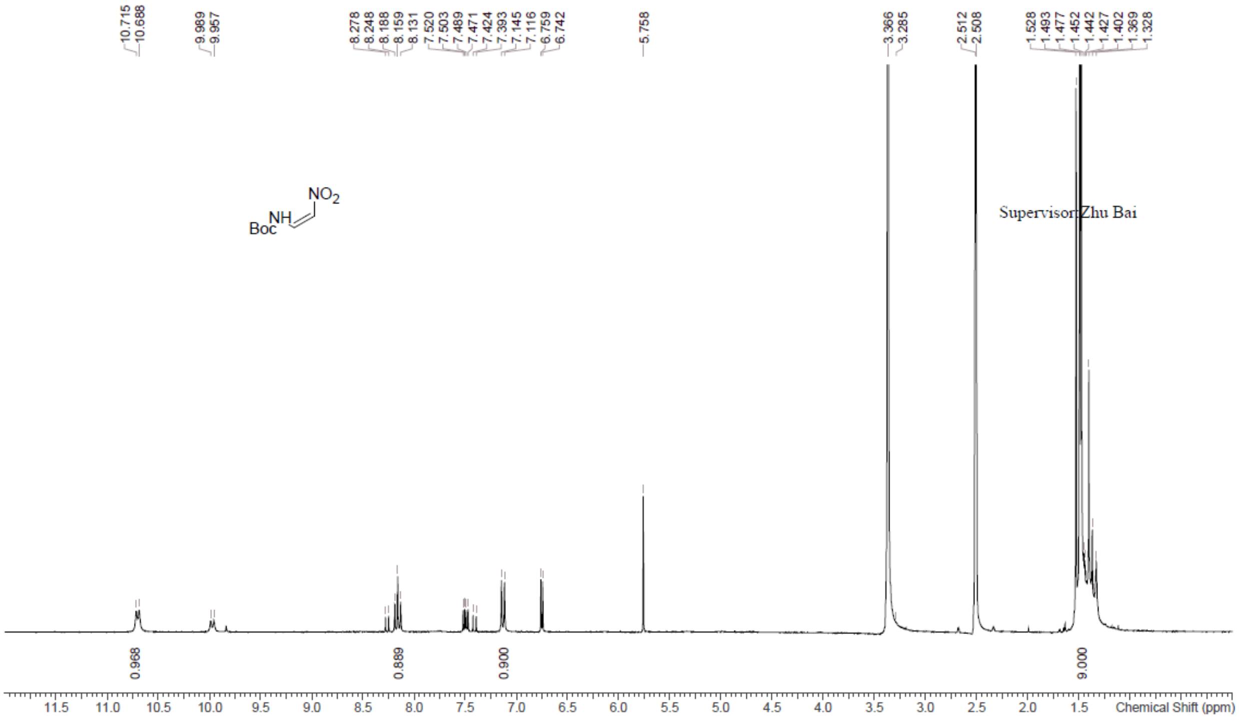

NO2
Boc NH
Supervisor:Zhu Bai
Chemical Shift (ppm)

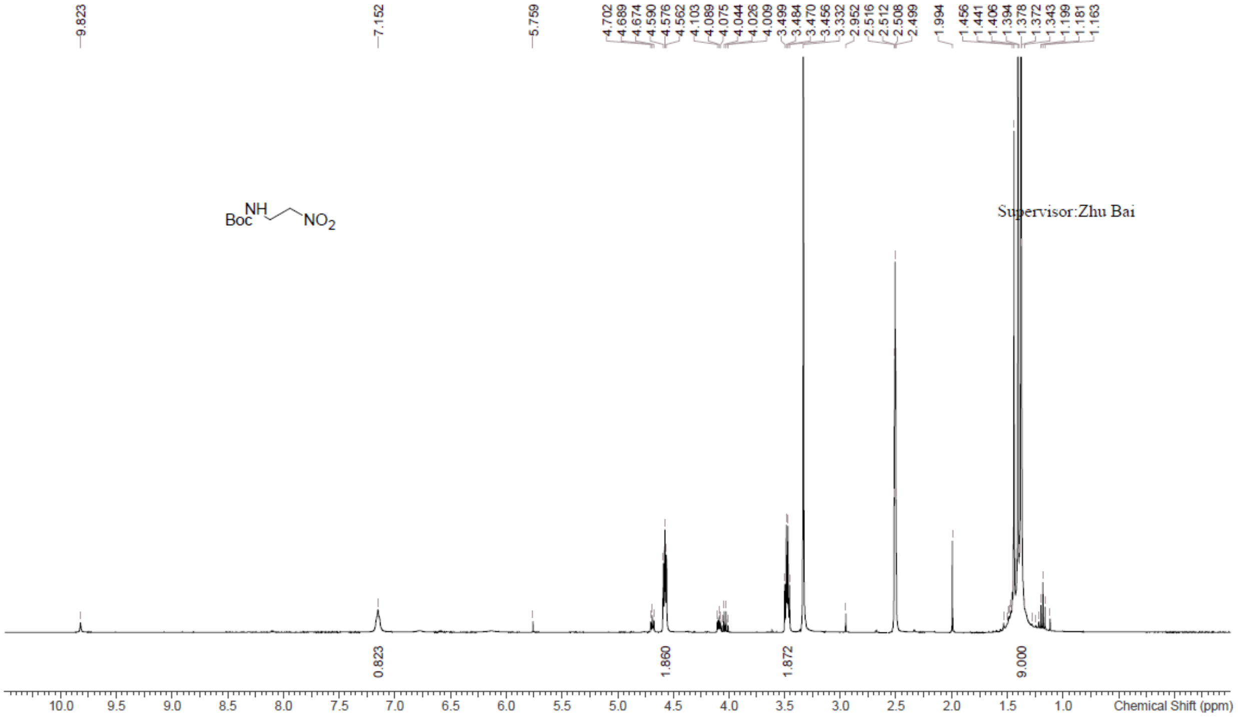

Boc NH NO2
Supervisor:Zhu Bai
Chemical Shift (ppm)

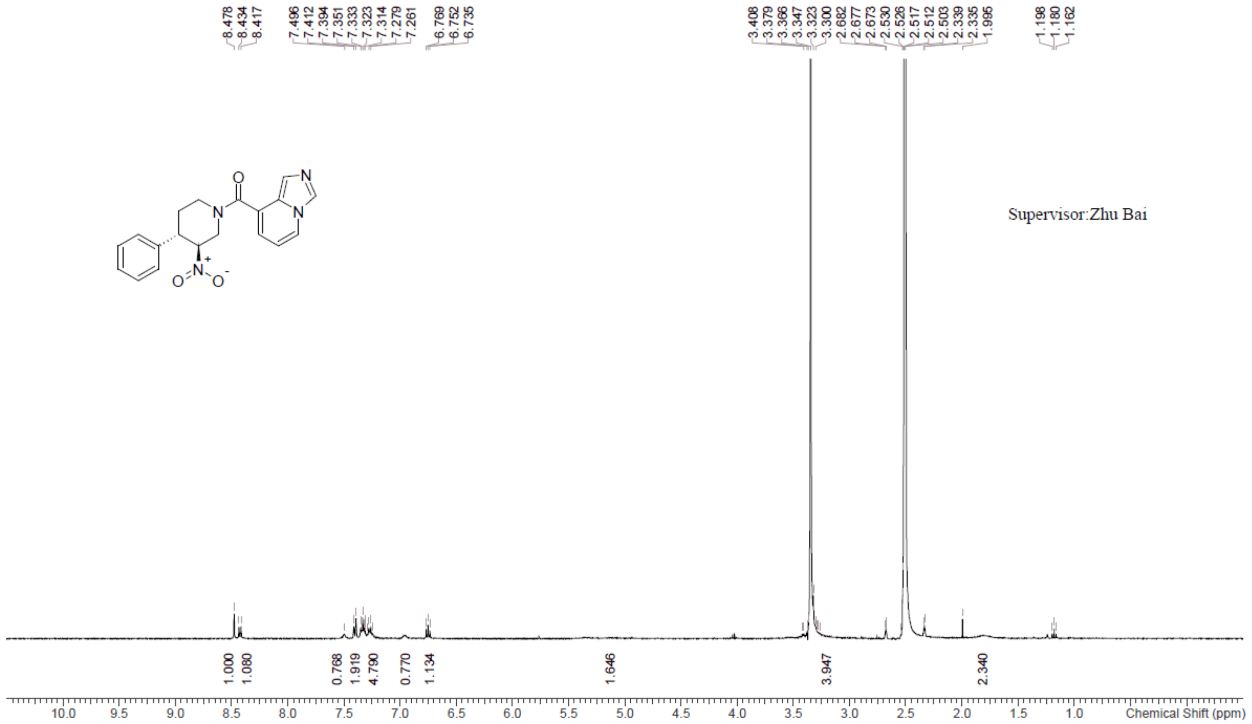
Supervisor:Zhu Bai
Chemical Shift (ppm)

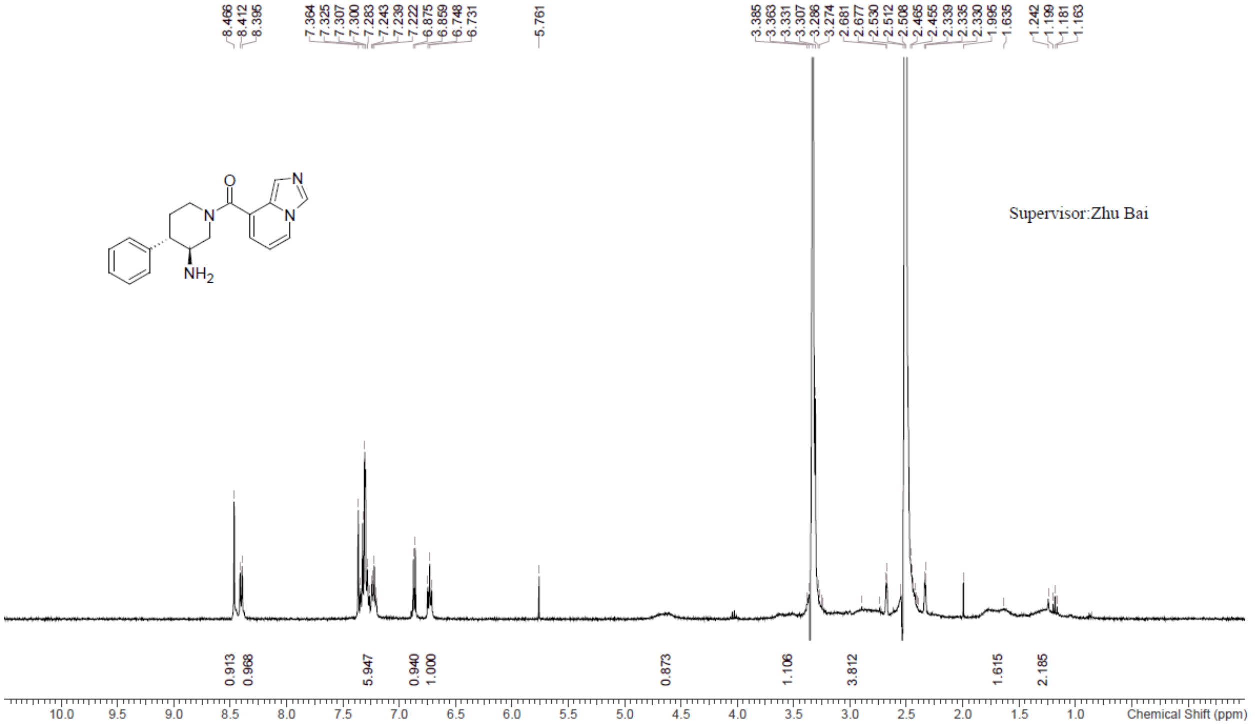
Supervisor:Zhu Bai
Chemical Shift (ppm)

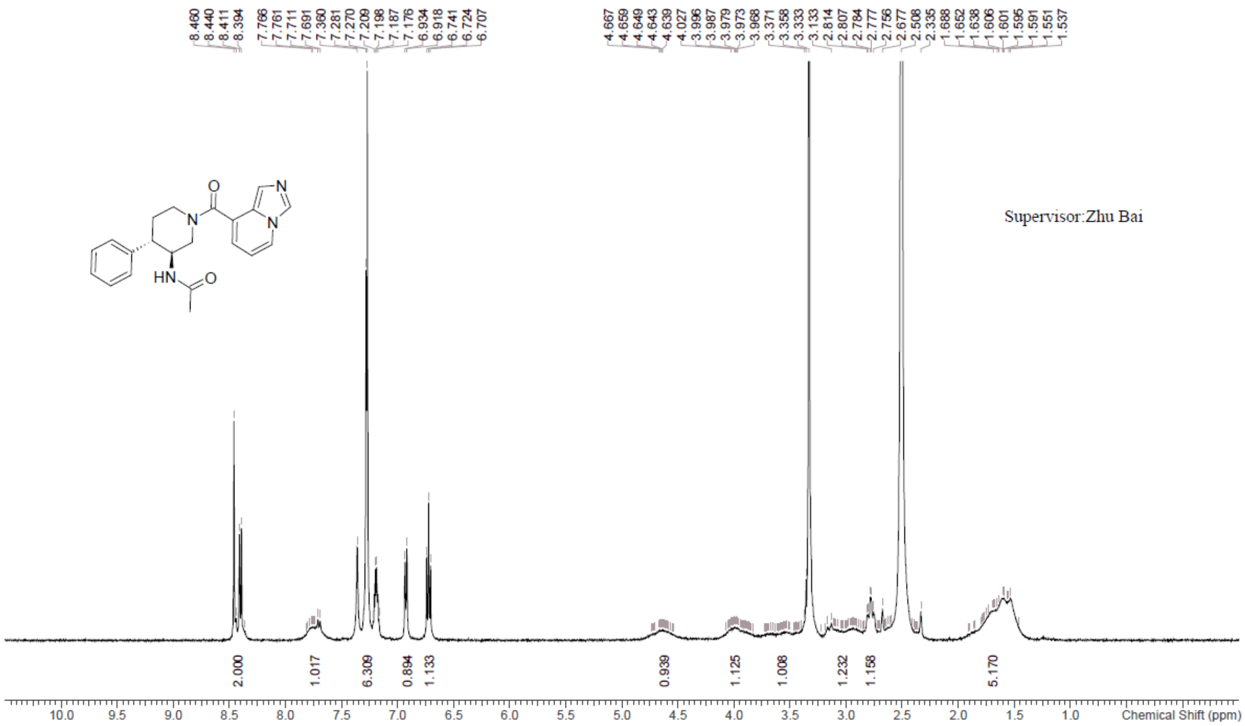
Supervisor:Zhu Bai
Chemical Shift (ppm)

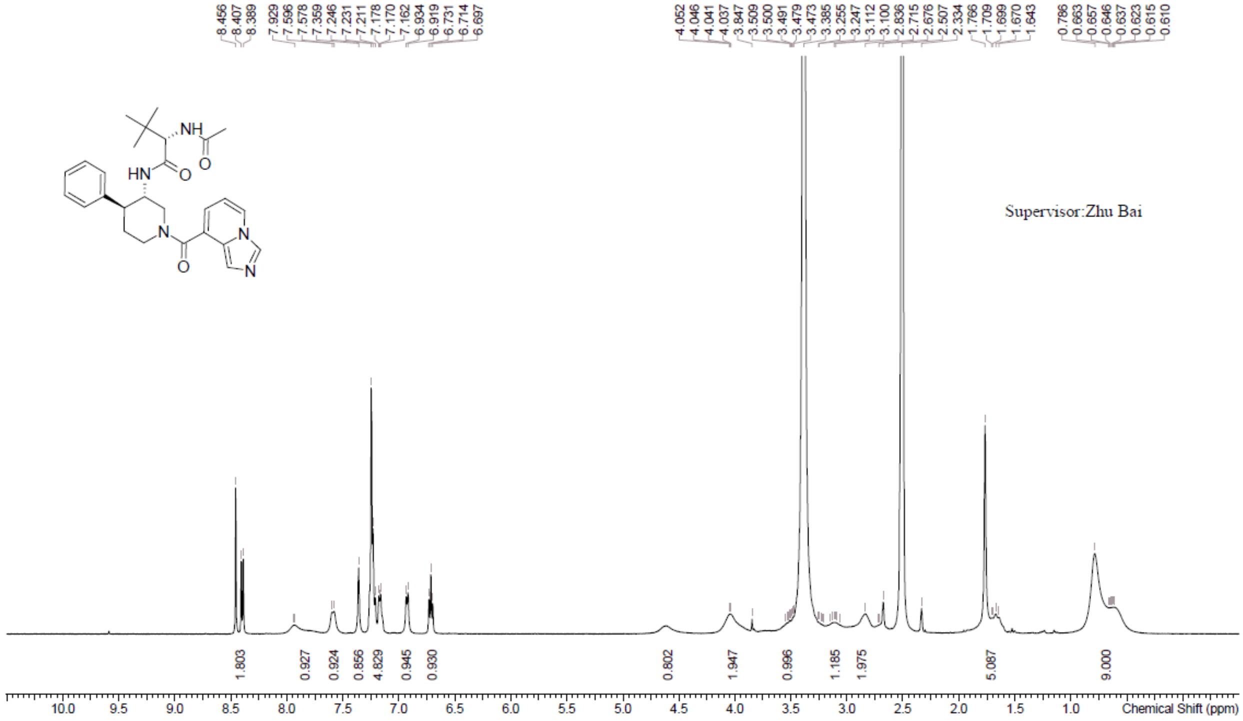
Supervisor:Zhu Bai
Chemical Shift (ppm)

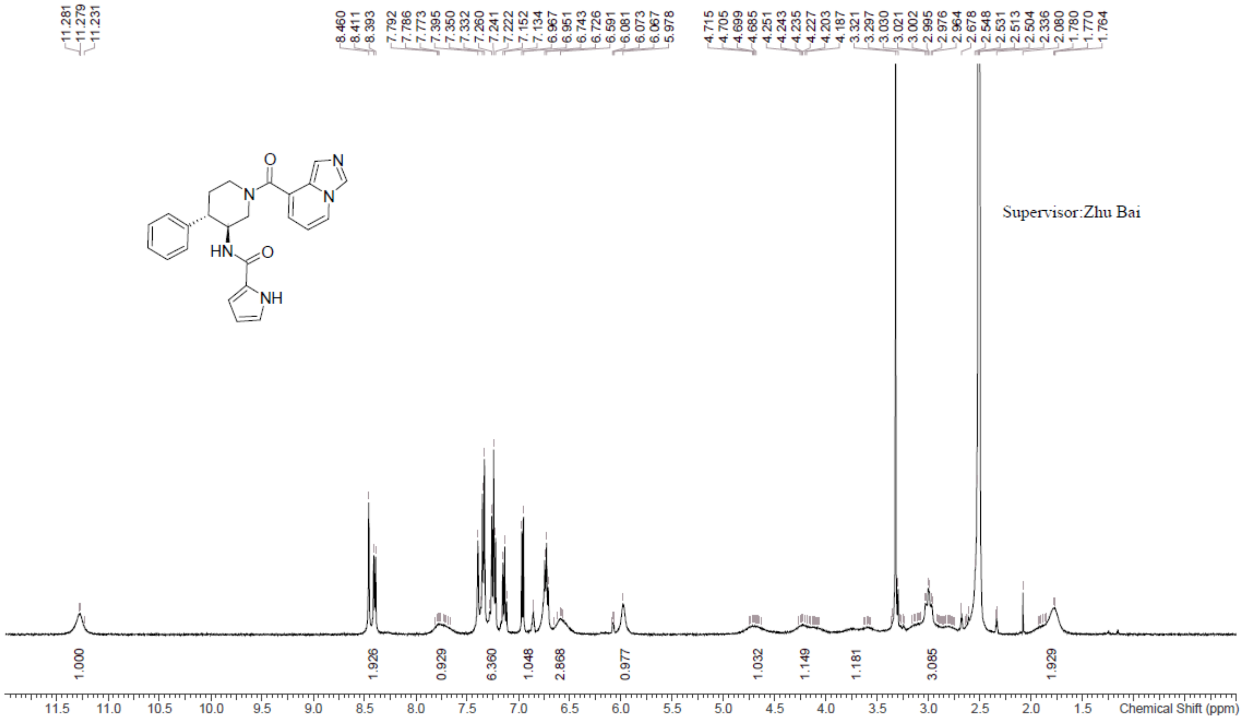
Supervisor:Zhu Bai
Chemical Shift (ppm)

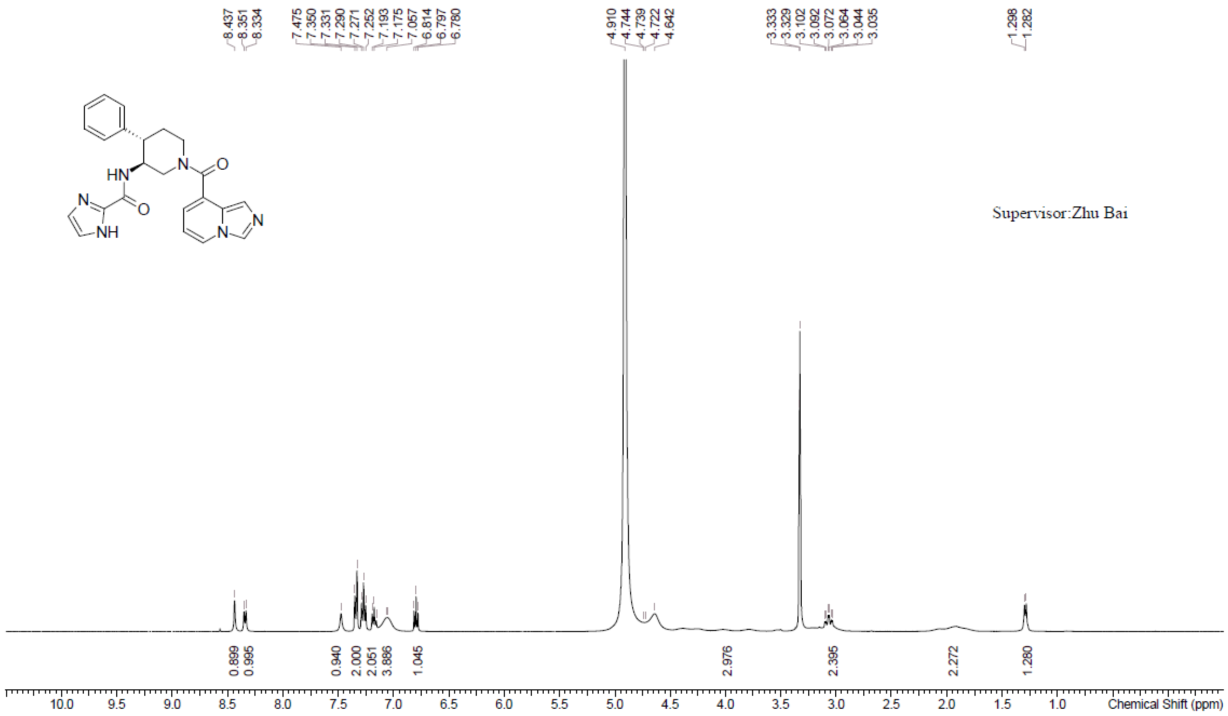
Supervisor:Zhu Bai
Chemical Shift (ppm)

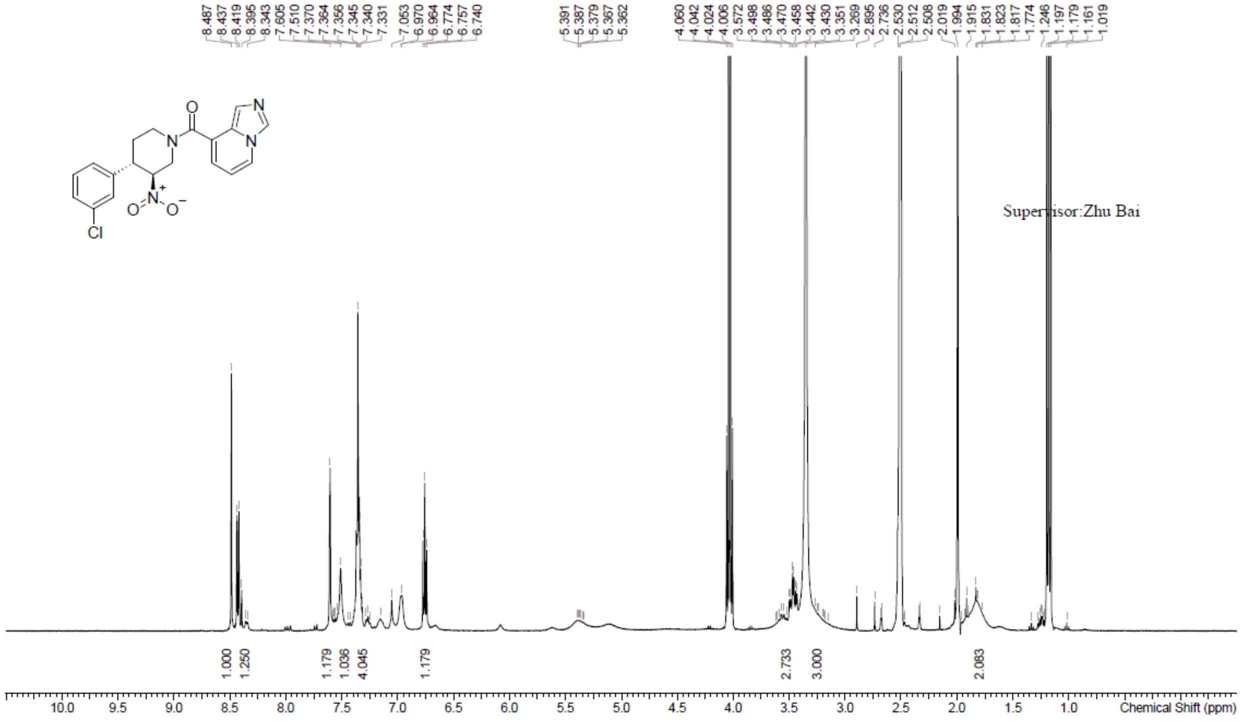
Supervisor:Zhu Bai
Chemical Shift (ppm)

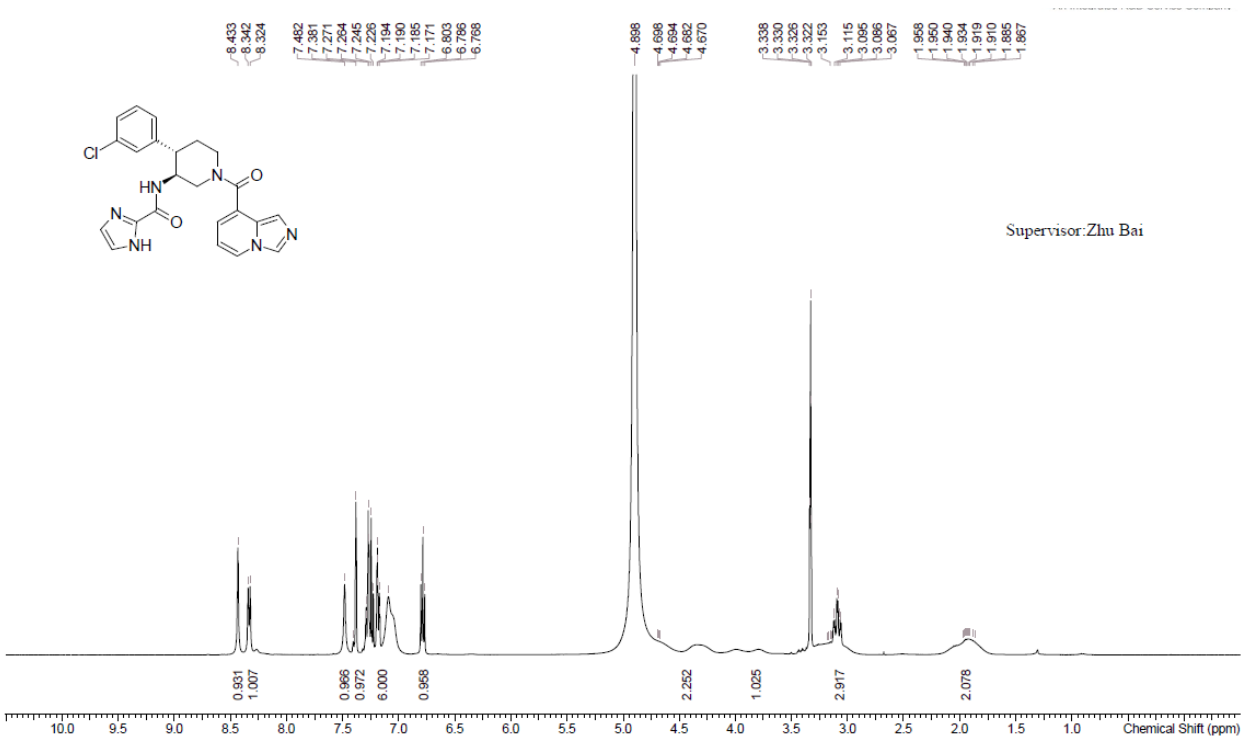
8.433
8.342
8.324
7.482
7.381
7.271
7.264
7.245
7.226
7.194
7.190
7.185
7.171
6.803
6.786
6.768
4.898
4.698
4.694
4.682
4.670
3.338
3.330
3.326
3.322
3.153
3.115
3.095
3.086
3.067
1.958
1.950
1.940
1.934
1.919
1.910
1.885
1.867
Cl
HN
N
O
N
O
NH
N
N
Supervisor:Zhu Bai
0.931
1.007
0.966
0.972
6.000
0.958
2.252
1.025
2.917
2.078
10.0
9.5
9.0
8.5
8.0
7.5
7.0
6.5
6.0
5.5
5.0
4.5
4.0
3.5
3.0
2.5
2.0
1.5
1.0
Chemical Shift (ppm)

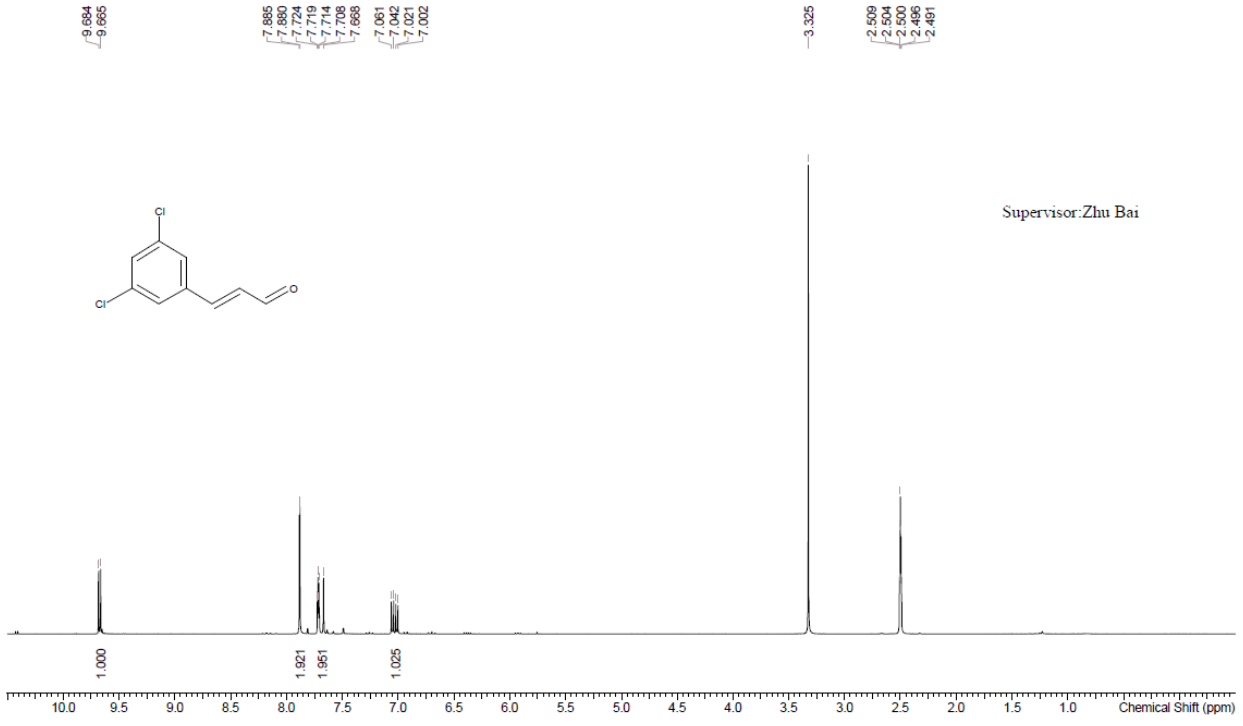
9.684
9.665
7.885
7.880
7.724
7.719
7.714
7.708
7.668
7.061
7.042
7.021
7.002
3.325
2.509
2.504
2.500
2.496
2.491
Cl
Cl
O
Supervisor:Zhu Bai
1.000
1.921
1.951
1.025
10.0
9.5
9.0
8.5
8.0
7.5
7.0
6.5
6.0
5.5
5.0
4.5
4.0
3.5
3.0
2.5
2.0
1.5
1.0
Chemical Shift (ppm)

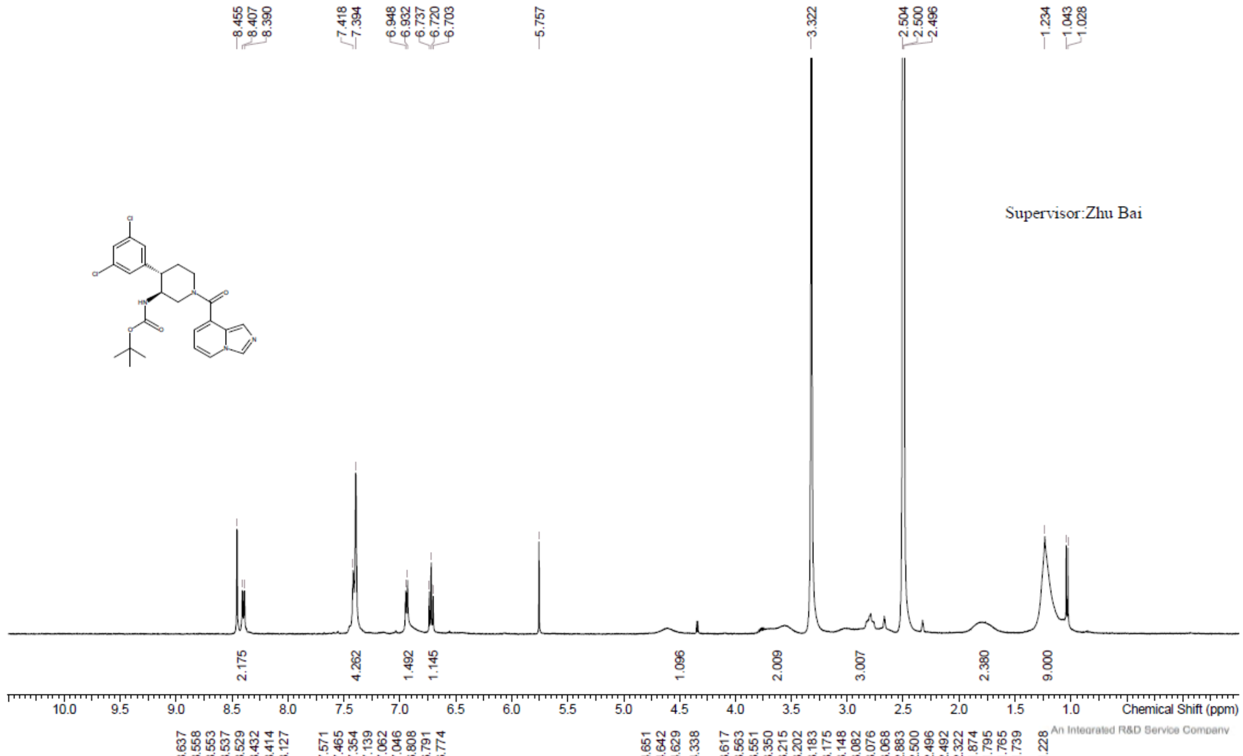
Supervisor:Zhu Bai
Chemical Shift (ppm)
An Integrated R&D Service Company

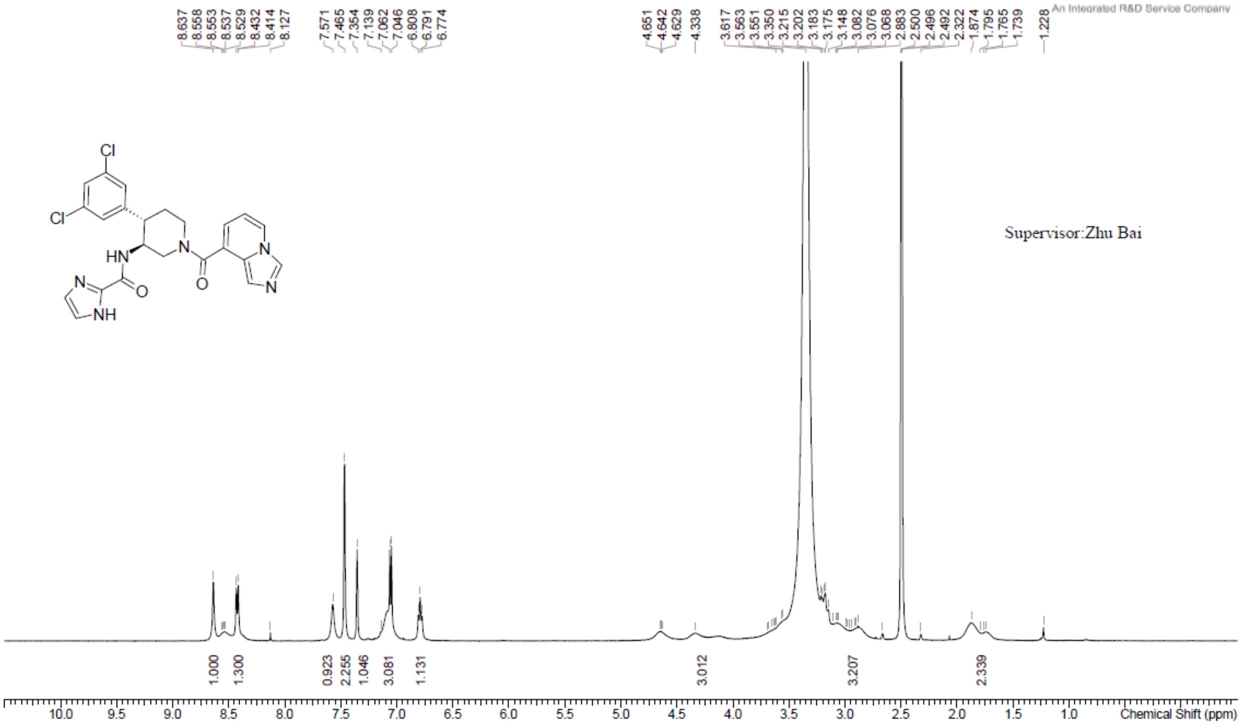
Supervisor:Zhu Bai
Chemical Shift (ppm)

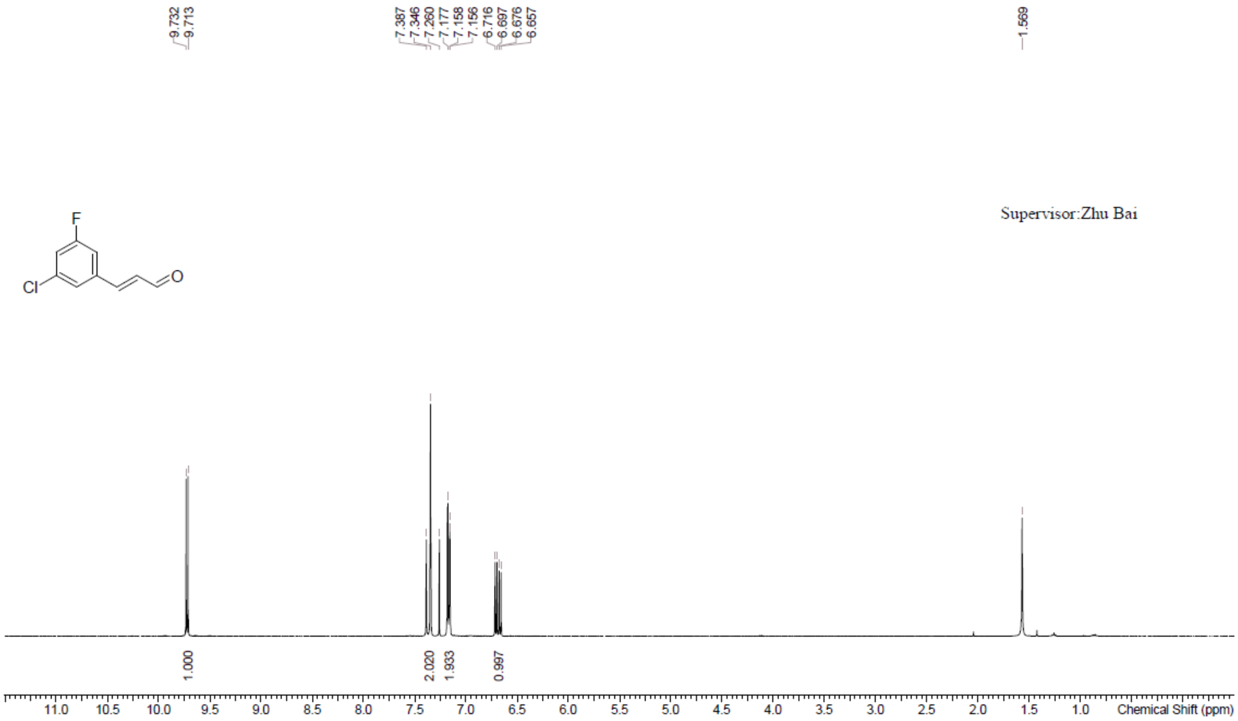
9.732
9.713
7.387
7.346
7.260
7.177
7.158
7.156
6.716
6.697
6.676
6.657
1.569
Supervisor:Zhu Bai
F
Cl
O
1.000
2.020
1.933
0.997
Chemical Shift (ppm)

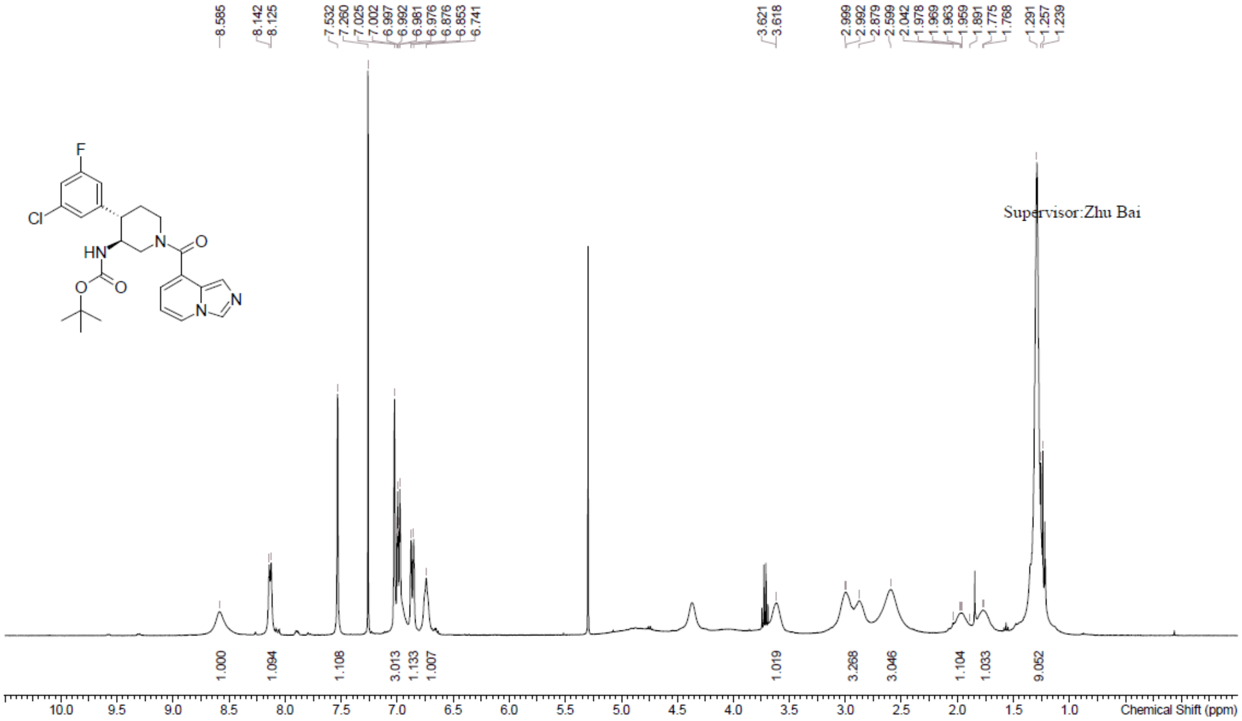
8.585
8.142
8.125
7.532
7.260
7.025
7.002
6.997
6.992
6.981
6.976
6.876
6.853
6.741
3.621
3.618
2.999
2.992
2.879
2.599
2.042
1.978
1.969
1.963
1.959
1.891
1.775
1.768
1.291
1.257
1.239
Supervisor:Zhu Bai
F
Cl
HN
N
O
N
1.000
1.094
1.108
3.013
1.133
1.007
1.019
3.268
3.046
1.104
1.033
9.052
Chemical Shift (ppm)

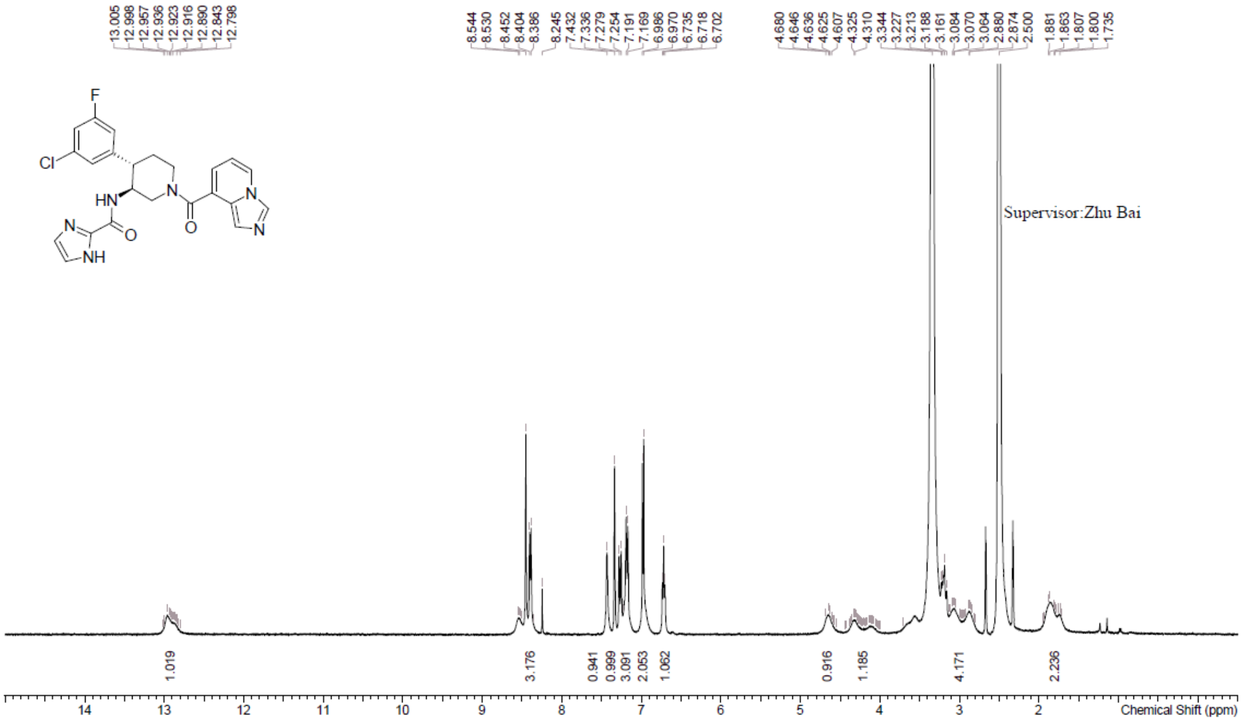
13.005
12.998
12.957
12.936
12.923
12.916
12.890
12.843
12.798
8.544
8.530
8.452
8.404
8.386
8.245
7.432
7.336
7.279
7.254
7.191
7.169
6.986
6.970
6.735
6.718
6.702
4.680
4.646
4.636
4.625
4.607
4.325
4.310
3.344
3.227
3.213
3.188
3.161
3.084
3.070
3.064
2.880
2.874
2.500
1.881
1.863
1.807
1.800
1.735
F
Cl
HN
N
O
N
N
NH
N
O
N
Supervisor:Zhu Bai
1.019
3.176
0.941
0.999
3.091
2.053
1.062
0.916
1.185
4.171
2.236
14
13
12
11
10
9
8
7
6
5
4
3
2
Chemical Shift (ppm)

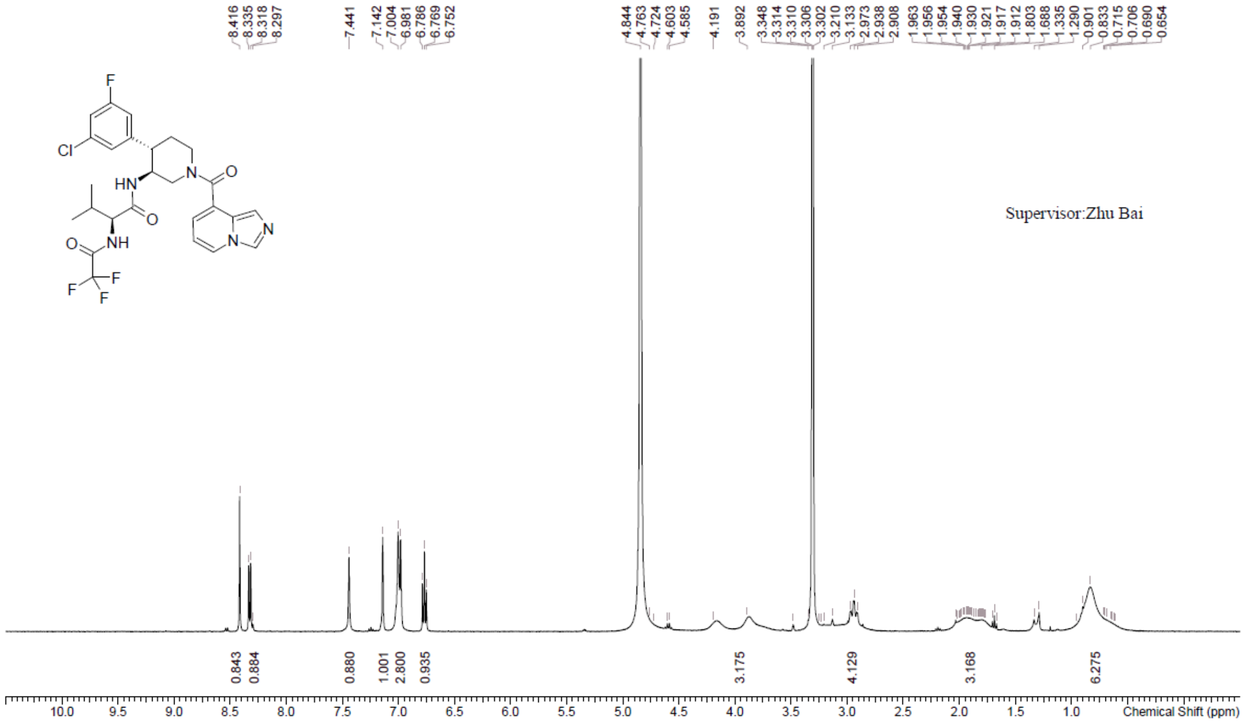
8.416
8.335
8.318
8.297
7.441
7.142
7.004
6.981
6.786
6.769
6.752
4.844
4.763
4.724
4.603
4.585
4.191
3.892
3.348
3.314
3.310
3.306
3.302
3.210
3.133
2.973
2.938
2.908
1.963
1.956
1.954
1.940
1.930
1.921
1.917
1.912
1.803
1.688
1.335
1.290
0.901
0.833
0.715
0.706
0.690
0.654
F
Cl
HN
N
O
O
NH
O
F
F
F
N
N
Supervisor:Zhu Bai
0.843
0.884
0.880
1.001
2.800
0.935
3.175
4.129
3.168
6.275
10.0
9.5
9.0
8.5
8.0
7.5
7.0
6.5
6.0
5.5
5.0
4.5
4.0
3.5
3.0
2.5
2.0
1.5
1.0
Chemical Shift (ppm)

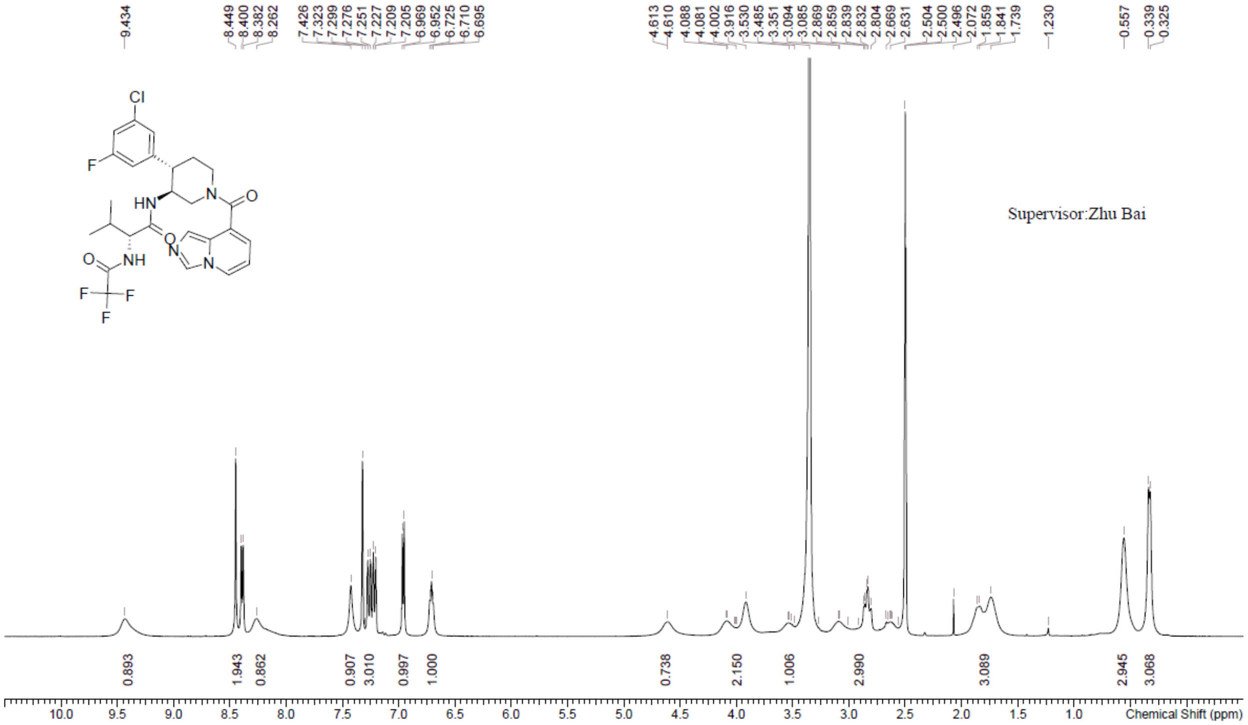
Supervisor:Zhu Bai
Chemical Shift (ppm)

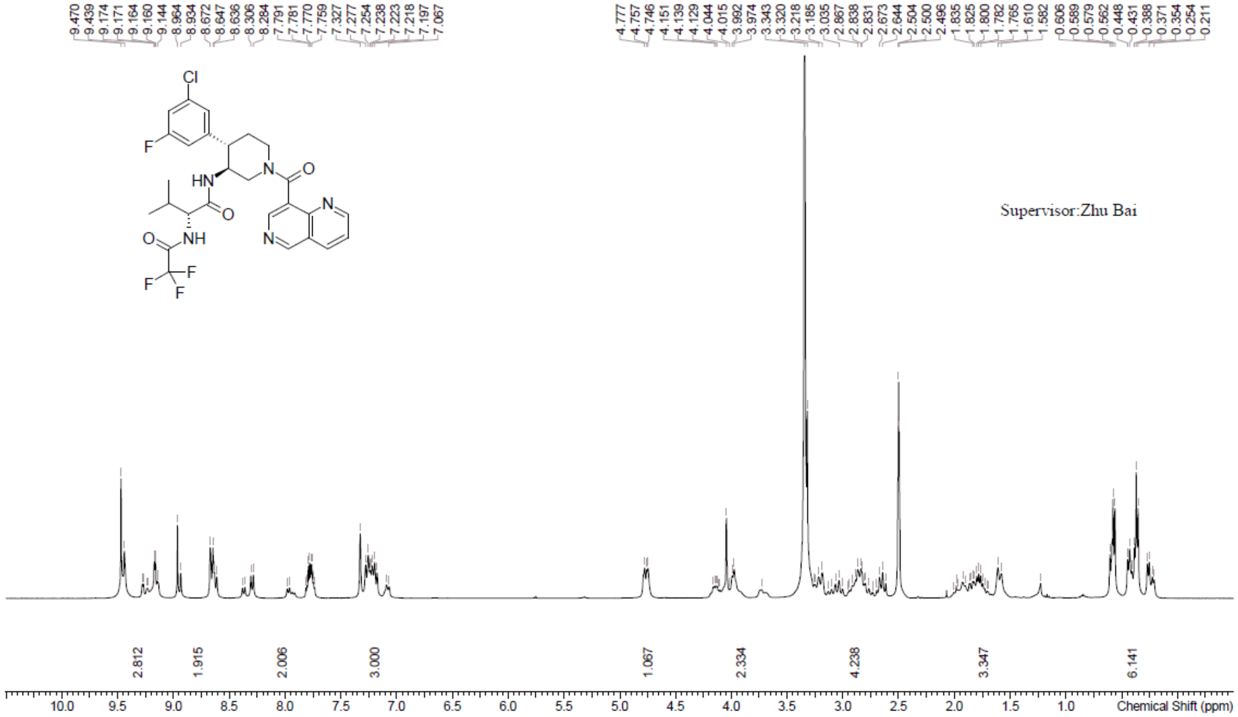
Supervisor:Zhu Bai
Chemical Shift (ppm)

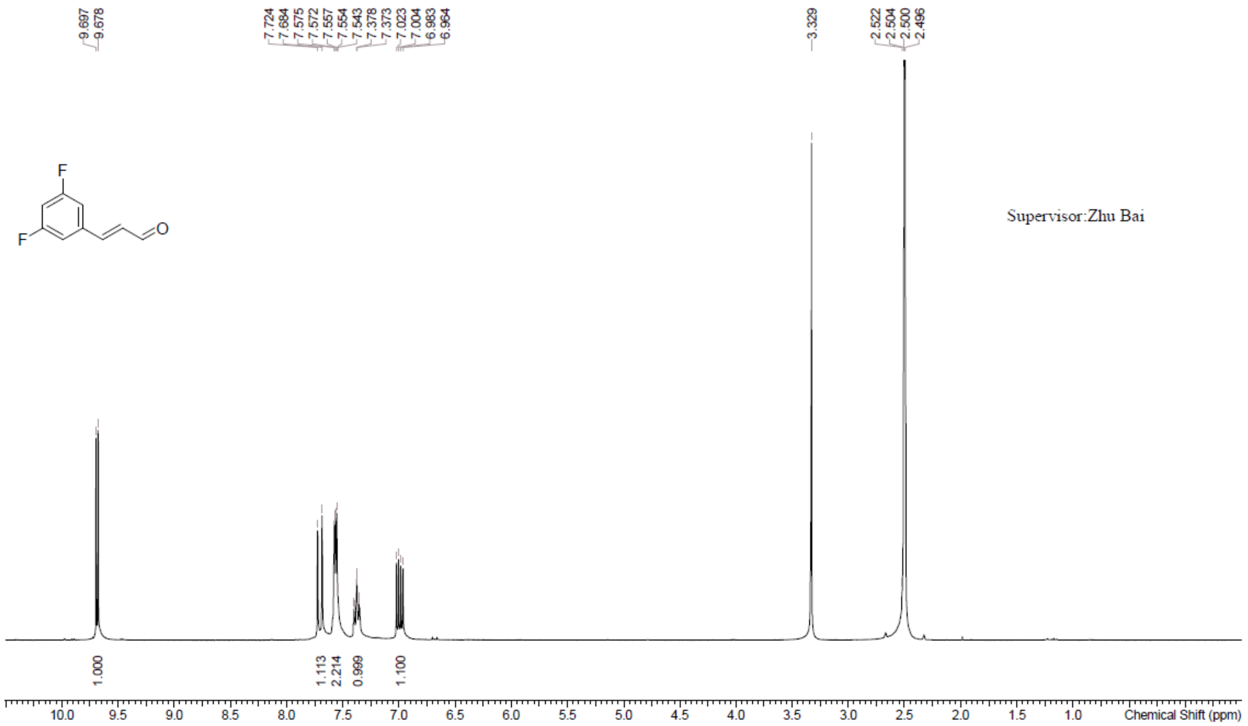
Supervisor:Zhu Bai
Chemical Shift (ppm)

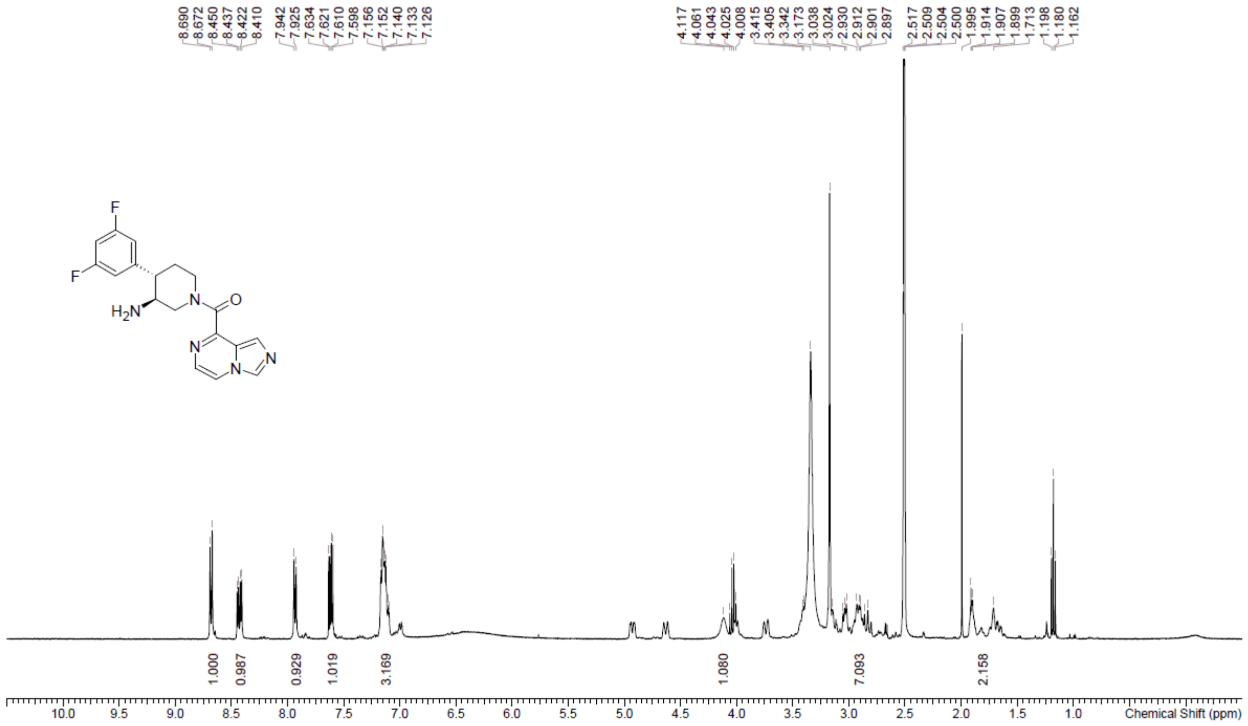
Chemical Shift (ppm)

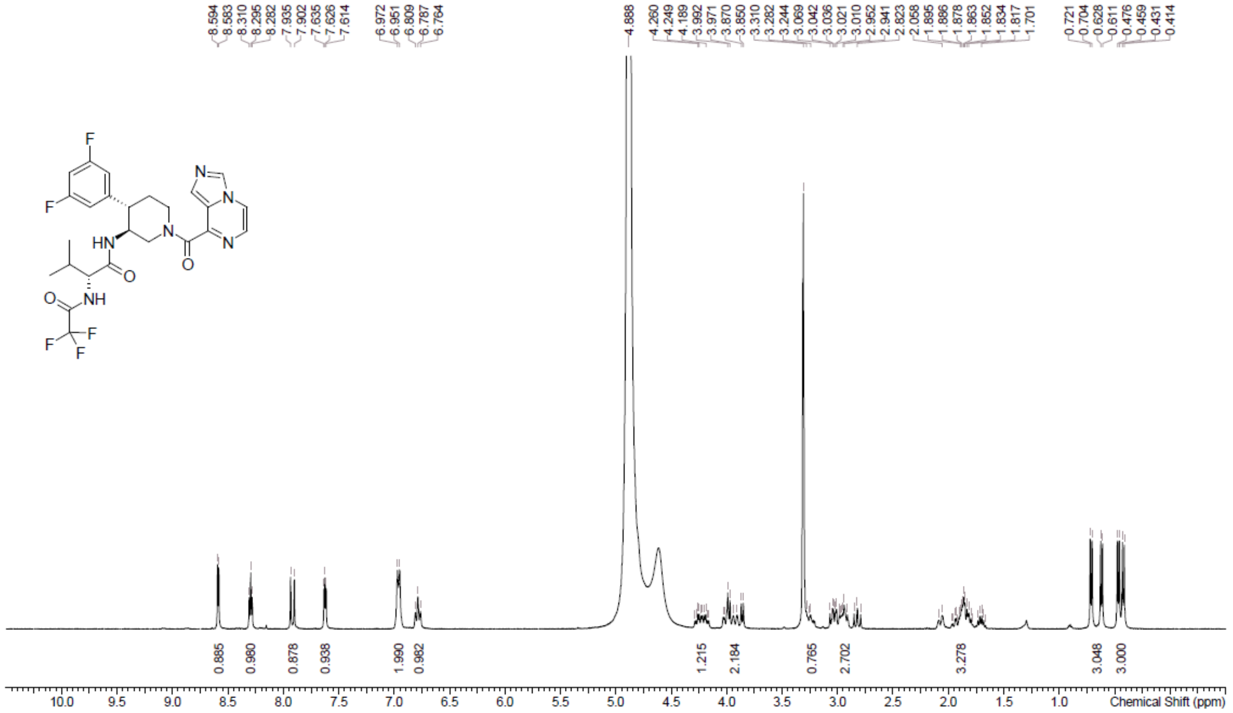
8.594
8.583
8.310
8.295
8.282
7.935
7.902
7.635
7.626
7.614
6.972
6.951
6.809
6.787
6.764
4.888
4.260
4.249
4.189
3.992
3.971
3.870
3.850
3.310
3.282
3.244
3.069
3.042
3.036
3.021
3.010
2.952
2.941
2.823
2.058
1.895
1.886
1.878
1.863
1.852
1.834
1.817
1.701
0.721
0.704
0.628
0.611
0.476
0.459
0.431
0.414
0.885
0.980
0.878
0.938
1.990
0.982
1.215
2.184
0.765
2.702
3.278
3.048
3.000
Chemical Shift (ppm)

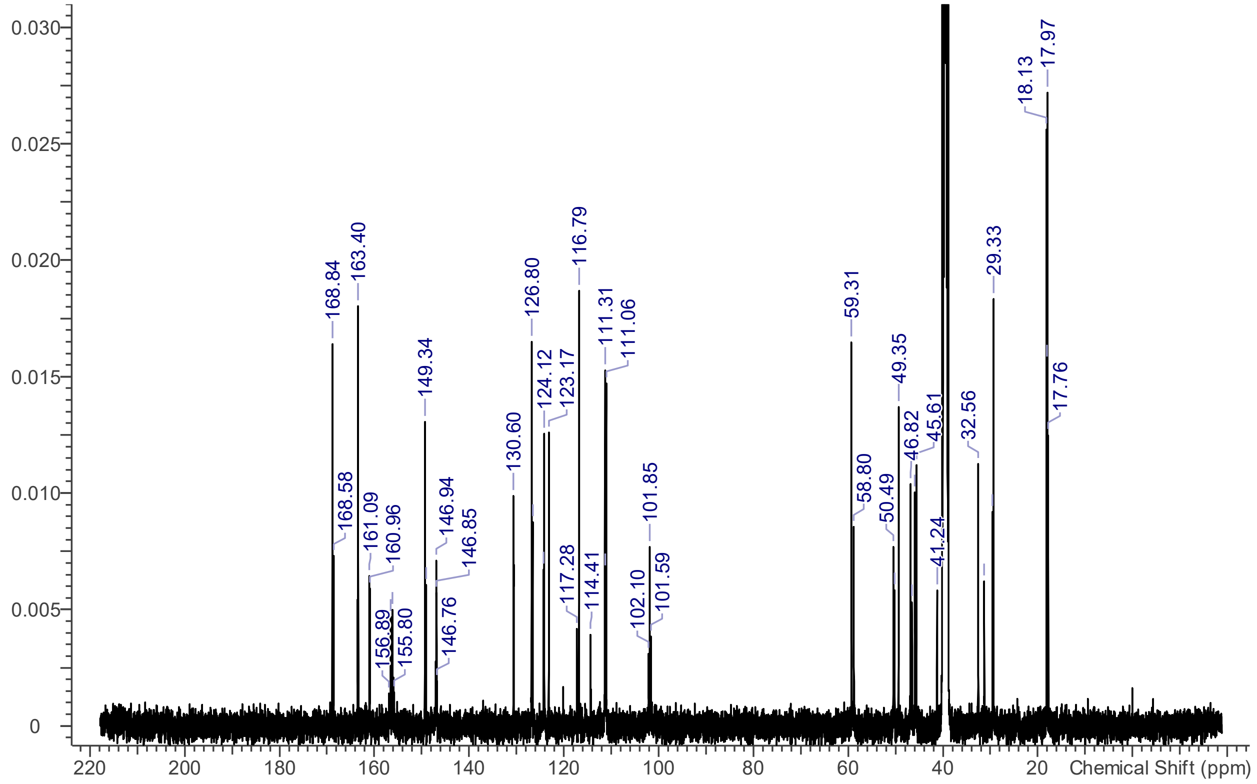
168.84
163.40
168.58
161.09
160.96
156.89
155.80
149.34
146.94
146.85
146.76
130.60
126.80
124.12
123.17
117.28
116.79
114.41
111.31
111.06
102.10
101.85
101.59
59.31
58.80
50.49
49.35
46.82
45.61
41.24
32.56
29.33
18.13
17.97
17.76
Chemical Shift (ppm)

Analytical HPLC of compound 30

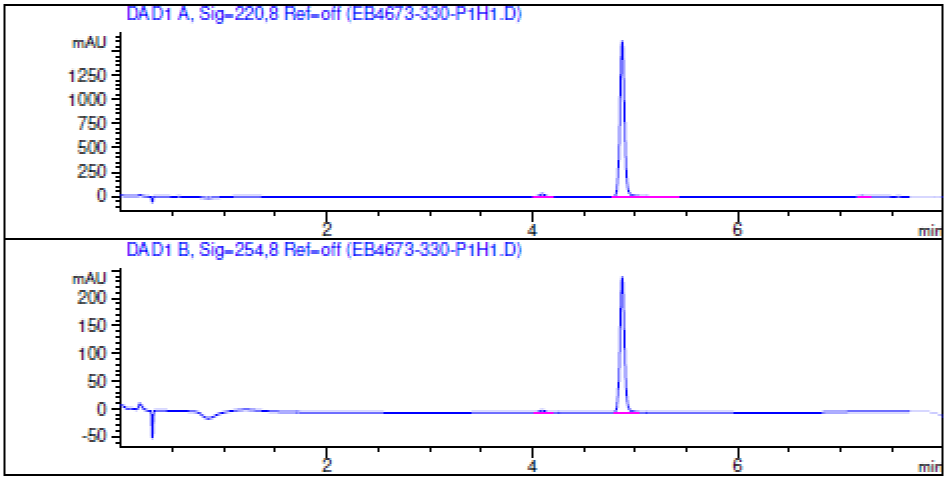

Report

Signal 1 : DAD1 A, Sig=220,8 Ref=off

Peak #	RT [min]	Height	Height %	Width [min]	Area	Area %
1	4.098	29.856	1.814	0.056	109.570	2.080
2	4.876	1605.940	97.555	0.049	5118.342	97.182
3	5.106	3.766	0.229	0.059	16.535	0.314
4	5.244	1.893	0.115	0.057	7.623	0.145
5	5.343	1.607	0.098	0.045	5.226	0.099
6	7.212	3.127	0.190	0.043	9.448	0.179

Signal 2 : DAD1 B, Sig=254,8 Ref=off

Peak #	RT [min]	Height	Height %	Width [min]	Area	Area %
1	4.098	4.623	1.850	0.056	16.942	2.154
2	4.876	245.277	98.150	0.048	769.725	97.846

Chiral SFC of compound 30

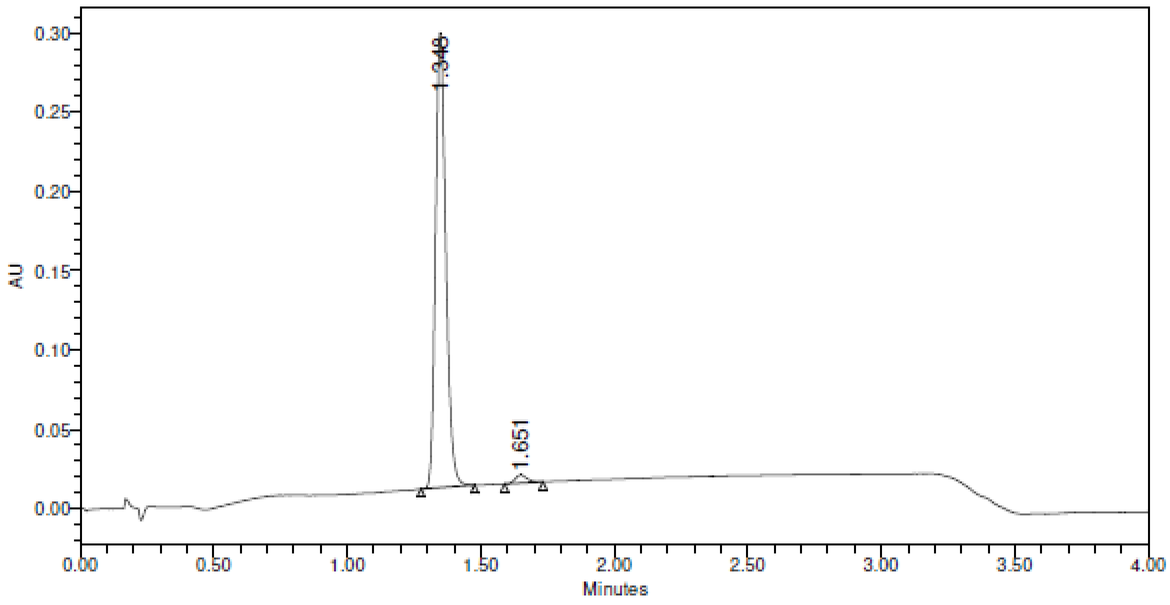

	RT	Area	% Area
1	1.348	772250	98.03
2	1.651	15525	1.97

Experimental procedures for human proteases

Caspase-2 biochemical assay

Compounds were 3-fold serially diluted for 10 doses and added to assay plates (384 well format) using ECHO, in duplicate wells. 250 μM Z-VAD(OMe)-FMK was used as 100% inhibition control, DMSO was used as the no inhibition control. 15 μL of Recombinant human Caspase-2 protein were added to the assay plates containing compounds. The compounds and Recombinant human Caspase-2 protein were pre-incubated at room temperature for 30 min. Then 5μL of Caspase 2 substrate were added to the appropriate assay plates. The final concentrations of protein and substrate were: 50 nM Recombinant human Caspase-2 + 400 μM substrate. Each activity testing point had a relevant background control to normalize the fluorescence interference of compound. After 20 min incubation at 37◦C, the optical density is detected using a microplate reader SpectraMax M2e (Molecular Devices) at 410 nm (OD_{410}). The inhibition activity was calculated using the formula: Inhibition%=[(CPD-BG_{CPD})-(ZPE−BG_{ZPE})]/[(HPE−BG_{HPE})−(ZPE−BG_{ZPE})]*100%; Where CPD: Signal of test compounds wells, containing compound + enzyme + substrate, ZPE: Average of signals of zero percent effective control wells, containing enzyme + substrate, no compound, HPE: Average of signals of hundred percent effect control wells, containing 250 μM Z-VAD(OMe)-FMK + enzyme + substrate, BG: Compound back-ground control wells, containing compound + substrate, no enzyme. The IC_{50} values of compounds were calculated with GraphPad Prism software using the nonlinear regression model of log (inhibitor) vs. response –Variable slope (four parameters).

Chymotrypsin C biochemical assay

Compounds were 3-fold serially diluted for 10 doses and added to assay plates (384 well format) using ECHO, in duplicate wells. 10 μM Chymostatin was used as 100% inhibition control, DMSO was used as the no inhibition control. 15 μL of Recombinant Human Chymotrypsin C protein, which were activated with 0.4 μg/mL Trypsin at 37°C for 1 hour, were added to the assay plates containing compounds. The compounds and Recombinant Human Chymotrypsin C protein were pre-incubated at room temperature for 30 min. Then 5μL of Chymotrypsin C substrate were added to the appropriate assay plates. The final concentrations of protein and substrate were: 62.5 nM Human Chymotrypsin C +200 μM substrate. Each activity testing point had a relevant background control to normalize the fluorescence interference of compound. After 60 min incubation at 37◦C, the optical density is detected using a microplate reader SpectraMax M2e (Molecular Devices) at 410 nm (OD_{410}). The inhibition activity was calculated using the formula: Inhibition%=[(CPD-BG_{CPD})-(ZPE−BG_{ZPE})]/[(HPE−BG_{HPE})−(ZPE−BG_{ZPE})]*100%; Where CPD: Signal of test compounds wells, containing compound + enzyme + substrate, ZPE: Average of signals of zero percent effective control wells, containing enzyme + substrate, no compound, HPE: Average of signals of hundred percent effect control wells, containing 10 μM Chymostatin + enzyme + substrate, BG: Compound back-ground control wells, containing compound + substrate, no enzyme. The IC_{50} values of compounds were calculated with GraphPad Prism software using the nonlinear regression model of log (inhibitor) vs. response –Variable slope (four parameters).

Elastase biochemical assay

Compounds were 3-fold serially diluted for 10 doses and added to assay plates (384 well format) using ECHO, in duplicate wells. 10 μM BAY-678 was used as 100% inhibition control, DMSO was used as the

no inhibition control. 15μL of Recombinant Human Neutrophil Elastase protein, which were activated with 1.25 μM Recombinant Mouse Active Cathepsin C at 37°C for 2 hours, were added to the assay plates containing compounds. The compounds and Recombinant Human Neutrophil Elastase protein were pre-incubated at room temperature for 30 min. Then 5μL of Elastase substrate were added to the appropriate assay plates. The final concentrations of protein and substrate were: 2.5 nM Recombinant Human Neutrophil Elastase +3.125 μM substrate. Each activity testing point had a relevant background control to normalize the fluorescence interference of compound. After 60 min incubation at 37◦C, the fluorescence signal (RFU) is detected using a microplate reader SpectraMax M2e (Molecular Devices) at Ex/Em=380nm/460nm. The inhibition activity was calculated using the formula: Inhibition%=[(CPD-BG_{CPD})-(ZPE−BG_{ZPE})]/ [(HPE−BG_{HPE})−(ZPE−BG_{ZPE})]*100%; Where CPD: Signal of test compounds wells, containing compound + enzyme + substrate, ZPE: Average of signals of zero percent effective control wells, containing enzyme + substrate, no compound, HPE: Average of signals of hundred percent effect control wells, containing 10 μM BAY-678 + enzyme + substrate, BG: Compound back-ground control wells, containing compound + substrate, no enzyme. The IC_{50} values of compounds were calculated with GraphPad Prism software using the nonlinear regression model of log (inhibitor) vs. response –Variable slope (four parameters).

Thrombin biochemical assay

Compounds were 3-fold serially diluted for 10 doses and added to assay plates (384 well format) using ECHO, in duplicate wells. 2 μM Argatroban was used as 100% inhibition control, DMSO was used as the no inhibition control. 15μL of Recombinant Human Thrombin protein were added to the assay plates containing compounds. The compounds and Recombinant Human Thrombin protein were pre-incubated at room temperature for 30 min. Then 5μL of Thrombin substrate were added to the appropriate assay plates. The final concentrations of protein and substrate were: 0.05 nM Recombinant Human Thrombin +12.5 μM substrate. Each activity testing point had a relevant background control to normalize the fluorescence interference of compound. After 60 min incubation at 37◦C, the fluorescence signal (RFU) is detected using a microplate reader SpectraMax M2e (Molecular Devices) at Ex/Em=380nm/460nm. The inhibition activity was calculated using the formula: Inhibition%=[(CPD-BG_{CPD})-(ZPE−BG_{ZPE})]/ [(HPE−BG_{HPE})−(ZPE−BG_{ZPE})]*100%; Where CPD: Signal of test compounds wells, containing compound + enzyme + substrate, ZPE: Average of signals of zero percent effective control wells, containing enzyme + substrate, no compound, HPE: Average of signals of hundred percent effect control wells, containing 2 μM Argatroban + enzyme + substrate, BG: Compound back-ground control wells, containing compound + substrate, no enzyme. The IC_{50} values of compounds were calculated with GraphPad Prism software using the nonlinear regression model of log (inhibitor) vs. response –Variable slope (four parameters).

Cathepsin D biochemical assay

Compounds were 3-fold serially diluted for 10 doses and added to assay plates (384 well format) using ECHO, in duplicate wells. 10 nM Pepstatin A was used as 100% inhibition control, DMSO was used as the no inhibition control. 15μL of Recombinant human Cathepsin D protein, which were activated in 37°C water bath for 30 min, were added to the assay plates containing compounds. The compounds and Recombinant human Cathepsin D protein were pre-incubated at room temperature for 30 min. Then 5μL of Cathepsin D substrate were added to the appropriate assay plates. The final concentrations of protein and substrate were: 5 nM Recombinant human Cathepsin D +6.25 μM substrate. Each activity testing

point had a relevant background control to normalize the fluorescence interference of compound. After 40 min incubation at 37◦C, the fluorescence signal (RFU) is detected using a microplate reader SpectraMax M2e (Molecular Devices) at Ex/Em=320nm/405nm. The inhibition activity was calculated using the formula: Inhibition%=[(CPD-BG_{CPD})-(ZPE−BG_{ZPE})]/ [(HPE−BG_{HPE})−(ZPE−BG_{ZPE})]*100%; Where CPD: Signal of test compounds wells, containing compound + enzyme + substrate, ZPE: Average of signals of zero percent effective control wells, containing enzyme + substrate, no compound, HPE: Average of signals of hundred percent effect control wells, containing 10 nM Pepstatin A + enzyme + substrate, BG: Compound back-ground control wells, containing compound + substrate, no enzyme. The IC_{50} values of compounds were calculated with GraphPad Prism software using the nonlinear regression model of log (inhibitor) vs. response –Variable slope (four parameters).

Pepsin biochemical assay

Compounds were 3-fold serially diluted for 10 doses and added to assay plates (384 well format) using ECHO, in duplicate wells. 100 nM Pepstatin A was used as 100% inhibition control, DMSO was used as the no inhibition control. 15μL of Pepsin protein were added to the assay plates containing compounds. The compounds and Pepsin protein were pre-incubated at room temperature for 30 min. Then 5μL of Pepsin substrate were added to the appropriate assay plates. The final concentrations of protein and substrate were: 1.25 nM Pepsin +12.5 μM substrate. Each activity testing point had a relevant background control to normalize the fluorescence interference of compound. After 40 min incubation at 37◦C, the fluorescence signal (RFU) is detected using a microplate reader SpectraMax M2e (Molecular Devices) at Ex/Em=320nm/405nm. The inhibition activity was calculated using the formula: Inhibition%=[(CPD-BG_{CPD})-(ZPE−BG_{ZPE})]/ [(HPE−BG_{HPE})−(ZPE−BG_{ZPE})]*100%; Where CPD: Signal of test compounds wells, containing compound + enzyme + substrate, ZPE: Average of signals of zero percent effective control wells, containing enzyme + substrate, no compound, HPE: Average of signals of hundred percent effect control wells, containing 100 nM Pepstatin A + enzyme + substrate, BG: Compound back-ground control wells, containing compound + substrate, no enzyme. The IC_{50} values of compounds were calculated with GraphPad Prism software using the nonlinear regression model of log (inhibitor) vs. response –Variable slope (four parameters).

Cathepsin B biochemical assay

Compounds were 3-fold serially diluted for 10 doses and added to assay plates (384 well format) using ECHO, in duplicate wells. 5 μM Leupeptin hemisulfate salt was used as 100% inhibition control, DMSO was used as the no inhibition control. 25 μL of Cathepsin B were added to the corresponding assay plates containing compounds respectively. The compounds and protein were pre-incubated at room temperature for 30 min. Then 5μL of substrate were added to the appropriate assay plates. The final concentrations of protein and substrate were: 1.17 nM Cathepsin B + 15 μM substrate. Each activity testing point had a relevant background control to normalize the fluorescence interference of compound. After 30 min incubation at 37◦C, the fluorescence signal (RFU) is detected using a microplate reader SpectraMax M2e (Molecular Devices) at Ex/Em=348nm/440nm. The inhibition activity was calculated using the formula: Inhibition%=[(CPD-BG_{CPD})-(ZPE−BG_{ZPE})]/ [(HPE−BG_{HPE})−(ZPE−BG_{ZPE})]*100%; Where CPD: Signal of test compounds wells, containing compound + enzyme + substrate, ZPE: Average of signals of zero percent effective control wells, containing enzyme + substrate, no compound, HPE: Average of signals of hundred percent effect control wells, containing 5 μM Leupeptin hemisulfate salt + enzyme + substrate, BG: Compound back-ground control wells, containing compound + substrate, no enzyme. The IC_{50} values

of compounds were calculated with GraphPad Prism software using the nonlinear regression model of log (inhibitor) vs. response –Variable slope (four parameters).

Cathepsin K biochemical assay

Compounds were 3-fold serially diluted for 10 doses and added to assay plates (384 well format) using ECHO, in duplicate wells. 5 µM Leupeptin hemisulfate salt was used as 100% inhibition control, DMSO was used as the no inhibition control. 25 µL of Cathepsin K were added to the corresponding assay plates containing compounds respectively. The compounds and protein were pre-incubated at room temperature for 30 min. Then 5µL of substrate were added to the appropriate assay plates. The final concentrations of protein and substrate were: 3.2 nM Cathepsin K and 12.5 µM substrate. Each activity testing point had a relevant background control to normalize the fluorescence interference of compound. After 60 min incubation at 37◦C, the fluorescence signal (RFU) is detected using a microplate reader SpectraMax M2e (Molecular Devices) at Ex/Em=360nm/460nm. The inhibition activity was calculated using the formula: Inhibition%=$[(CPD-BG_{CPD})-(ZPE-BG_{ZPE})]/ [(HPE-BG_{HPE})-(ZPE-BG_{ZPE})]*100\%$; Where CPD: Signal of test compounds wells, containing compound + enzyme + substrate, ZPE: Average of signals of zero percent effective control wells, containing enzyme + substrate, no compound, HPE: Average of signals of hundred percent effect control wells, containing 5 µM Leupeptin hemisulfate salt + enzyme + substrate, BG: Compound back-ground control wells, containing compound + substrate, no enzyme. The IC_{50} values of compounds were calculated with GraphPad Prism software using the nonlinear regression model of log (inhibitor) vs. response –Variable slope

Cathepsin L biochemical assay

Compounds were 3-fold serially diluted for 10 doses and added to assay plates (384 well format) using ECHO, in duplicate wells. 5 µM Leupeptin hemisulfate salt was used as 100% inhibition control, DMSO was used as the no inhibition control. 25 µL of Cathepsin L were added to the corresponding assay plates containing compounds respectively. The compounds and protein were pre-incubated at room temperature for 30 min. Then 5µL of substrate were added to the appropriate assay plates. The final concentrations of protein and substrate were: 1 nM Cathepsin L and 6.25 µM substrate. Each activity testing point had a relevant background control to normalize the fluorescence interference of compound. For Cathepsin L assay, After 60 min incubation at 25◦C, the fluorescence signal (RFU) is detected using a microplate reader SpectraMax M2e (Molecular Devices) at Ex/Em=360nm/460.The inhibition activity was calculated using the formula: Inhibition%=$[(CPD-BG_{CPD})-(ZPE-BG_{ZPE})]/ [(HPE-BG_{HPE})-(ZPE-BG_{ZPE})]*100\%$; Where CPD: Signal of test compounds wells, containing compound + enzyme + substrate, ZPE: Average of signals of zero percent effective control wells, containing enzyme + substrate, no compound, HPE: Average of signals of hundred percent effect control wells, containing 5 µM Leupeptin hemisulfate salt + enzyme + substrate, BG: Compound back-ground control wells, containing compound + substrate, no enzyme. The IC_{50} values of compounds were calculated with GraphPad Prism software using the nonlinear regression model of log (inhibitor) vs. response –Variable slope

Cathepsin S biochemical assay

Compounds were 3-fold serially diluted for 10 doses and added to assay plates (384 well format) using ECHO, in duplicate wells. 5 μM Leupeptin hemisulfate salt was used as 100% inhibition control, DMSO was used as the no inhibition control. 25 μL of Cathepsin S were added to the corresponding assay plates containing compounds respectively. The compounds and protein were pre-incubated at room temperature for 30 min. Then 5μL of substrate were added to the appropriate assay plates. The final concentrations of protein and substrate were: 5 nM Cathepsin S and 25 μM substrate. Each activity testing point had a relevant background control to normalize the fluorescence interference of compound. After 60 min incubation at 37◦C, the fluorescence signal (RFU) is detected using a microplate reader SpectraMax M2e (Molecular Devices) at Ex/Em=360nm/460nm. The inhibition activity was calculated using the formula: Inhibition%=[(CPD-BG_{CPD})-(ZPE−BG_{ZPE})]/ [(HPE−BG_{HPE})−(ZPE−BG_{ZPE})]*100%; Where CPD: Signal of test compounds wells, containing compound + enzyme + substrate, ZPE: Average of signals of zero percent effective control wells, containing enzyme + substrate, no compound, HPE: Average of signals of hundred percent effect control wells, containing 5 μM Leupeptin hemisulfate salt + enzyme + substrate, BG: Compound back-ground control wells, containing compound + substrate, no enzyme. The IC_{50} values of compounds were calculated with GraphPad Prism software using the nonlinear regression model of log (inhibitor) vs. response –Variable slope

Experimental procedures for *in vitro* safety

hERG inhibition assay

Whole-cell patch-clamp experiments were conducted using Chinese hamster ovary (CHO) cells expressing the human ether-a-go-go-related gene channel with SyncroPatch 384 (Nanion Technologies, Germany) at room temperature. Single-hole chips with medium resistance were used. External solution contained 80 mM NaCl, 4mM KCl, 2mM CaCl2 2, 1mM MgCl2, 5mM Glucose, 60 mM NMDG 60, 10 mM HEPES 10, adjusted pH to pH7.4. Internal solution contained 110 mM KF, 10 mM KCl, 10 mM NaCl, 10 mM EGTA, 10 mM HEPES, adjusted pH to pH7.2. The hERG current is elicited using the following voltage protocol. From the holding potential of -80 mV, the voltage was first stepped to -90 mV for 100 ms and back to -80 mV. After that the voltage was stepped to +40 mV for 500 ms and then ramped to -80 mV for 108 ms, causing a "rebound" current, which was measured and collected for data analysis. Finally, the voltage was stepped back to the holding potential. This voltage command protocol was repeated every 5000 msec during the test. Two additions of 40 µL of DMSO were applied as the vehicle, followed by 300s at least of voltage protocol for a baseline period. Then each concentration of compound30 was added and current response was recorded. Within each cell recording, percent of control values were calculated for each test concentration current response based on peak current in presence of vehicle control (current response/ peak current) ×100%. For four concentrations testing, IC50 values were determined from Dose-Response curves that were obtained with the standard Hill equation as shown below:

Y=Bottom+(Top-Bottom)/(1+10^((LogIC50-X)*HillSlope))

Where X is the logarithm of concentration, Y is inhibition%, Top is 1 and Bottom is equal to 0.

Nav1.5 inhibition assay

Whole-cell patch-clamp experiments were conducted using HEK293 cells expressing Nav1.5 sodium channel with SyncroPatch 384 (Nanion Technologies, Germany) at room temperature. Single-hole chips with low resistance were used. External solution contained 140 mM NaCl, 4mM KCl, 3 mM CaCl2 , 1 mM MgCl2, 5 mM Glucose, 10 mM HEPES, adjusted pH to 7.4. Internal solution contained 110 mM CsF, 10 mM CsCl, 10 mM NaCl, 10 mM EGTA, 10 mM HEPES, adjusted pH to pH 7.2. hNav1.5 current is elicited using the following voltage protocol. From the holding potential of -95 mV, the voltage was first stepped back to -120 mV for 200 ms. After that, the voltage was stepped to -15 mV for 40 ms to open the sodium channels. The fast currents induced under the pulse -15 mV were for the data analysis. And then the voltage was stepped to +40 mV for 200 ms and ramped from +40 mV to -95 mV with 108 ms duration. After that, the voltage was stepped at -95 mV holding potential. This voltage command protocol was repeated every 10 s continuously during the test. One addition of 40 µL of DMSO were applied as the vehicle, followed by 300s at least of voltage protocol for a baseline period. Then each concentration of compound30 was added and current response was recorded. Within each cell recording, percent of control values were calculated for each test concentration current response based on peak current in presence of vehicle control (current response/ peak current) ×100%. For four concentrations testing, IC50 values were determined from Dose-Response curves that were obtained with the standard Hill equation as shown below:

Y=Bottom+(Top-Bottom)/(1+10^((LogIC50-X)*HillSlope))

Where X is the logarithm of concentration, Y is inhibition%, Top is 1 and Bottom is equal to 0.

In vitro micronucleus test

An in vitro micronucleus test was conducted using TK6 cells (human lymphoblast-derived) with short-term treatment involving a metabolic activation system (S9 mix) and continuous treatment without S9 mix. A suspension of TK6 cells was utilized either in the absence of metabolic activation for the 24-hour treatment group or in the presence of metabolic activation for the 4-hour treatment group. The suspensions were mixed with compound30 dissolved in DMSO solution and then incubated at 37°C for the respective treatment periods. Both negative controls (DMSO) and positive controls (cyclophosphamide monohydrate or colchicine) were prepared concurrently. Following the short-term treatment period, cells were washed and subsequently incubated in fresh culture medium for 20 hours. Post-incubation, cells were counted to assess cytotoxicity, and the in vitro micronucleus test was performed with compound30 at doses ranging from 222-500 µg/mL for both short-term and continuous treatments based on cytotoxicity data. Nucleic acid staining was achieved using the Giemsa staining method, and micronucleus frequency was observed under a microscope. The test result was considered positive if there was a significant and dose-dependent increase in the number of micronuclei-containing cells in the test-substance groups compared to the negative-control group under any treatment condition.

lnSphero's 3D lnSightTM Human Liver models test

Human 3D InSight Liver Models, hLiMT, was created by cryopreserved primary human hepatocytes (Lot IPHH_32; multi-donor) with cryopreserved primary human nonparenchymal cells (Lot IPHN_17; single donor). The culture medium was 3D InSight Human Liver Maintenance Medium TOX (Catalogue No. CS-07-001, InSphero). The treated cells in 96-well Akura™ plates were incubated in a humidified cell culture incubator (37°C, 5% CO2). hLiMTs were exposed to compound30 (0.1, 0.3, 1, 3.16, 10, 31.6, or 100 µM in 0.5% DMSO) for 7 days (compound30 administered on Days 0 and 4). Six replicates were tested for each condition. The vehicle control was 0.5% DMSO, and the positive control was chlorpromazine (0.1, 0.3, 1, 3.16, 10, 31.6, or 100 µM in DMSO). Cellular ATP content was measured in the cell lysate on Days 7 of treatment. Intracellular ATP content in model lysates was measured with the CellTiter-Glo® 2.0 Cell Viability Assay (Catalogue No. G9243, Promega).
